# Risk of Infection Associated With Administration of Intravenous Iron: A Systematic Review and Meta-analysis

**DOI:** 10.1001/jamanetworkopen.2021.33935

**Published:** 2021-11-12

**Authors:** Akshay A. Shah, Killian Donovan, Claire Seeley, Edward A. Dickson, Antony J. R. Palmer, Carolyn Doree, Susan Brunskill, Jack Reid, Austin G. Acheson, Anita Sugavanam, Edward Litton, Simon J. Stanworth

**Affiliations:** 1 Radcliffe Department of Medicine, University of Oxford, Oxford, United Kingdom; 2 National Institute for Health Research Biomedical Research Centre Haematology Theme, Oxford, United Kingdom; 3 Adult Intensive Care Unit, Oxford University Hospitals National Health Service (NHS) Foundation Trust, Oxford, United Kingdom; 4 Department of Anaesthesia, Royal Berkshire Hospitals NHS Foundation Trust, Reading, United Kingdom; 5 National Institute for Health Research Biomedical Research Centre in Gastrointestinal and Liver Diseases, Nottingham University Hospitals NHS Trust, Nottingham, United Kingdom.; 6 Department of Colorectal Surgery, Nottingham University Hospitals NHS Trust, University of Nottingham, Nottingham, United Kingdom.; 7 Nuffield Department of Orthopaedics, Rheumatology and Musculoskeletal Sciences, University of Oxford, Oxford, United Kingdom; 8 Systematic Review Initiative, NHS Blood & Transplant, Oxford, United Kingdom; 9 Department of Anaesthesia, Brighton and Sussex University Hospitals NHS Trust, Brighton, United Kingdom; 10 Intensive Care Unit, Fiona Stanley Hospital, Perth, Australia; 11 Department of Haematology, Oxford University Hospitals NHS Foundation Trust, Oxford, United Kingdom

## Abstract

**Importance:**

Intravenous iron is recommended by many clinical guidelines based largely on its effectiveness in reducing anemia. However, the association with important safety outcomes, such as infection, remains uncertain.

**Objective:**

To examine the risk of infection associated with intravenous iron compared with oral iron or no iron.

**Data Sources:**

Medline, Embase, and Cochrane Central Register of Controlled Trials (CENTRAL) were searched for randomized clinical trials (RCTs) from 1966 to January 31, 2021. Ongoing trials were sought from ClinicalTrials.gov, CENTRAL, and the World Health Organization International Clinical Trials Search Registry Platform.

**Study Selection:**

Pairs of reviewers identified RCTs that compared intravenous iron with oral iron or no iron across all patient populations, excluding healthy volunteers. Nonrandomized studies published since January 1, 2007, were also included. A total of 312 full-text articles were assessed for eligibility.

**Data Extraction and Synthesis:**

Data extraction and risk of bias assessments were performed according to the Preferred Reporting Items of Systematic Reviews and Meta-analyses (PRISMA) and Cochrane recommendations, and the quality of evidence was assessed using the GRADE (Grades of Recommendation, Assessment, Development, and Evaluation) approach. Two reviewers extracted data independently. A random-effects model was used to synthesize data from RCTs. A narrative synthesis was performed to characterize the reporting of infection.

**Main Outcomes and Measures:**

The primary outcome was risk of infection. Secondary outcomes included mortality, hospital length of stay, and changes in hemoglobin and red blood cell transfusion requirements. Measures of association were reported as risk ratios (RRs) or mean differences.

**Results:**

A total of 154 RCTs (32 762 participants) were included in the main analysis. Intravenous iron was associated with an increased risk of infection when compared with oral iron or no iron (RR, 1.16; 95% CI, 1.03-1.29; *I*^2^ = 36%; moderate certainty of evidence). Intravenous iron also was associated with an increase in hemoglobin (mean difference, 0.57 g/dL; 95% CI, 0.50-0.64 g/dL; *I*^2^ = 94%) and a reduction in the risk of requiring a red blood cell transfusion (RR, 0.83; 95% CI, 0.76-0.89; *I*^2^ = 15%) when compared with oral iron or no iron. There was no evidence of an effect on mortality or hospital length of stay.

**Conclusions and Relevance:**

In this large systematic review and meta-analysis, intravenous iron was associated with an increased risk of infection. Well-designed studies, using standardized definitions of infection, are required to understand the balance between this risk and the potential benefits.

## Key Points

**Question:**

In patients who require treatment with intravenous iron, what is the evidence that this intervention increases the risk of developing a new infection?

**Findings:**

In this systematic review and meta-analysis of 154 randomized clinical trials that included 32 762 participants, intravenous iron was associated with an increased risk of infection.

**Meaning:**

The results of this study suggest that, despite broad advocacy in clinical guidelines, intravenous iron may increase the risk of infection, which must be balanced with the potential benefits of treating anemia and reducing blood transfusion requirements.

## Teaser

This systematic review and meta-analysis evaluates the risk of infection associated with intravenous iron vs oral iron or no iron.

## Introduction

Intravenous iron is recommended by clinical practice guidelines for the treatment of anemia associated with a range of common conditions, including chronic kidney disease (CKD), heart failure, and inflammatory bowel disease (IBD). During the past decade, its use has expanded to other clinical settings where rapid replenishment of iron stores is required or where oral iron may not be absorbed (eg, chronic inflammation, surgery, and obstetrics).^1,2,3,4,5,6,7^ This expansion may, in part, be driven by newer preparations that are reported to be safer with regard to risks such as anaphylaxis when compared with older dextran-based formations,^8^ ease of administration, and increased adoption of patient blood management programs.

Iron is required for host immunity and pathogen replication, and in health, iron is tightly regulated by the peptide hormone hepcidin.^9,10,11^ Inflammation triggers a process of withholding free iron from invading pathogens, termed *nutritional immunity*.^11,12^ Intravenous iron increases the levels of circulating non–transferrin-bound iron, which may be detrimental to the host by promoting pathogen growth^13^ and predisposes patients to infection. Recent data suggest that the use of intravenous iron is increasing.^14,15,16^ Therefore, there is an unmet need to examine whether there is an association between intravenous iron and infection and, if so, whether this association increases morbidity and mortality.^17,18,19,20^

Previous systematic reviews^21,22^ have demonstrated the efficacy of intravenous iron for treating anemia and reducing allogeneic red blood cell (RBC) transfusions, but safety data on the risk of infection are conflicting. A recent trial^23^ also questioned the benefit of intravenous iron in patients undergoing major surgery. A systematic review^24^ of 35 international guidelines on the use of intravenous iron across multiple indications found that approximately 60% of the guidelines have not been updated in more than 5 years and consequently do not reflect the current evidence on the safety and efficacy of intravenous iron. Therefore, we aimed to evaluate the safety and efficacy of intravenous iron by updating a previous systematic review^24^ and focusing primarily on the risk of infection.

## Methods

This report was prepared according to the Preferred Reporting Items for Systematic Reviews and Meta-analyses (PRISMA) reporting guideline. The study protocol was registered with PROSPERO (CRD42018096023) and has been published previously.^25^

### Eligibility Criteria

We included randomized clinical trials (RCTs) that compared intravenous iron with oral iron or no iron or placebo. We included studies from 1966 to January 31, 2021, that were included in the previous meta-analysis and conducted another search of studies published from January 1, 2013, until January 5, 2021, examining all relevant patient populations. We recognized that adverse events may not always be fully captured in RCTs.^26^ Therefore, we also searched for nonrandomized studies (NRSs) that met the following criteria: (1) report data on infection, (2) presence of at least 2 comparable groups, (3) quasi-RCTs, and (4) published since January 1, 2007. This date was chosen because this was the year that newer iron preparations (eg, ferric carboxymaltose and iron isomaltoside) received or renewed their marketing authorization.

Our primary outcome was the risk of infection. Secondary outcomes were differences in hemoglobin, RBC transfusion requirements, hospital length of stay, and short-term (≤30 days) and long-term (>30 days) mortality.

### Search Methods and Study Selection

An information specialist (C.D.) searched the following databases: Cochrane Central Register of Controlled Trials (CENTRAL) in The Cochrane Library, Medline, Embase, Cumulative Index to Nursing and Allied Health Literature (CINAHL), PubMed (e-publications ahead of print only), Web of Science (1990 onward), and the Transfusion Evidence Library (1950 onward). Ongoing trials were sought from ClinicalTrials.gov, CENTRAL, and World Health Organization International Clinical Trials Search Registry Platform. Key search terms included *iron, ferric/ferrous compounds,* and *intravenous/I.V./inject/parenteral.* Only English-language articles were considered. The first search date was May 2, 2018, and the last search date was January 5, 2021. The full search strategy is available in eAppendix 1 in the Supplement.

Literature searches were uploaded to Covidence, a web-based software platform, and 2 reviewers (A.A.S. and K.D.) independently screened study titles and abstracts for eligibility. The same 2 reviewers then independently screened the full text reports for inclusion. Disagreements were resolved through discussion.

### Data Extraction

Data were extracted onto standardized and prepiloted forms for RCTs and NRSs by pairs of 2 reviewers (A.A.S. and K.D. as well as C.S. and E.A.D.) independently. The following data, if reported, on the outcome of infection were extracted: (1) definitions of infection used, (2) anatomical site of infection, (3) positive microbiology culture data, and (4) antibiotic use. Disagreements were resolved through discussion or referred to a third reviewer (S.J.S.). Study authors were contacted to resolve any uncertainties.

### Assessment of Risk of Bias

Risk of bias for RCTs was assessed using the Cochrane Risk of Bias tool.^27^ Because of some of the potential costs and logistical difficulties associated with blinding intravenous iron preparations due to their rusty brown appearance, we made an a priori decision to classify open-label designs as low risk for detection bias for objective outcomes, such as changes in hemoglobin, mortality, and length of stay, in the absence of other concerns. For analysis and presentation purposes, a 3-point scale (low risk, some concerns, or high risk) was used to determine overall risk of bias for each study. Risk of bias for NRSs was assessed using the Risk of Bias in Nonrandomised Studies–of Interventions (ROBINS-I) tool developed by the Cochrane Bias Methods Group.^28^ All assessments were performed in duplicate by pairs of 2 independent reviewers (A.A.S. and K.D. as well as C.S. and E.A.D.).

### Data Synthesis

The primary end point was the proportion of patients who developed an infection. Dichotomous outcomes (infection, mortality, and requirement for RBC transfusion) were reported as risk ratio (RR) with corresponding 95% CIs. For continuous measures, we reported mean differences (MD) with 95% CIs. Data from RCTs that fulfilled the eligibility criteria were pooled for meta-analysis with a random-effects model. Statistical heterogeneity was measured using the *I*^2^ statistic, and *I*^2^ > 50% was considered as showing substantial heterogeneity.^29^ Metaregression was undertaken to investigate the effect of baseline iron status (hemoglobin, ferritin, and transferrin saturation) on the risk of developing infection. Statistical analyses were conducted with RevMan software, version 5.3 (Cochrane Collaboration) and Stata software, version 14 (StataCorp LLC). A 2-sided *P* < .05 was considered statistically significant. For NRSs, we reported results on infection descriptively instead of pooling results because of heterogeneity in clinical conditions, study designs, and differences in adjusting for confounders.^28^ A narrative synthesis was performed to characterize the definitions of infection used, reporting of infection sources and pathogens, and antibiotic use.

#### Subgroup and Sensitivity Analyses

We hypothesized that certain clinical, biological, and therapeutic characteristics may predispose patients to an increased risk of infection. If sufficient data were available, we planned a priori to perform subgroup analyses on the following: (1) clinical settings or conditions (surgery, obstetrics, critical illness, CKD, IBD, heart failure, and pediatrics), (2) iron profiles at enrollment, (3) mode of administration (single dose or multiple doses), and (4) iron preparation used and cumulative dose of iron. We performed a sensitivity analysis for infection by only including trials judged to be at overall low risk of bias.

#### Assessment of the Quality of the Evidence

The GRADE (Grades of Recommendation, Assessment, Development, and Evaluation)^30^ approach was used to assess the overall quality of evidence for the primary outcome of infection. We assessed for publication bias on the primary outcome with a funnel plot if more than 10 RCTs were available, plotting the odds ratio for the proportion that develop infection against the SE of the log odds ratio.

## Results

### Study Selection

**Figure 1.**
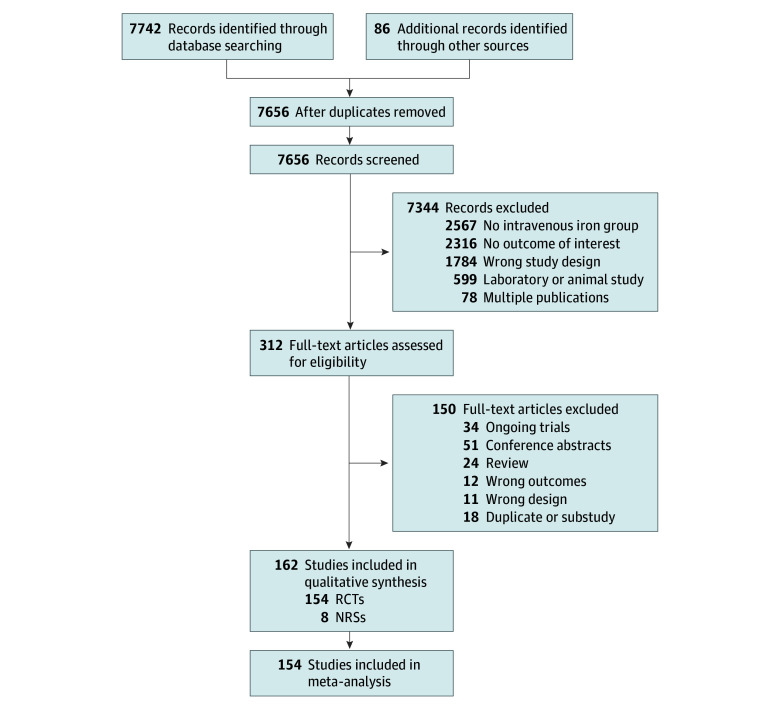
Selection of Studies in the Systematic Review NRSs indicates nonrandomized studies; RCTs, randomized clinical trials.

Our search identified 7656 records. We assessed 311 full-text articles for exclusion by screening of titles, duplicates, and abstracts (Figure 1). Details on all included and the 34 ongoing trials are available (Table 1; eTables 1 and 2 in the Supplement).

**Table 1.** Summary Characteristics of Included RCTs According to Geographic Location, Clinical Settings, and Type of Iron Preparation

Characteristic	No. of RCTs		
	Intravenous iron vs oral iron	Intravenous iron vs no iron	Total
Single center	51	30	81
Multicenter	37	36	73
Geographic location			
Australasia	5	4	9
Europe	25	31	56
Asia	28	14	42
Africa	4	0	4
North America	17	16	33
South America	2	1	3
Cross-continent	6	1	7
Clinical setting			
Blood donors	1	2	3
CCF	1	8	9
CKD	16	6	22
Critical care	0	5	5
Elite athletes	0	2	2
IBD	4	2	6
Mixed or general medical	2	7	9
Menorrhagia	1	2	3
Obstetrics	39	0	39
Oncology			
Hematology	0	3	3
Nonhematology or solid organ	8	5	13
Pediatric	3	1	4
Restless legs	0	7	7
Surgery	10	17	27
Gastrointestinal bleeding	0	2	2
Inclusion criteria			
Iron deficiency anemia	28	9	37
Functional iron deficiency	20	14	34
Hemoglobin only	31	25	56
Iron deficiency only	3	7	10
Nonanemic	4	2	6
Unclear	2	9	11
Iron preparations evaluated			
Ferric carboxymaltose	21	30	51
Iron sucrose	43	25	68
Iron dextran	8	4	12
Iron isomaltoside	4	3	7
Iron polymaltose	3	0	3
Iron saccharate	2	0	2
Ferumoxytol	2	1	3
Ferric gluconate	3	4	7
Not reported	1	0	1

Abbreviations: CCF, congestive cardiac failure; CKD, chronic kidney disease; IBD, inflammatory bowel disease; RCT, randomized clinical trial.

### Study Characteristics

A total of 162 studies with 39 908 participants were included in this systematic review, of which 154 were RCTs (32 762 participants)^31,32,33,34,35,36,37,38,39,40,41,42,43,44,45,46,47,48,49,50,51,52,53,54,55,56,57,58,59,60,61,62,63,64,65,66,67,68,69,70,71,72,73,74,75,76,77,78,79,80,81,82,83,84,85,86,87,88,89,90,91,92,93,94,95,96,97,98,99,100,101,102,103,104,105,106,107,108,109,110,111,112,113,114,115,116,117,118,119,120,121,122,123,124,125,126,127,128,129,130,131,132,133,134,135,136,137,138,139,140,141,142,143,144,145,146,147,148,149,150,151,152,153,154,155,156,157,158,159,160,161,162,163,164,165,166,167,168,169,170,171,172,173,174,175,176,177,178,179,180,181,182,183,184^ and 8 were NRSs (7146 participants).^185,186,187,188,189,190,191,192^ Two RCTs^47,106^ were analyzed as 2 separate studies each. A descriptive summary, including study characteristics and risk of bias assessments, of NRSs that reported on outcomes of infection is provided in eAppendix 2 and eTables 3 and 4 in the Supplement.

The median number of participants in the included RCTs was 111 (IQR, 14-2534). Seventy-four RCTs were multicenter, and studies were performed in a wide range of clinical settings (Table 1). The most common settings were obstetrics (39 RCTs; 9993 participants),^31,32,37,38,39,49,50,53,55,56,63,64,73,74,76,79,81,83,88,91,95,96,97,104,105,111,129,130,131,140,148,152,157,160,167,172,174,177,182^ surgery (27 RCTs; 4223 participants),^54,57,58,61,80,89,99,101,102,103,107,109,111,126,127,128,129,136,138,139,151,154,159,163,178,181,183^ and CKD (22 RCTs; 6013 participants).^33,34,35,36,46,47,68,69,72,77,86,115,119,120,124,146,147,155,159,161,173^ Trials were conducted across all continents, with the most common location being Europe (n = 56).^38,41,45,48,50,52,53,54,55,57,58,60,61,62,63,65,67,78,80,82,84,85,90,91,93,94,96,99,102,103,104,113,115,119,121,127,133,135,136,138,140,141,143,145,150,151,153,154,155,159,163,166,168,172,178,179^ The most common iron preparations evaluated were iron sucrose (n = 68)^31,32,33,34,35,37,38,39,42,48,49,50,51,52,53,55,56,58,60,69,73,75,77,79,80,81,83,88,90,91,92,93,98,101,109,110,112,113,116,117,118,122,123,124,126,127,129,130,131,132,135,141,142,143,146,149,152,155,166,167,171,175,178,179,181,183^ and ferric carboxymaltose (n = 51).^40,41,45,46,47,54,57,61,62,63,64,65,68,70,71,74,78,82,84,85,89,94,103,104,106,107,108,111,114,115,120,137,138,139,144,145,148,151,153,154,157,158,160,163,167,172,174,176,180,182^

### Risk of Bias

The overall risk of bias was low for 31 RCTs^41,45,46,51,54,58,67,70,72,103,104,108,111,113,120,125,126,127,128,129,137,139,145,147,151,159,163,167,170,180^ and high for 106 RCTs. Twenty-two RCTs^31,33,35,36,37,38,39,40,42,43,44,48,49,50,52,53,55,56,59,60,61,62,63,64,65,66,68,69,73,74,75,77,78,79,81,82,84,85,86,87,88,89,90,91,92,94,95,96,97,98,99,100,102,105,109,110,112,114,116,117,118,119,121,122,123,124,125,130,131,132,133,134,136,138,140,142,143,144,146,149,150,152,154,155,156,158,161,162,164,166,169,171,172,173,174,176,177,178,179,180,182,183^ had some concerns for bias. The high risk of bias was most frequently attributable to insufficient blinding of participants, study personnel, or outcome assessors or not providing sufficient information for a decision. A total of 88 included studies^31,32,33,35,36,37,40,42,43,45,46,47,49,50,52,54,56,59,62,63,64,65,66,69,70,71,73,74,75,77,78,82,85,86,87,88,92,94,95,96,97,99,100,101,103,110,111,113,116,117,118,119,120,122,123,124,125,126,131,132,135,137,141,142,147,150,153,155,156,157,158,159,161,162,163,169,170,171,174,179,180,182,184^ were also at high or unclear risk of bias for allocation concealment (eFigure 1 and eTable 5 in the Supplement). Visual inspection of funnel plots for publication bias did not identify any concerns (eFigure 2 in the Supplement).

### Primary Outcome

#### Infection

**Figure 2.**
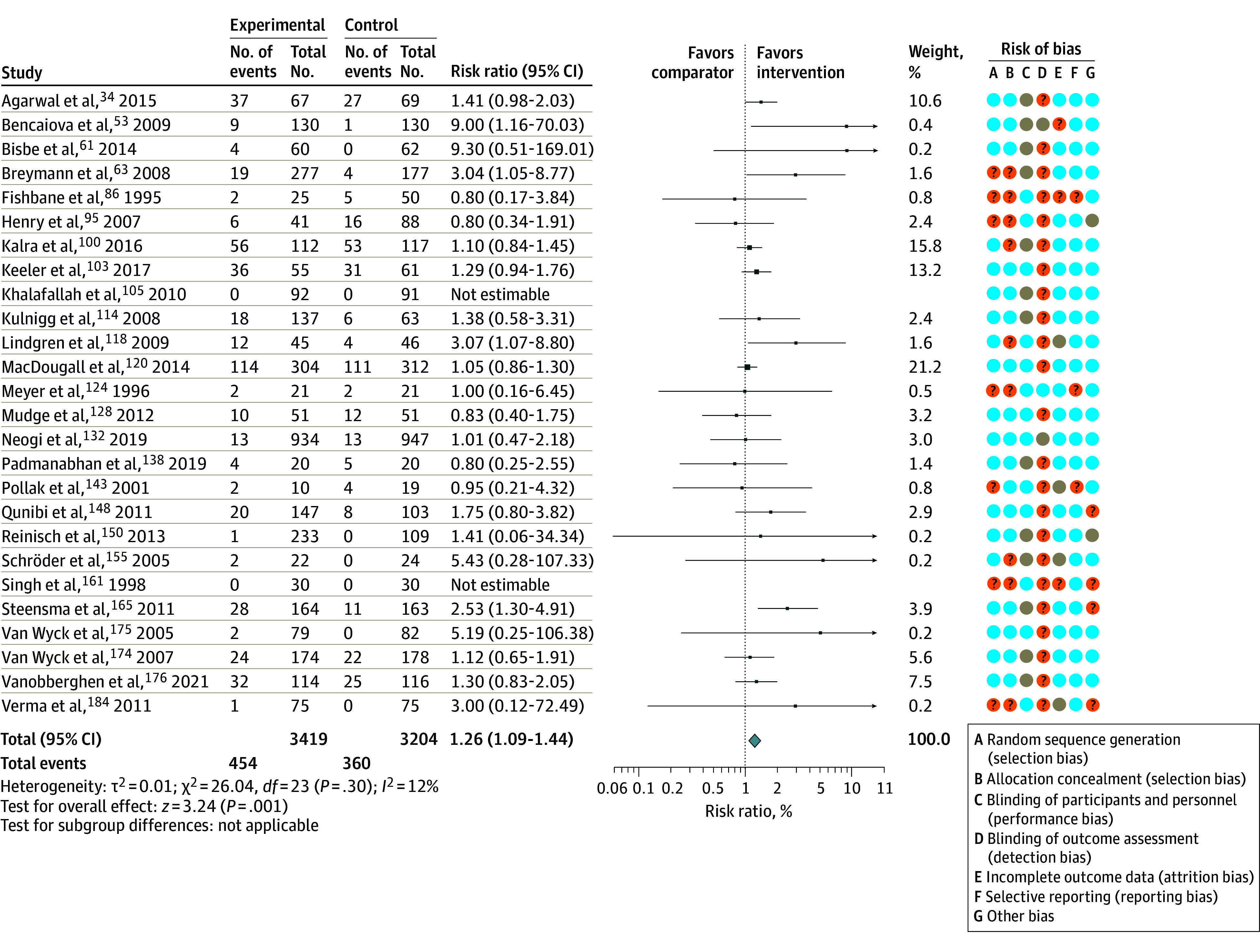
Association Between Risk of Infection and Intravenous Iron When Compared With Oral Iron The risk ratios were calculated using a random-effects model with Mantel-Haenszel weighting. The size of the data markers indicates the weight of the study. Error bars indicate 95% CIs. Orange indicates unclear risk of bias; blue, low risk of bias; and grey, high risk of bias.

**Figure 3.**
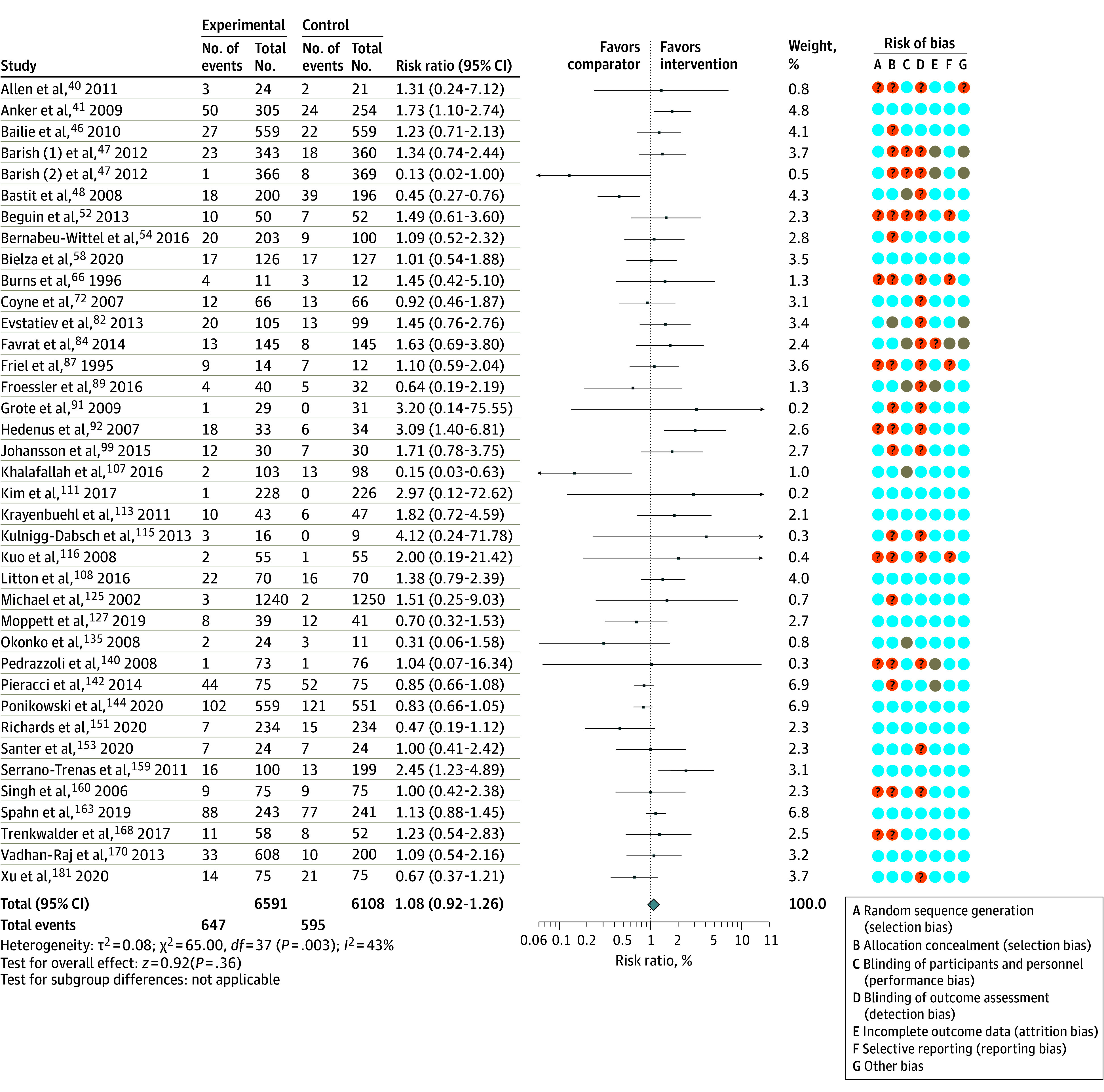
Association Between Risk of Infection and Intravenous Iron When Compared With No Iron The size of the data markers indicates the weight of the study. Error bars indicate 95% CIs. Orange indicates unclear risk of bias; blue, low risk of bias; and grey, high risk of bias.

Sixty-four RCTs^34,40,41,46,47,48,52,53,54,58,61,63,66,72,82,84,86,87,89,92,93,95,99,100,103,105,107,108,111,113,114,115,116,118,120,124,125,127,128,132,135,138,140,142,143,144,148,150,151,153,155,160,161,162,164,165,168,170,174,175,176,181,184^ with 19 322 participants provided sufficient data for analysis of our primary outcome. Intravenous iron was associated with an increased risk of infection when compared with oral iron or no iron (RR, 1.16; 95% CI, 1.03-1.29; *I*^2^ = 36%; *P* = .003) (Table 2, Figure 2, and Figure 3). On the basis of the GRADE framework, this finding was judged to be moderate-quality evidence. In absolute terms, 16 more people per 1000 population (95% CI, 3-29) will experience an infection when intravenous iron is used (eAppendix 3 in the Supplement). In the preplanned sensitivity analysis that excluded studies at high risk of bias, the RR was 1.13 (95% CI, 0.97-1.32; *I*^2^ = 36%; *P* = .08; 16 RCTs; 8561 participants) (eFigure 3 in the Supplement).

**Table 2.** Association Between Intravenous Iron and Primary and Secondary Outcomes

Outcome	No. of studies	No. of participants^a^		Treatment effect	*P* value	*I*^2^, %
		Intravenous iron	Oral iron or no iron			
Primary outcome						
Infection	64	1101/10 010	955/9312	RR (95% CI): 1.16 (1.03 to 1.29)	.003	36
Continuous outcomes						
Hemoglobin	110	10 816	9720	MD (95% CI): 0.57 (0.50 to 0.64) g/dL	<.001	94
RBCs transfused	11	998	956	MD (95% CI): −0.20 (−0.32 to −0.08) cells	<.001	81
Hospital LOS	8	807	883	MD (95% CI): −0.43 (−1.10 to 0.24) d	.05	50
Dichotomous outcomes						
Treatment response^b^	60	4336/7137	2611/6165	RR (95% CI): 1.46 (1.32 to 1.60)	<.001	92
Mortality						
Short term (≤30 d)	15	40/1298	40/1292	RR (95% CI): 0.99 (0.69 to 1.42)	.73	0
Long term (>30 d)	12	165/2752	161/2258	RR (95% CI): 0.94 (0.75 to 1.18)	.63	0
Requirement for RBC transfusion	54	802/6256	989/6040	0.83 (0.76 to 0.89)	<.001	15

Abbreviations: LOS, length of stay; MD, mean difference; RBC, red blood cell; RR, risk ratio.

SI conversion factor: To convert hemoglobin to grams per liter, multiply by 10.

^a^ Number for primary and dichotomous outcomes indicates number of events divided by the total number of participants.

^b^ As defined by the study authors. Examples include increase in hemoglobin by 2 g/dL and proportion of participants without anemia at the end of the study period.

Subgroup analysis by clinical setting found evidence of an increased risk of infection in patients with IBD (RR, 1.73; 95% CI, 1.11-2.71; *I*^2^ = 0%; *P* = .02; 6 RCTs; 908 participants) (eFigure 4 in the Supplement).^82,114,115,118,150,155^ We observed no evidence of an effect associated with iron or anemia profiles at study enrollment (as defined by the study authors) or type of iron preparation used. Both single- and multiple-dose administrations were associated with an increased risk of infection (eFigure 5 in the Supplement). Metaregression found no evidence of an interaction between baseline ferritin level, hemoglobin level, transferrin saturation, study year, and risk of infection (eTable 6 in the Supplement).

Significant variation was found in the definitions and reporting of infection across included studies (eTable 7 in the Supplement). Eight RCTs^41,46,66,103,127,142,144,148^ used an established classification system, with the most common one being the Medical Dictionary for Regulatory Activities.^193^ Two trials used standardized Centers for Disease Control and Prevention definitions for nosocomial infections. Twenty-four RCTs^34,47,54,61,63,82,84,91,93,99,103,111,116,120,125,135,138,140,142,148,159,168,170^ reported the anatomical site of infection, with the lung being the most common site. Seven RCTs^87,107,108,128,132,159,181^ had a pragmatic investigator-defined definition. Forty-nine RCTs^34,40,47,48,52,53,54,61,63,82,84,86,87,92,93,95,99,100,106,111,113,114,115,116,118,120,124,125,128,135,138,140,143,150,151,153,155,159,160,164,165,168,170,174,175,184^ did not provide a definition a priori. There was less information on treatment of infections. One RCT^143^ reported data on antibiotic use between groups. No RCTs reported data on positive microbiology cultures.

### Secondary Outcomes

#### Hemoglobin

Hemoglobin data were available from 111 RCTs^31,32,33,34,35,36,37,38,39,41,42,43,44,45,47,50,51,53,54,55,56,57,60,61,62,63,64,65,66,67,68,69,72,73,74,75,76,77,79,80,81,83,85,89,90,91,92,93,95,97,98,99,101,102,103,104,105,106,107,108,109,110,112,113,115,116,117,118,119,120,123,126,127,130,131,132,133,134,135,136,137,138,139,141,144,146,147,148,149,150,152,153,156,157,158,159,161,164,165,170,171,176,177,183,184^ with 20 776 participants. When pooled, intravenous iron was associated with an increase in hemoglobin at the end of the study period when compared with oral iron or no iron (MD, 0.57 g/dL; 95% CI, 0.50-0.64 g/dL [to convert to grams per liter, multiply by 10]; *I*^2^ = 94%; *P* < .001). Intravenous iron was also associated with a treatment response, as defined by the study authors (eg, increase in hemoglobin of >2 g/dL), when compared with oral iron or no iron (RR, 1.46; 95% CI, 1.32-1.60; *I*^2^ = 92%; *P* < .001) (Table 2; eFigure 6 in the Supplement).

#### RBC Transfusion Requirements

Data on RBC transfusion requirements were available from 54 RCTs^34,38,42,44,45,48,50,52,53,54,57,58,61,63,75,80,88,89,90,93,96,99,101,103,106,107,108,109,110,111,112,120,122,124,126,127,128,129,132,133,134,138,139,140,141,142,151,158,159,163,165,171,179,182^ with 12 116 participants. Intravenous iron was associated with a reduction in the risk of requiring an RBC transfusion when compared with oral iron or no iron (RR, 0.83; 95% CI, 0.76-0.89; *I*^2^ = 15%; *P* < .001) and a lower number of mean RBCs transfused when compared with no iron (MD, −0.20; 95% CI, −0.32 to −0.08; *I*^2^ = 81*%*; *P* < .001) (Table 2; eFigure 7 in the Supplement).

#### Mortality

We observed no evidence of an association of intravenous iron with short-term (RR, 0.99; 95% CI, 0.69-1.42; *I*^2^ = 0%; *P* = .73; 15 RCTs; 3445 participants) or long-term mortality (RR, 0.94; 95% CI, 0.75-1.18; *I*^2^ = 0%; *P* = .63; 12 RCTs; 5010 participants) when compared with oral iron or no iron (Table 2; eFigure 8 in the Supplement).

#### Hospital Length of Stay

There was no evidence of an association of intravenous iron with hospital length of stay when compared with oral iron or no iron (MD, −0.43 days; 95% CI, −1.10 to 0.24 days; *I*^2^ = 50%; *P* = .05; 8 RCTs; 1690 participants) (Table 2; eFigure 8 in the Supplement).

## Discussion

### Key Findings

Our systematic review and meta-analysis identified 154 RCTs^31,32,33,34,35,36,37,38,39,40,41,42,43,44,45,46,47,48,49,50,51,52,53,54,55,56,57,58,59,60,61,62,63,64,65,66,67,68,69,70,71,72,73,74,75,76,77,78,79,80,81,82,83,84,85,86,87,88,89,90,91,92,93,94,95,96,97,98,99,100,101,102,103,104,105,106,107,108,109,110,111,112,113,114,115,116,117,118,119,120,121,122,123,124,125,126,127,128,129,130,131,132,133,134,135,136,137,138,139,140,141,142,143,144,145,146,147,148,149,150,151,152,153,154,155,156,157,158,159,160,161,162,163,164,165,166,167,168,169,170,171,172,173,174,175,176,177,178,179,180,181,182^ across a range of clinical settings. The main findings were (1) intravenous iron was associated with an increased risk of infection (moderate certainty of evidence); (2) there was substantial variation and inconsistency in how infection was defined and reported; and (3) intravenous iron remained associated with improved hemoglobin levels and reduced RBC transfusion requirements. Although we observed no differences in mortality or hospital length of stay, the 95% CIs were wide and could encompass clinically important differences.

Previous systematic reviews^21,22,194^ have summarized the evidence of superior efficacy for intravenous iron over oral iron or no iron but have less rigorously evaluated or reported the risk of infection. Two earlier systematic reviews^21,22^ have reported on the risks of infection but were based on much smaller numbers of RCTs. One review^22^ identified an increased risk of infection (RR, 1.34; 95% CI, 1.10-1.64; 24 RCTs; 4400 participants); the other review^21^ pooled data from 32 RCTs and found a point estimate that favored infection, although the finding was statistically nonsignificant (RR, 1.17; 95% CI, 0.83-1.65).

There is biological plausibility for increased rates of infection in patients receiving iron.^13^ Iron is required for growth by almost all human pathogens.^11,13^ Intravenous iron administration can lead to increased levels of non–transferrin-bound iron, which is associated with impaired T-cell and neutrophil function, worsening organ function in preclinical and clinical studies, and increased pathogen growth.^195,196^ Conversely, preexisting iron deficiency or restriction can impair T-cell, B-cell, and neutralizing antibody responses to infection.^197^ These risks may be further expanded in patients with IBD in whom the prevalence of iron deficiency has been reported to be as high as 90%^20^ and who may also be more susceptible to infection because of the increasing use of immunosuppressive and biological drugs.^198,199^ Although there was no statistical evidence of an increased risk in subgroups other than IBD, the point estimate favored increased infection rates in nearly all groups.

We identified significant variation and inconsistency in the reporting of infection despite the availability of standardized definitions.^200^ Of the 34 ongoing RCTs, 13 have prespecified infection-related outcomes. Of these, 9 RCTs do not have a definition, 2 have an investigator-defined definition, and 2 are using the Clavien-Dindo Classification system (eTable 2 in the Supplement).^201^ Reasons for this poor uptake are unclear but may include limitations with validity, reliability, and practicality. Recent initiatives, such as the updated Sepsis-3 definition^202^ and the Standardised Endpoints in Perioperative Medicine initiative,^203^ have sought to address these limitations and bring consistency to reporting infection. No studies reported data on pathogens or positive microbiology results. This lack of microbiology results has important clinical and mechanistic implications on understanding whether exogenous iron can convert benign bacterial colonization into virulent infection. Common commensal bacteria that are frequently implicated in nosocomial infection, such as *Staphylococcus aureus* and *Staphylococcus epidermidis*, have developed iron acquisition mechanisms and can become pathogenic under suitable conditions.^10,13^

Imbalances of iron homeostasis, including both iron deficiency and overload, can affect the risk of developing infection, but the effect of therapeutic iron on this remains unclear.^204^ The effects are likely to be context specific, depending on the patient’s preexisting iron status, exposure to potential infections and genetic background, and type of iron preparation administerd.^205,206^ We observed no evidence of an interaction between baseline iron status (as measured by ferritin, transferrin saturation, and hemoglobin) and the risk of developing infection, although the number of studies reporting this information in a way that could be analyzed for metaregression ranged from 29 to 40. Trial data from patients with CKD undergoing maintenance hemodialysis^207^ suggest that a high-dose intravenous iron regimen may be safe in patients with ferritin concentrations up to 700 ng/mL (to convert to micrograms per liter, multiply by 1), but data above this threshold are conflicting.^20^ The proportion of low free iron concentrations of the total dose of iron in newer intravenous iron preparations is thought to be associated with a lower risk of infection, which may be one reason why our subgroup analysis by type of preparation demonstrated no superiority of one preparation over another.

### Implications for Practice

Our findings have broad applicability to many patients in whom iron deficiency and anemia are common. Clinical guidelines increasingly advocate the use of intravenous iron, and clinicians and policy makers should recognize that the benefits of treating anemia may need to be balanced against the risk of developing infection. These issues appear more pertinent in clinical settings with high endemic infection burdens where iron supplementation may be neither effective nor safe.^206^

### Implications for Research

This review reinforces the need to quantify all relative risks more precisely alongside the benefits of intravenous iron. Part of the uncertainty around the risk of infection arises from variation and inconsistency in reporting this outcome, despite the availability of standardized definitions. Future trials should be adequately powered and robust, implement standardized definitions for outcomes of infections and focus on hypothesis testing mechanistic studies, such as the interactions between specific pathogens and host iron status on the risk of developing infection.

### Strengths and Limitations

This review has strengths. Its main strength is the strict methodologic process. We followed Cochrane Collaboration, PRISMA, and GRADE recommendations, performed 2 comprehensive searches, and used duplicate data extraction and risk of bias assessments. Compared with the previous reviews,^21,22^ we sought to further characterize the risk of infection and performed clinically relevant subgroup analyses, extracted data on how infection was reported, and included NRSs.

This study also has limitations, which can be attributed to the clinical and methodological differences and variations in infection reporting between the included studies, which may limit the understanding of the nature and true extent of the risk of infection. Updating the previous systematic review^22^ may lead to type 1 error as a result of multiplicity attributable to repeated significance testing. Although we identified an improvement in hemoglobin levels, we did not assess whether this improvement translated into improved health-related quality of life, although this has been investigated by others.^208^ The quality of the included studies was also variable. Nevertheless, a sensitivity analysis including only trials at low risk of bias had minimal effect on the pooled estimate, and the heterogeneity calculation for outcomes of infection and RBC transfusion was not substantial.

## Conclusions

In this systematic review and meta-analysis, intravenous iron administration was associated with an increased risk of infection, which must be considered alongside the potential benefit of treating anemia. Well-designed studies, using standardized definitions of infection, are needed to reduce the uncertainty about this particular risk.

## Notes

Supplement. **eAppendix 1.** Search Strategy **eAppendix 2.** Descriptive Summary of Findings of Included Nonrandomized Studies (NRS) **eAppendix 3.** GRADE Assessment for Primary Outcome **eTable 1.** Characteristics of Included RCTs **eTable 2.** Characteristics of Ongoing RCTs **eTable 3.** Characteristics of Included NRS **eTable 4.** Summary of Risk of Bias Assessment for Nonrandomized Studies **eTable 5.** Risk of Bias Assessments for Individual RCTs **eTable 6.** Meta-regression Analyses **eTable 7.** Summary of Reporting of Infection in Included Studies **eFigure 1.** Risk of Bias Summary for RCTs **eFigure 2.** Funnel Plot for Primary Outcome **eFigure 3.** Sensitivity Analysis for Primary Outcome **eFigure 4.** Subgroup Analysis by Clinical Setting **eFigure 5.** Subgroup Analysis by Type of Preparation, Dosing Schedule and Iron Profile at Enrollment on the Risk of Infection **eFigure 6.** Secondary Outcome—Hemoglobin Forest Plot **eFigure 7.** Secondary Outcomes—Red Blood Cell (RBC) Transfusion Requirements **eFigure 8.** Secondary Outcomes—Mortality and Length of Stay Forest Plots
