# Errors in Data

**DOI:** 10.1001/jamanetworkopen.2021.46637

**Published:** 2022-01-11

In the Original Investigation titled “Risk of Infection Associated With Administration of Intravenous Iron: A Systematic Review and Meta-analysis,”^1^ published November 12, 2021, the number of participants and the number of infection events in each group were incorrectly recorded. Once corrected, the overall point estimate for the risk of infection changed from a relative risk of 1.17 (95% CI, 1.04-1.13, *I*^2^ = 37%) to 1.16 (95% CI, 1.03-1.29, *I*^2^ = 36%). The overall number of participants changed from 32 920 participants to 32 762 participants, and the number of participants providing data for the primary outcome of infection changed from 19 480 participants to 19 322 participants. This article has been corrected.^1^

## References

[1] Shah AA, Donovan K, Seeley C, et al. Risk of infection associated with administration of intravenous iron: a systematic review and meta-analysis. *JAMA Netw Open*. 2021;4(11):e2133935. doi:10.1001/jamanetworkopen.2021.33935 PMC8590171 34767026

