# Error in Data

**DOI:** 10.1001/jamanetworkopen.2024.54479

**Published:** 2024-12-09

In the Original Investigation titled “Risk of Infection Associated With Administration of Intravenous Iron: A Systematic Review and Meta-analysis,” published November 12, 2021,^1^ a data error appeared in the Abstract and main article. Where it was reported that there was a reduction in the risk of a red blood cell transfusion being required compared with oral iron or no iron, the risk ratio should have been 0.83 (95% CI, 0.76-0.89). The article was also corrected on January 11, 2022, to fix errors in the number of participants and infection events and the relative risk of infection.^2^
