# Correction to Meta-Analysis to Acknowledge Retracted Study

**DOI:** 10.1001/jamanetworkopen.2025.0887

**Published:** 2025-01-27

A study that was included in “Risk of Infection Associated With Administration of Intravenous Iron: A Systematic Review and Meta-Analysis,”^1^ published in *JAMA Network Open* on November 12, 2021, was retracted after the meta-analysis was conducted and published. The following notice has been added to the article: “Correction: This meta-analysis was published before 1 of the included studies^76^ was retracted. A reanalysis without that study does not affect the reported results.”
