# Supplementary material for: Risk of Infection Associated With Administration of Intravenous Iron: A Systematic Review and Meta-analysis
Source: JAMA Netw Open. 2021 Nov 12;4(11):e2133935. doi: 10.1001/jamanetworkopen.2021.33935 (PMC8590171; doi:10.1001/jamanetworkopen.2021.33935)
Supplement: Supplement. — eAppendix 1. Search Strategy eAppendix 2. Descriptive Summary of Findings of Included Nonrandomized Studies (NRS) eAppendix 3. GRADE Assessment for Primary Outcome eTable 1. Characteristics of Included RCTs eTable 2. Characteristics of Ongoing RCTs eTable 3. Characteristics of Included NRS eTable 4. Summary of Risk of Bias Assessment for Nonrandomized Studies eTable 5. Risk of Bias Assessments for Individual RCTs eTable 6. Meta-regression Analyses eTable 7. Summary of Reporting of Infection in Included Studies eFigure 1. Risk of Bias Summary for RCTs eFigure 2. Funnel Plot for Primary Outcome eFigure 3. Sensitivity Analysis for Primary Outcome eFigure 4. Subgroup Analysis by Clinical Setting eFigure 5. Subgroup Analysis by Type of Preparation, Dosing Schedule and Iron Profile at Enrollment on the Risk of Infection eFigure 6. Secondary Outcome—Hemoglobin Forest Plot eFigure 7. Secondary Outcomes—Red Blood Cell (RBC) Transfusion Requirements eFigure 8. Secondary Outcomes—Mortality and Length of Stay Forest Plots [file jamanetwopen-e2133935-s001.pdf]

Supplementary Online Content

Shah AA, Donovan K, Seeley C, et al. Risk of infection associated with administration of intravenous iron: a systematic review and meta-analysis. *JAMA Netw Open*. 2021;4(11):e2133935. doi:10.1001/jamanetworkopen.2021.33935

eAppendix 1. Search Strategy

eAppendix 2. Descriptive Summary of Findings of Included Nonrandomized Studies (NRS)

eAppendix 3. GRADE Assessment for Primary Outcome

eTable 1. Characteristics of Included RCTs

eTable 2. Characteristics of Ongoing RCTs

eTable 3. Characteristics of Included NRS

eTable 4. Summary of Risk of Bias Assessment for Nonrandomized Studies

eTable 5. Risk of Bias Assessments for Individual RCTs

eTable 6. Meta-regression Analyses

eTable 7. Summary of Reporting of Infection in Included Studies

eFigure 1. Risk of Bias Summary for RCTs

eFigure 2. Funnel Plot for Primary Outcome

eFigure 3. Sensitivity Analysis for Primary Outcome

eFigure 4. Subgroup Analysis by Clinical Setting

eFigure 5. Subgroup Analysis by Type of Preparation, Dosing Schedule and Iron Profile at Enrollment on the Risk of Infection

eFigure 6. Secondary Outcome—Hemoglobin Forest Plot

eFigure 7. Secondary Outcomes—Red Blood Cell (RBC) Transfusion Requirements

eFigure 8. Secondary Outcomes—Mortality and Length of Stay Forest Plots

This supplementary material has been provided by the authors to give readers additional information about their work.

© 2021 Shah AA et al. *JAMA Network Open.*

eAppendix 1. Search strategy

The following databases were searched for randomised controlled trials (from 2013 to present), systematic reviews and non-randomised trials with a comparator group (from 2007 to present) on 28.9.20:

Cochrane Central Register of Controlled Trials (CENTRAL, *The Cochrane Library*) & CDSR 2020, Issue 9
MEDLINE (OvidSP, 1946 to present)
Embase (OvidSP, 1974 to present)
CINAHL (EBSCOHost) (1937 to present)
PubMed (e-publications ahead of print only)
Transfusion Evidence Library (1950 to present)
LILACS (1982 to present)
Web of Science Conference Proceedings Citation Index-Science (CPCI-S) (Thomson Reuters, 1990 to present)

Ongoing trial databases:
CENTRAL
ClinicalTrials.gov (clinicaltrials.gov)
WHO International Clinical Trials Registry Search Platform (ICTRP)

SEARCH STRATEGIES

CENTRAL (*The Cochrane Library*)
#1 MeSH descriptor: [Ferric Compounds] explode all trees
#2 MeSH descriptor: [Ferrous Compounds] explode all trees
#3 MeSH descriptor: [Iron] explode all trees
#4 (alvofer or colliron or faremio or ferion or feriv or fermed or ferri saccharate or ferric hydroxide sucrose or ferric oxide saccharate or saccharated ferric oxide or ferric saccharate or ferrinemia or ferrisaccharate or ferrivenin or ferrologic or ferrous saccharate or ferrovin or fesin or hemafer s or hemafer-s or idafer or (iron near/2 hydroxide sucrose complex) or iron saccharate or iron sucrose or ironcrose or iviron or nefro-fer or nefrofer or neo ferrum or nephroferol or proferrin or referen or reoxyl or saccharate ferric or saccharate iron or saccharated ferric oxide or saccharated iron oxide or sucro fer or sucrofer or sucroven or veniron or venofer or venotrix)
#5 #1 or #2 or #3 or #4
#6 MeSH descriptor: [Administration, Intravenous] explode all trees
#7 (intravenous* or IV or "IV" or infus* or inject* or parenteral*)
#8 #6 or #7
#9 #5 and #8
#10 (ferric carboxymaltose or Ferinject or Injectafer or Iroprem or ferlecit or ferlixit or ferric gluconate or ferrigluconate or ferrlecit or gluconate ferric sodium or (iron near/2 gluconate) or iron isomaltoside or intravenous iron sucrose or iron sucrose injection* or sodium ferrigluconate or diafer or ferric derisomaltose or venofer or monofer or monafer or monoferro or monover or ferumoxytol or feraheme or rienso or "IV iron" or "IV iron" or "iron therapy" or ((intravenous* or inject* or infus* or parenteral) near/3 iron))
#11 (anaemex or cosmofer or dexferrum or dexiron or dextrafer or dextran fe or dextran ferrous or dextran iron or driken or fenate or fer dextran or ferric dextran or ferridex or tranferrisat or ferrodex or ferrodextran or ferrous dextran or ferrum lek or fervetag or hibiron or imferdex or imferon or impheron or imposil or infed or infufer or iron dextran or ironate or monofar or proferdex or uniferon or uniferon or uniferron)
#12 ferumoxytol or injectafer or infed or cosmofer or iron polymaltose or triferic or ferric pyrophosphate citrate
#13 #4 or #9 or #10 or #11 or #12 with Publication Year from 2013 to 2020, in Trials
#14 #4 or #9 or #10 or #11 or #12 with Cochrane Library publication date Between Jan 2007 and Dec 2020, in Cochrane Reviews, Cochrane Protocols
#15 #13 or #14

MEDLINE (OvidSP)
1. exp Ferric Compounds/
2. exp Ferrous Compounds/
3. exp Iron/
4. (alvofer or colliron or faremio or ferion or feriv or fermed or ferri saccharate or ferric hydroxide sucrose or ferric oxide saccharate or ferric oxide,saccharated or ferric saccharate or ferrinemia or ferrisaccharate or ferrivenin or ferrologic or ferrous saccharate or ferrovin or fesin or hemafer s or hemafer-s or idafer or (iron adj2 hydroxide sucrose complex) or iron saccharate or iron sucrose or ironcrose or iviron or nefro-fer or nefrofer or neo ferrum or nephroferol or proferrin or

© 2021 Shah AA et al. *JAMA Network Open.*

referen or reoxyl or saccharate ferric or saccharate iron or saccharated ferric oxide or saccharated iron oxide or sucro fer or sucrofer or sucroven or veniron or venofer or venotrix).tw,kf.
5. or/1-3
6. exp Administration, Intravenous/
7. (intravenous* or IV or "IV" or infus* or inject* or parenteral*).tw,kf.
8. 6 or 7
9. 5 and 8
10. (ferric carboxymaltose or Ferinject or Injectafer or Iroprem).tw,kf.
11. (ferlecit or ferlixit or ferric gluconate or ferrigluconate or ferrlecit or gluconate ferric sodium or (iron adj2 gluconate) or sodium ferrigluconate or intravenous iron sucrose or iron sucrose injection* or venofer).tw,kf.
12. (diafer or ferric derisomaltose or iron isomaltoside or monofer or monafer or monoferro or monover or ferumoxytol or feraheme or rienso).tw,kf.
13. (IV iron or "IV iron" or iron therapy or ((intravenous* or inject* or infus* or parenteral) adj3 iron)).tw,kf.
14. (ferumoxytol or injectafer or infed or cosmofer or iron polymaltose or triferic or ferric pyrophosphate citrate).mp.

15. (anaemex or cosmofer or dexferrum or dexiron or dextrafer or dextran fe or dextran ferrous or dextran iron or driken or fenate or fer dextran or ferric dextran or ferridex or tranferrisat or ferrodex or ferrodextran or ferrous dextran or ferrum lek or fervetag or hibiron or imferdex or imferon or impheron or imposil or infed or infufer or iron dextran complex or ironate or monofar or proferdex or uniferon or uniferon or uniferron).mp.
16. 4 or 9 or 10 or 11 or 12 or 13 or 14 or 15
17. RANDOMIZED CONTROLLED TRIAL.pt.
18. CONTROLLED CLINICAL TRIAL.pt.
19. (randomi* or trial*).tw,kf.
20. (placebo* or randomly or groups).ab.
21. CLINICAL TRIALS AS TOPIC.sh.
22. or/17-21
23. exp animals/ not humans/
24. 22 not 23
25. 16 and 24
26. limit 25 to yr="2013 -Current"
27. exp COHORT STUDIES/
28. (cohort* or controlled trial* or controlled stud* or comparative trial* or comparative stud* or comparison group* or comparator group* or control group*).tw,kf.
29. ((follow up or observational) adj (study or studies)).tw,kf.
30. (longitudinal* or retrospective* or prospective* or cross sectional*).mp.
31. CROSS-SECTIONAL STUDIES/
32. CONTROLLED BEFORE-AFTER STUDIES/
33. OBSERVATIONAL STUDY/
34. HISTORICALLY CONTROLLED STUDY/
35. INTERRUPTED TIME SERIES ANALYSIS/
36. (nonrandom* or non random*).tw,kf.
37. ((before adj15 (after or during)) or "before-after" or time series or time point* or repeated measur*).tw,kf.
38. (pre-post or pre-test* or pretest* or posttest* or post-test* or (pre adj5 post)).tw,kf.
39. or/27-38
40. Meta-Analysis.pt.
41. Systematic Review.pt.
42. (meta analy$ or metaanaly$).ti.
43. (systematic* adj2 (review* or overview*)).tw,kf.
44. (evidence synthes* or cochrane or medline or pubmed or embase or cinahl or cinhal or lilacs or "web of science" or science citation index or search terms or literature search or published articles or search strateg* or reference list* or bibliograph* or handsearch* or hand search* or manual* search*).ab.
45. (additional adj (papers or articles or sources)).ab.
46. ((electronic or online) adj (sources or resources or databases)).ab.
47. (relevant adj (journals or articles)).ab.
48. "REVIEW LITERATURE AS TOPIC"/
49. "Systematic Reviews as Topic"/
50. META-ANALYSIS AS TOPIC/
51. or/40-50
52. Review.pt.
53. exp CLINICAL TRIALS AS TOPIC/

54. selection criteria.ab. or critical appraisal.ti.
55. (data adj (extraction or analys*)).ab.
56. RANDOMIZED CONTROLLED TRIALS/
57. OBSERVATIONAL STUDY/
58. ((cohort* or observational or retrospective*) adj1 (trial* or stud*)).tw,kf.
59. or/53-58
60. 52 and 59
61. 51 or 60
62. (Comment or Letter or Editorial).pt.
63. 61 not 62
64. 39 or 63
65. exp animals/ not humans/
66. 64 not 65
67. 16 and 66
68. limit 67 to yr="2007 -Current"
69. 26 or 68

EMBASE (OvidSP)
1. ferric carboxymaltose/ or ferric gluconate/ or iron dextran/ or iron isomaltose/ or iron saccharate/
2. (alvofer or colliron or faremio or ferion or feriv or fermed or ferri saccharate or ferric hydroxide sucrose or ferric oxide saccharate or ferric oxide,saccharated or ferric saccharate or ferrinemia or ferrisaccharate or ferrivenin or ferrologic or ferrous saccharate or ferrovin or fesin or hemafer s or hemafer-s or idafer or (iron adj2 hydroxide sucrose complex) or iron saccharate or iron sucrose or ironcrose or iviron or nefro-fer or nefrofer or neo ferrum or nephroferol or proferrin or referen or reoxyl or saccharate ferric or saccharate iron or saccharated ferric oxide or saccharated iron oxide or sucro fer or sucrofer or sucroven or veniron or venofer or venotrix).tw.
3. (anaemex or cosmofer or dexferrum or dexiron or dextrafer or dextran fe or dextran ferrous or dextran iron or driken or fenate or fer dextran or ferric dextran or ferridex or tranferrisat or ferrodex or ferrodextran or ferrous dextran or ferrum lek or fervetag or hibiron or imferdex or imferon or impheron or imposil or infed or infufer or iron dextran complex or ironate or monofar or proferdex or uniferon or uniferon or uniferron).tw.
4. (Ferric carboxymaltose or Ferinject or Injectafer or Iroprem).tw.
5. (ferlecit or ferlixit or ferric gluconate or ferrigluconate or ferrlecit or gluconate ferric sodium or (iron adj2 gluconate) or sodium ferrigluconate or intravenous iron sucrose or iron sucrose injection* or venofer).tw,kf.
6. (diafer or ferric derisomaltose or iron isomaltoside or monofer or monafer or monoferro or monover or ferumoxytol or feraheme or rienso).tw,kf.
7. ferumoxytol/
8. (IV iron or "IV iron" or iron therapy or ((intravenous* or inject* or infus* or parenteral) adj3 iron)).tw.
9. (ferumoxytol or injectafer or infed or cosmofer or iron polymaltose or triferic or ferric pyrophosphate citrate or feraheme or rienso).mp.
10. 1 or 2 or 3 or 4 or 5 or 6 or 7 or 8 or 9
11. Meta Analysis/
12. Systematic Review/
13. (meta analy* or metaanalys*).tw.
14. ((systematic* or literature) adj2 (review* or overview* or search*)).tw.
15. (cochrane or embase or cinahl or cinhal or lilacs or BIDS or science citation index or psyclit or psychlit or psycinfo or psychinfo or cancerlit).ti,ab.
16. ((electronic* or online) adj (sources or resources or databases)).ab.
17. (additional adj (articles or papers or sources)).ab.
18. (reference lists or bibliograph* or handsearch* or hand search* or manual* search*).ab.
19. (relevant adj (journals or articles)).ab.
20. (search term* or published articles or search strateg*).ab.
21. or/11-20
22. (data extraction or selection criteria or inclusion criteria).ab.
23. review.pt.
24. 21 or (22 and 23)
25. editorial.pt.
26. 24 not 25
27. crossover-procedure/ or double-blind procedure/ or randomized controlled trial/ or single-blind procedure/
28. (random* or factorial* or crossover* or cross over* or cross-over* or placebo* or doubl* blind* or singl* blind* or assign* or allocat* or volunteer*).mp.
29. 27 or 28

30. MAJOR CLINICAL STUDY/
31. LONGITUDINAL STUDY/
32. RETROSPECTIVE STUDY/
33. OBSERVATIONAL STUDY/
34. INTERVENTION STUDY/
35. PROSPECTIVE STUDY/ not RANDOMIZED CONTROLLED TRIAL/
36. COHORT ANALYSIS/
37. COMPARATIVE STUDY/
38. (cohort* or controlled trial* or controlled stud* or comparative trial* or comparative stud* or comparison group* or comparator group* or control group*).tw.
39. ((follow up or observational) adj (study or studies)).tw.
40. (longitudinal* or retrospective* or prospective* or cross sectional*).mp.
41. (nonrandom* or non random*).tw.
42. ((before adj15 (after or during)) or "before-after" or time series or time point* or repeated measur*).tw.
43. (pre-post or pre-test* or pretest* or posttest* or post-test* or (pre adj5 post)).tw.
44. or/30-43
45. 26 or 44
46. 10 and 29
47. limit 46 to yr="2013 -Current"
48. 10 and 45
49. limit 48 to yr="2007 -Current"
50. 47 or 49

CINAHL (EBSCOHoST)

S1	(MH "Ferric Compounds+")
S2	(MH "Ferrous Compounds")
S3	(MH "Iron")
S4	S1 OR S2 OR S3
S5	(MH "Administration, Intravenous+")
S6	TX (intravenous* or IV or "IV" or infus* or inject* or parenteral)
S7	S5 OR S6
S8	S4 AND S7
S9	TX (alvofer or colliron or faremio or ferion or feriv or fermed or ferri saccharate or ferric hydroxide sucrose or ferric oxide saccharate or ferric oxide,saccharated or ferric saccharate or ferrinemia or ferrisaccharate or ferrivenin or ferrologic or ferrous saccharate or ferrovin or fesin or hemafer s or hemafer-s or idafer or (iron N2 hydroxide sucrose complex) or iron saccharate or iron sucrose or ironcrose or iviron or nefro-fer or nefrofer or neo ferrum or nephroferol or proferrin or referen or reoxyl or saccharate ferric or saccharate iron or saccharated ferric oxide or saccharated iron oxide or sucro fer or sucrofer or sucroven or veniron or venotrix)
S10	TX (anaemex or cosmofer or dexferrum or dexiron or dextrafer or dextran fe or dextran ferrous or dextran iron or driken or fenate or fer dextran or ferric dextran or ferridex or tranferrisat or ferrodex or ferrodextran or ferrous dextran or ferrum lek or fervetag or hibiron or imferdex or imferon or impheron or imposil or infed or infufer or iron dextran complex or ironate or monofar or proferdex or uniferon or uniferon or uniferron)
S11	TX (ferric carboxymaltose or Ferinject or Injectafer or Iroprem or venofer or ferlecit or ferlixit or ferric gluconate or ferrigluconate or ferrlecit or gluconate ferric sodium or (iron N2 gluconate) or sodium ferrigluconate or diafer or ferric derisomaltose or iron isomaltoside or monofer or monafer or monoferro or monover or ferumoxytol or feraheme or rienso or "IV iron" or "IV iron" or "iron therapy" or ((intravenous* or inject* or infus* or parenteral) N3 iron))
S12	S8 OR S9 OR S10 OR S11
S13	(MH "Prospective Studies+")
S14	(MH "Case Control Studies+")
S15	(MH "Correlational Studies") OR (MH "Cross Sectional Studies")
S16	TI ((cohort study or cohort studies)) OR AB ((cohort study or cohort studies))
S17	TI ((observational stud* or retrospective stud*)) OR AB ((observational stud* or retrospective stud*))
S18	S13 OR S14 OR S15 OR S16 OR S17
S19	(MH Clinical Trials+)
S20	PT Clinical Trial
S21	TI ((controlled trial*) or (clinical trial*)) OR AB ((controlled trial*) or (clinical trial*))

S22 TI ((singl* blind*) OR (doubl* blind*) OR (trebl* blind*) OR (tripl* blind*) OR (singl* mask*) OR (doubl* mask*) OR (tripl* mask*)) OR AB ((singl* blind*) OR (doubl* blind*) OR (trebl* blind*) OR (tripl* blind*) OR (singl* mask*) OR (doubl* mask*) OR (tripl* mask*))

S23 TI randomi* OR AB randomi*
S24 MH RANDOM ASSIGNMENT
S25 TI ((phase three) or (phase III) or (phase three)) or AB ((phase three) or (phase III) or (phase three))
S26 (TI (random* N2 (assign* or allocat*))) OR (AB (random* N2 (assign* or allocat*)))
S27 MH PLACEBOS OR (TI placebo* OR AB placebo*)
S28 S19 OR S20 OR S21 OR S22 OR S23 OR S24 OR S25 OR S26 OR S27
S29 MH META ANALYSIS
S30 MH SYSTEMATIC REVIEW
S31 TI ("meta analys*" OR metaanalys* OR "systematic review" OR "systematic overview" OR "systematic search*") OR AB ("meta analys*" OR metaanalys* OR "systematic review" OR "systematic overview" OR "systematic search*")
S32 TI ("literature review" OR "literature overview" OR "literature search*") OR AB ("literature review" OR "literature overview" OR "literature search*")
S33 TI (cochrane OR embase OR cinahl OR cinhal OR lilacs OR BIDS OR science AND citation AND index OR cancerlit) OR AB (cochrane OR embase OR cinahl OR cinhal OR lilacs OR BIDS OR science AND citation AND index OR cancerlit)
S34 MH QUANTITATIVE STUDIES
S35 (MH "Controlled Before-After Studies") OR (MH "Historically Controlled Study") OR (MH "Interrupted Time Series Analysis") OR (MH "Nonrandomized Trials") OR (MH "Pretest-Posttest Design+") OR (MH "Static Group Comparison") OR (MH "Solomon Four-Group Design")
S36 TX nonrandom* or non random*
S37 TX ((before N15 (after or during)) or "before-after" or time series or time point* or repeated measur*)
S38 TX (pre-post or pre-test* or pretest* or posttest* or post-test* or (pre N5 post))
S39 TX TI ((controlled stud*) or (clinical stud*)) OR AB ((controlled stud*) or (clinical stud*))
S40 S18 OR S29 OR S30 OR S31 OR S32 OR S33 OR S34 OR S35 OR S36 OR S37 OR S38 OR S39
S41 S12 AND S28
S42 S12 AND S28 Limiters - Published Date: 20130101-20201231
S43 S12 AND S40
S44 S12 AND S40 Limiters - Published Date: 20070101-20201231
S45 S42 OR S44

PubMed

#1 (alvofer[TIAB] OR colliron[TIAB] OR faremio[TIAB] OR ferion[TIAB] OR feriv[TIAB] OR fermed[TIAB] OR "ferri saccharate"[TIAB] OR "ferric hydroxide sucrose"[TIAB] OR "ferric oxide saccharate" [TIAB] OR "ferric saccharate"[TIAB] OR ferrinemia[TIAB] OR ferrisaccharate[TIAB] OR ferrivenin[TIAB] OR ferrologic[TIAB] OR "ferrous saccharate"[TIAB] OR ferrovin[TIAB] OR fesin[TIAB] OR "hemafer-s"[TIAB] OR "hemafer-s"[TIAB] OR idafer[TIAB] OR "iron hydroxide sucrose complex"[TIAB] OR "iron saccharate"[TIAB] OR "iron sucrose"[TIAB] OR ironcrose[TIAB] OR iviron[TIAB] OR "nefro-fer"[TIAB] OR nefrofer[TIAB] OR "neo ferrum"[TIAB] OR nephroferol[TIAB] OR proferrin[TIAB] OR referen[TIAB] OR reoxyl[TIAB] OR "saccharate ferric"[TIAB] OR "saccharate iron"[TIAB] OR "saccharated ferric oxide"[TIAB] OR "saccharated iron oxide"[TIAB] OR "sucro fer"[TIAB] OR sucrofer[TIAB] OR sucroven[TIAB] OR veniron[TIAB] OR venofer[TIAB] OR venotrix[TIAB] OR anaemex[TIAB] OR cosmofer[TIAB] OR dexferrum[TIAB] OR dexiron[TIAB] OR dextrafer[TIAB] OR "dextran fe"[TIAB] OR "dextran ferrous"[TIAB] OR "dextran iron"[TIAB] OR driken[TIAB] OR fenate[TIAB] OR "fer dextran"[TIAB] OR "ferric dextran"[TIAB] OR ferridex[TIAB] OR tranferrisat[TIAB] OR ferrodex[TIAB] OR ferrodextran[TIAB] OR "ferrous dextran"[TIAB] OR "ferrum lek"[TIAB] OR fervetag[TIAB] OR hibiron[TIAB] OR imferdex[TIAB] OR imferon[TIAB] OR impheron[TIAB] OR imposil[TIAB] OR infed[TIAB] OR infufer[TIAB] OR "iron dextran"[TIAB] OR ironate[TIAB] OR monofar[TIAB] OR proferdex[TIAB])
#2 (intravenous*[TIAB] OR IV[TIAB] OR "IV"[TIAB] OR infus*[TIAB] OR inject*[TIAB] OR parenteral*[TIAB])
#3 #1 AND #2
#4 ("ferric carboxymaltose"[TIAB] OR Ferinject[TIAB] OR Injectafer[TIAB] OR Iroprem[TIAB] OR venofer[TIAB] OR ferlecit[TIAB] OR ferlixit[TIAB] OR "ferric gluconate"[TIAB] OR ferrigluconate[TIAB] OR ferrlecit[TIAB] OR "gluconate ferric sodium"[TIAB] OR " iron gluconate"[TIAB] OR "sodium ferrigluconate"[TIAB] OR diafer[TIAB] OR "ferric derisomaltose"[TIAB] OR "iron isomaltoside"[TIAB] OR monofer[TIAB] OR monafer[TIAB] OR monoferro[TIAB] OR monover[TIAB] OR ferumoxytol[TIAB] OR feraheme[TIAB] OR rienso[TIAB] OR "IV iron"[TIAB] OR "IV iron"[TIAB] OR "intravenous iron"[TIAB] OR "iron injection"[TIAB] OR "injectable iron"[TIAB] OR "iron infusion"[TIAB] OR "iron infusions"[TIAB] OR "infused iron"[TIAB] OR "parenteral iron"[TIAB] OR "iron therapy"[TIAB])
#5 #3 OR #4

© 2021 Shah AA et al. *JAMA Network Open.*

#6 (random* OR blind* OR "control group" OR placebo* OR controlled OR groups OR trial* OR "systematic review" OR "meta-analysis" OR metaanalysis OR "literature search" OR medline OR pubmed OR cochrane OR embase OR case control* OR "case series" OR cohort* OR comparative OR comparison OR comparator OR "follow-up study" OR "follow-up studies" OR observational* OR retrospective* or non-random* OR pre-post or pre-test* or pretest* or posttest* or post-test* OR before-after) AND (publisher[sb] OR inprocess[sb] OR pubmednotmedline[sb])
#7 #5 AND #6

TRANSFUSION EVIDENCE LIBRARY
Subject Area: Clinical Practice – Management of Anaemia
Date Limit: 2013-2020

LILACS
tw:((ferric carboxymaltose OR ferinject OR injectafer OR iroprem OR iron sucrose OR venofer OR ferlecit OR ferlixit OR ferric gluconate OR ferrigluconate OR ferrlecit OR gluconate ferric sodium OR iron gluconate OR sodium ferrigluconate OR diafer OR ferric derisomaltose OR iron isomaltoside OR monofer OR monafer OR monoferro OR monover OR ferumoxytol OR feraheme OR rienso OR iv iron OR IV iron OR iron therapy OR intravenous iron OR injectable iron OR iron infusion OR iron infusions OR infused iron OR parenteral iron)) AND (instance:"regional") AND (db:("LILACS") AND year_cluster:("2007"-"2020"))

WEB OF SCIENCE
#1 TS=(alvofer or colliron or faremio or ferion or feriv or fermed or ferri saccharate or ferric hydroxide sucrose or ferric oxide saccharate or ferric saccharate or ferrinemia or ferrisaccharate or ferrivenin or ferrologic or ferrous saccharate or ferrovin or fesin or hemafer s or hemafer-s or idafer or iron hydroxide sucrose or iron saccharate or iron sucrose or ironcrose or iviron or nefro-fer or nefrofer or neo ferrum or nephroferol or proferrin or referen or reoxyl or saccharate ferric or saccharate iron or saccharated ferric oxide or saccharated iron oxide or sucro fer or sucrofer or sucroven or veniron or venofer or venotrix)
#2 TS=(anaemex or cosmofer or dexferrum or dexiron or dextrafer or dextran fe or dextran ferrous or dextran iron or driken or fenate or fer dextran or ferric dextran or ferridex or tranferrisat or ferrodex or ferrodextran or ferrous dextran or ferrum lek or fervetag or hibiron or imferdex or imferon or impheron or imposil or infed or infufer or iron dextran or ironate or monofar or proferdex)
#3 #1 or #2
#4 TS=(intravenous* or IV or "IV" or infus* or inject* or parenteral*)
#5 #3 and #4
#6 TS=(ferric carboxymaltose or Ferinject or Injectafer or Iroprem or venofer or ferlecit or ferlixit or ferric gluconate or ferrigluconate or ferrlecit or gluconate ferric sodium or (iron near/2 gluconate) or sodium ferrigluconate or diafer or ferric derisomaltose or iron isomaltoside or monofer or monafer or monoferro or monover or ferumoxytol or feraheme or rienso or "IV iron" or "IV iron" or "iron therapy" or ((intravenous* or inject* or infus* or parenteral) NEAR/3 iron))
#7 #5 OR #6
#8 TS=(random* OR blind* OR "control group" OR placebo* OR controlled OR groups OR trial* OR "systematic review" OR "meta-analysis" OR metaanalysis OR "literature search" OR medline OR pubmed OR cochrane OR embase OR case control* OR "case series" OR cohort* OR comparative OR comparison OR comparator OR "follow-up study" OR "follow-up studies" OR observational* OR retrospective* or non-random* OR pre-post or pre-test* or pretest* or posttest* or post-test* OR before-after)
#9 #7 AND #8 [Indexes=CPCI-S, BKCI-S Timespan=2007-2020]

ClinicalTrials.gov
Intervention or Title: (ferric carboxymaltose OR ferinject OR injectafer OR iroprem OR iron sucrose OR venofer OR iron isomaltoside OR monofer OR ferumoxytol OR feraheme OR ferrlecit or rienso OR IV iron OR iron th
erapy OR intravenous iron OR iron infusion)

ICTRP
Single Search Box: (ferric carboxymaltose OR ferinject OR injectafer OR iroprem OR iron sucrose OR venofer OR iron isomaltoside OR monofer OR ferumoxytol OR feraheme OR ferrlecit or rienso OR IV iron OR iron therapy OR intravenous iron OR iron infusion

© 2021 Shah AA et al. *JAMA Network Open.*

eAppendix 2. Descriptive summary of findings of non-randomized studies (NRS)

The number of participants in the included NRS ranged from 39 to 2547. The clinical settings included surgery (n = 5), CKD (n = 3) and digestive diseases.

Risk of bias
Overall risk of bias was moderate for seven NRS and only one study was deemed to be at overall low risk of bias. All studies were at serious or moderate risk of bias due to confounding, mainly due to the absence of or lack of controlling for important confounders. Risk of bias in measurement of outcomes was moderate across all studies due to outcome assessors either being aware of the intervention status or insufficient information was available to make a decision.

Infection
Three studies reported no statistically significant increases in the risk of infection associated with intravenous iron. One study found an increased risk and two studies reported a lowering of risk. One study, which lacked a comparator group, only reported an incidence of 25.9% of infection in patients with underlying digestive diseases receiving intravenous iron. Four studies provided no definition of infection. Of the remaining four studies, three studies used recognised classification systems (CDC, MedDRA, ICD-9 classification) and one study used an investigator-led definition.

© 2021 Shah AA et al. *JAMA Network Open.*

eAppendix 3. GRADE assessment for primary outcome

Certainty assessment							Summary of findings				
Participants (studies) Follow up	Risk of bias	Inconsistency	Indirectness	Imprecision	Publication bias	Overall certainty of evidence	Study event rates (%)		Relative effect (95% CI)	Anticipated absolute effects	
							With comparator	With IV iron		Risk with comparator	Risk difference with IV iron
**Infection**											
19480 (64 RCTs)	not serious [a]	serious [b]	not serious [c]	not serious [d]	none	⨁⨁⨁◯ MODERATE	950/9391 (10.1%)	1102/10089 (10.9%)	**RR 1.17** (1.04 to 1.31)	100 per 1,000	**17 more per 1,000** (from 4 more to 31 more)

CI: Confidence interval; **IV:** Intravenous; **RR:** Risk ratio

Explanations

a. Most information is from studies at low or unclear risk of bias. Point estimate largely unchanged in sensitivity analyses that excludes studies at high risk of bias.
b. Downgraded for inconsistency due to moderate heterogeneity (41.4%) and variation in point estimates across studies.
c. Population, intervention and comparators are broadly representative of current practice. Iron sucrose continues to be used in low to middle-income countries.
d. Large number of participants and 95% CI excludes no effect

© 2021 Shah AA et al. *JAMA Network Open.*

eTable 1. Characteristics of included randomized controlled trials

Study ID	Methods	Participants	Interventions (IV iron)	Comparator	Primary outcome	Secondary outcomes
Abdelazim et al,[31] 2017	Multicenter RCT (n = 3) (Kuwait)	Pregnant women >18 years, 24-30 weeks' gestation, Hb 8-10 g/dL due to iron deficiency anaemia<br><br>n = 260	Iron sucrose 200mg doses - calculated to total iron deficit	Oral iron	Treatment efficacy - changes in hemoglobin, serum ferritin, MCV at 3-months post-treatment	-
Abhilashini et al,[32] 2014	Single center RCT (India)	Pregnant women, 30-34 weeks' gestation, with established iron deficiency anaemia with Hb 6-8 g/dL<br><br>n = 100	Iron sucrose according to Ganzoni formula, given over multiple dose	Ferrous sulphate 200mg x3/day	Change in Hb up to 37 weeks' gestation	
Adhikary et al,[33] 2011	Single center RCT (Nepal)	CKD established on EPO, Hb <11 g/dL, Tsat <25%, Ferritin <300 ng/mL<br><br>n = 90	Iron sucrose (Total 1000mg over 5 weeks)	Ferrous fumarate 152mg x3/day<br>Both groups: 4000 IU EPO weekly	Improvement in Hb 30 days after therapy	
Agarwal et al,[35] 2006	Multicenter RCT (n = 26) (USA)	Age >18 years, CKD (eGFR between 10 and 59 ml/min), Hb <12 g/dL, serum ferritin <100 ng/mL, Tsat <20%<br><br>n = 75	Ferric gluconate 250mg on 4 separate days	Ferrous sulfate 325mg x3/day for 6 weeks	Change in hemoglobin from baseline to end point (day 43)	(i) Changes in iron parameters; (ii) HRQoL
Agarwal et al,[34] 2015	Multicenter RCT (n = 2) (USA)	CKD Stage 3, Hb <120 g/L, Serum ferritin <100 ng/mL or Tsat <25%<br><br>n = 136	Iron sucrose 200mg weekly for 5 weeks	Ferrous sulfate 325mg x3/day for 8 weeks	Between group difference in slope of mGFR from baseline to two years	(i) Changes in hemoglobin; (ii) Changes in KDQOL.
Aggarwal et al,[36] 2003	Single center RCT (India)	Established CKD on conservative treatment, age >15 years, Hb 5-8 g/dL	Iron dextran 200 mg	Oral iron sulphate 200mg x3/day	Not specified, but measurements included changes in Hb and iron	

© 2021 Shah AA et al. *JAMA Network Open.*

		n = 40			profiles, and adverse events	
Akhtar et al,[37] 2018	Single center RCT (Pakistan)	Postpartum anaemia within 24-48 hours of delivery, Hb 7-10 g/dL and ferritin <15 mcg/L<br><br>n = 60	Iron sucrose 400mg (two doses)	Ferrous sulphate 200mg x3/day for 6 weeks	Changes in Hb and ferritin from baseline until 40 days postpartum	
Al et al,[38] 2005	Single center RCT (Turkey)	Pregnant women between 26-34 weeks' gestation, Hb 8 to 10.5 g/dL, ferritin <13 ng/mL<br><br>n = 90	Iron sucrose calculated to target hemoglobin (divided doses)	Oral iron 100mg x3/day	Hb concentration at day 28 post-treatment and at delivery	(i) Changes in ferritin levels; (ii) adverse effects; and (iii) fetal birth weight
Al-Momen et al,[39] 2006	Single center RCT (Saudi Arabia)	Pregnant women gestational age <32 weeks, Hb <9 g/dL, ferritin <20 ng/mL<br><br>n = 111	Iron sucrose calculated to target Hb	Ferrous sulphate 300 mg x3/day	Not specified, but measurements included changes in Hb and iron profiles, and safety monitoring	
Allen et al,[40] 2015	Multicenter RCT (n = 15) (USA)	Age >18 years, Restless leg syndrome with baseline IRLS score >15<br><br>n = 46	Ferric carboxymaltose 500mg given twice	0.9% saline IV placebo	Changes in IRLS scores	(i) Changes in CG1-1 and PG-1; (ii) safety
Anker et al,[41] 2009	Multicenter RCT (n = 75) (Europe)	Adults >18 years, NYHA class II or III, LVEF <45%, Hb 95 - 135 g/dL, ferritin <100 ng/mL or 100 - 299 ng/mL with Tsat <20%<br><br>n = 459	Ferric carboxymaltose 200mg doses calculated to iron deficit	0.9% saline IV placebo (opaque infusion kits)	Self-reported Patient Global Assessment and NYHA functional class at week 24	(i) self-reported PGA and NYHA class at weeks 4 and 12; (ii) distance on 6-minute walk-test; (iii) EQ-5D-5L; (iv) adverse events; (v) changes in Hb and iron profiles
Athibovonsuk et al,[42] 2013	Single center (Thailand)	Age 20-70 years, Gynaecological malignancy receiving first-line platinum-based chemotherapy<br><br>n = 64	Iron sucrose 200mg after each cycle of chemotherapy	Ferrous fumarate 200mg x3/day throughout	Requirement for blood transfusion	(i) total number of transfusion units; (ii) number of cycles requiring blood transfusion; (iii) changes in

© 2021 Shah AA et al. *JAMA Network Open.*

				course of chemotherapy		hemoglobin; (iv) serious adverse events
Auerbach et al,[43] 2004	Multicenter RCT (n = 4) (USA)	Histologic diagnosis of cancer, Hb <10.5 g/dL, ferritin <450 ng/mL, Tsat <19%<br><br>n = 157	Intervention 1: Iron dextran 100mg boluses<br><br>Intervention 2: Iron dextran total dose infusion	Comparator 1: No iron<br><br>Comparator 2: Ferrous sulphate 325mg daily	Change in Hb from baseline to end point (6 weeks)	Haematopoietic response; (ii) time to Hb response; (iii) QoL
Auerbach et al,[44] 2010	Multicenter RCT (Europe, USA, Russia)	Age >18 years, active nonmyeloid malignancies, Hb <10 g/dL related to cancer and chemotherapy<br><br>n = 243	Iron dextran 400mg	No iron<br><br>All groups received darbopoeitin 300-500 mcg	Achievement of target Hb (>11 g/dL)	(i) Time to acheive target Hb; (ii) proportion of patients with an Hb rise >2 g/dL; (iii) change in FACT-F fatigue scale; (iv) safety
Bager et al,[45] 2014	Single center RCT (Denmark)	Age >18 years admitted with non-variceal upper GI bleeding; ~48h post stabilisation of bleeding source and endoscopy; Anaemia according WHO definitions (Hb <12 g/dL in women; <13 g/dL in men)<br><br>n = 97	Ferric carboxymaltose 1000mg	Oral ferrous sulphate 100mg x2/day for 3 months	Difference in Hb between both groups at 3 months post-treatment	(i) Proportion of patients with normal Hb at end of study; (ii) proportion of patients with Hb increased >2 g/dL; (iii) restoration of iron stores
Bailie et al,[46] 2010	Multicenter RCT (n=62) (USA), cross-over trial	Adults with anaemia (WHO definition) with evidence of iron deficiency into one of the following cohorts:<br><br>(i) secondary to CKD (Tsat <25% and ferritin <300 ng/mL)<br>(ii) secondary to IBD (Tsat <25% and ferritin <300 ng/mL)	Ferric carboxymaltose 15mg/kg	0.9% saline IV placebo	Treatment-related adverse events within first 7 days	(i) Treatment-related adverse events during first 24 hours; (ii) all adverse events

© 2021 Shah AA et al. *JAMA Network Open.*

		(iii) other conditions (Tsat <25% and ferritin <100 ng/mL)<br><br>n = 559				
Barish et al,[47] 2012 (1)*	Multicenter RCT (n = 95) (USA)	Age 18-85 years old, Diagnosis of iron deficiency anaemia (Hb <11g/dL), Ferritin <100 ng/mL, Tsat <30% (mixed population included CKD, Postpartum, GI bleeding, other)<br><br>n = 708	Intravenous ferric carboxymaltose 750mg weekly until calculated iron deficit met (Ganzoni formula)	Standard medical care	Evaluate safety of maximal dose of ferric carboxymaltose	Changes in hemoglobin and iron status
Barish et al,[47] 2012 (2)*	Multicenter RCT (n = 95) (USA)	Age 18-85 years, Diagnosis of iron deficiency anaemia (Hb <11g/dL), Ferritin <100 ng/ml, Tsat <30% (mixed population included CKD, Postpartum, GI bleeding, other)<br><br>n = 738	Intravenous ferric carboxymaltose 750mg or 15 mg/kg, whichever was smaller, one-off dosing	Standard medical care	Evaluate safety of maximal dose of ferric carboxymaltose	Changes in hemoglobin and iron status
Bastit et al,[48] 2008	Multicentre RCT (Europe)	Age >18 years, Hb <11 g/dL, non-myeloid malignancy<br><br>n = 396	Iron sucrose 200mg at each study visit (weeks 1, 4, 7, 10, 13 and 16)	Usual care	Proportion of patients achieving Hb >12 g/dL or rise of >2 g/dL	(i)time to achieving response; (ii) transfusion requirements; (iii) HRQoL measured by FACT-F; (iv) adverse events
Batool et al,[49] 2018	Single center RCT (Pakistan)	Postpartum anaemia (Hb <10 g/dL)<br><br>n = 82	Iron sucrose 200mg weekly for six weeks	Ferrous sulphate 200mg x3/day for 6 weeks	Hb response, defined as Hb rise of >3.5 g/dL, after 6 weeks of therapy	
Bayoumeu et al,[50] 2002	Single center RCT (France)	Age >18 years, Pregnant women at 6 months gestation, Hb 8 - 10 g/dL, ferritin <50 mcg/L<br><br>n = 50	Iron sucrose calculated to target Hb given	Oral iron 80 md x3/day for 4 weeks	Primary outcome not specified, but outcome measures included changes in Hb, iron	

© 2021 Shah AA et al. *JAMA Network Open.*

			over 6 slow IV injections		profiles up to day 30, neonatal iron status	
Beck-da-Silva et al,[51] 2013	Multicenter RCT (Brazil)	Age <18 years, Heart failure NYHA functional class II, LVEF <40% within previous six months, Hb 9-12 g/dL, Tsat <20%, ferritin <500 mcg/L<br><br>n = 23	Iron sucrose 200mg once a week for 5 weeks + oral placebo t.d.s. for 8 weeks	Comparator 1: Ferrous sulphate 200mg t.d.s. for 8 weeks and IV placebo for 5 weeks<br><br>Comparator 2: Oral and IV placebo	Changes in maximal oxygen consumption	Changes in hemoglobin and iron stores
Beguin et al,[52] 2013	Multicenter RCT (Belgium)	Age 16-70 years, received autologous stem cell transplant for multiple myeloma or lymphoma, ferritin >100 ng/mL<br><br>n = 72	Iron sucose 200mg on days 28, 42 and 56 after stem cell transplant + EPO	Comparator 1: EPO 300 mcg given at day 28 post-stem cell transplant followed by 2-weekly intervals until day 112 post-transplant<br><br>Comparator 2: no i.v or EPO	Proportion with complete correction of anaemia (Hb >13g/dL) before day 126 post-transplant	(i) Median time to increase Hb level by >2 g/dL; (ii) proportion of responders (>2 g/dL) before day 126; (iii) transfusion requirements; (iv) adverse events; (v) quality of life using FACIT-F questionnaires
Bencaiova et al,[53] 2009	Single center RCT (Switzerland)	Non-anaemic pregnant women between 15 and 20 weeks' gestation<br><br>n = 260	Iron sucrose (1st phase - 400mg, 2nd phase - 600mg)	Ferrous sulphate 80mg daily until delivery	Percentage of pregnant women prior to delivery with Hb <11 g/dL	(i) changes in Hb and ferritin; (ii) birth weight; (iii) gestational age at birth; (iv) transfusion requirements; (v) hospital LOS after delivery
Bernabeu-Wittel et al,[54] 2016	Multicentre RCT (n = 13) (Spain)	Age >65 year, requiring hip fracture surgery with Hb 9-12 g/dL	Intervention 1: Ferric	s.c. placebo and 0.9% saline IV	RBC transfusion requirements within 60 days	(i) changes in Hb levels 24 and 72 hours after surgery; (ii) mortality; (iii) adverse

© 2021 Shah AA et al. *JAMA Network Open.*

		n = 306	carboxymaltose 1000mg and EPO<br><br>Intervention 2: Ferric carboxymaltose 1000mg + s.c placebo	placebo (opaque infusion kits)		events; (iv) HRQoL; (iv) medical complications (MI, DVT, stroke, infection, delirium)
Bhandal et al,[55] 2006	Single center RCT (UK)	Postpartum IDA, defined as Hb <9 g/dL and ferritin <15 mcg/L at 24-48 hours post-delivery)<br><br>n = 44	Iron sucrose 400mg split over 2 doses	Ferrous sulphate 200mg x2/day over 6 weeks	Primary outcome not specified but study outcomes included changes in Hb, ferritin and adverse events	
Bhavi et al,[56] 2017	Single center RCT (India)	Pregnant women between 14 and 34 weeks' gestation, Hb 7-11 g/dL, ferritin <15ng/ml<br><br>n = 112	Iron sucrose 200mg - total administered over multiple doses as per Ganzoni formula	Ferrous fumarate 100mg daily for 4 weeks	Changes in hemoglobin and iron stores	Safety and tolerability
Biboulet et al,[57] 2018	Single center RCT (France)	Scheduled for elective hip or knee arthroplasty with Hb between 10 and 13 g/dL<br><br>n = 100	Ferric carboxymaltose 1000mg (single dose) and 40,000 IU Eprex weekly for 3 weeks prior to surgery	Ferrous sulphate 160mg x2/day until day of surgery and 40,000 IU Eprex weekly for 3 weeks prior to surgery	Hb the day before surgery	(i) Changes in ferritin; (ii) Erythrocyte mass the day before surgery; (iii) Postoperative Hb
Bielza et al,[58] 2020	Single center RCT (Spain)	Age >70 years scheduled to undergo hip fracture surgery<br><br>n = 153	Iron sucrose 200mg on days 1, 3 and 5 of admission	0.9% saline IV placebo (opaque infusion kits)	Difference in absolute functional gain defined as Barthel's Index at discharge minus BI on admission	(i) Functional recovery at 3 months; (ii) mortality at 3,6 and 12 months; (iii) transfusion requirements; (iv) Hb at 3 months; (v) nosocomial infection;

© 2021 Shah AA et al. *JAMA Network Open.*

						(vi) Postoperative delirium
Birgegard et al,[60] 2010	Single center RCT (Sweden)	Blood donors with at least 5 previous blood donations,<br><br>n = 120	Iron sucrose 200mg after each donation	Iron sulphate 100mg once daily for 20 days after each donation	Iron status at the end of the study	(i) iron status during the study; (ii) RLS frequency and severity
Birgegard et al,[59] 2016	Multicenter RCT (n = 47) (Asia, USA, Europe)	Age >18 years, Non-myeloid malignancies on chemotherapy, Hb <120 g/dL, Tsat <50%, ferritin <800 mcg/L<br><br>n = 350	Iron isomaltoside (amount as per Ganzoni formula) single dose	Iron sulfate 100mg x2/day for 12 weeks	Change in hemoglobin from baseline to 4 weeks post-treatment	(i) Changes in hemoglobin, serum ferritin, TIBC, Tsat and iron levels; (ii) Changes in quality of life from baseline; (iii) adverse drug reactions
Bisbe et al,[61] 2014	Single center RCT (Spain)	Adults >18 years, undergoing total knee arthroplasty, postoperative Hb <12 g/dL or Tsat <20%<br><br>n = 122	Ferric carboxymaltose (amount as per Ganzoni formula) single dose	Ferrous sulphate 100mg once daily for 21 days	(i) Change in hemoglobin from post-operative day 4 to day 30; (ii) percentage of patients without anaemia (Hb >12g/dL)	(ii) Hb at day 30; (ii) changes in quality of life (EQ-5D and BI); (iii) distance in 6-MWT; (iv) percentage of transfused patients; (v) adverse events (including infection)
Boomershine et al,[62] 2016	Single center RCT (USA)	Age >18 years with fibromyalgia, Hb <13 g/dL<br><br>n = 81	Ferric carboxymaltose (15mg/kg), given over two doses	0.9% saline IV placebo (opaque infusion kits)	Proportion of patients with >13-point improvement from baseline to day 42 in FIQR score	(i) Changes in Hb and iron stores; (ii) changes in vital signs; (iii) adverse events
Breymann et al,[63] 2008	Multicenter RCT (n = 20) (Europe)	Postpartum anaemia with Hb <10.5 g/dL<br><br>n = 349	Ferric carboxymaltose 1000mg	Ferrous sulfate 100mg x2/day for 12 weeks	Change from baseline Hb to week 12 post-treatment	(i) Changes in iron profiles; (ii) transfusion requirements; (iii) proportion of patients who achieved Hb

© 2021 Shah AA et al. *JAMA Network Open.*

						levels of 12-16 g/dL; (iv) safety
Breymann et al,[64] 2017	Multicenter RCT (n = 29) (International)	Age >18 years, 2nd or 3rd trimester pregnancy with iron deficiency anaemia (Hb <11 g/dL, ferritin <20 ng/mL)<br><br>n = 252	Ferric carboxymaltose 1000-1500mg	Oral ferrous sulphate	Change in Hb from baseline to 3 weeks post-treatment	(i) Changes in Hb and iron profiles; (b) proportion of women who achieved anaemia correction; (c) time to anaemia correction; (d) changes in HRQoL (SF-36)
Burden et al,[65] 2015	Single center RCT (UK)	Elite athletes with non-anaemic iron deficiency (ferritin <30 mcg/L for females; <40 mcg/L for men)<br><br>n = 15	Ferric carboxymaltose 500mg single dose	0.9 saline IV placebo (opaque infusion kits)	Changes in hepcidin and iron profiles in response to iron treatment up to 4 weeks post-treatment	(i) Changes in Hb and t-Hb mass; (ii) changes in aerobic capacity
Burns et al,[66] 1996	Single center RCT (USA)	Hospitalised patients requiring total parenteral nutrition, iron deficiency defined as Fe-to-TIBC ratio <10%<br><br>n = 23	Iron dextran 10mg supplemented to TPN for 7 days	No iron	Not specified. Outcome measurements included changes in Hb, iron profiles, transfusion requirements and infection	
Charles-Edwards et al,[67] 2019	Single center RCT (UK)	Age >30 years, Symptomatic chronic stable heart failure, ferritin <100 mcg/L or 100-300 mcg/L with Tsat <20%<br><br>n = 40	Iron isomaltoside 1000mg	0.9% saline IV placebo (opaque infusion kits)	Change in skeletal muscle energetics two weeks post-treatment	(i) Changes in 6MWT; (ii) peak oxygen consumption; (iii) Hb and iron profiles; (iv) NYHA symptoms; (v) LVEF
Charytan et al,[69] 2005	Multicenter RCT (n = 16) (USA)	Age >18 years, non-dialysis dependent CKD, Hb <10.5 g/dL, Tsat <15%, ferritin <300 ng/mL<br><br>n = 96	Iron sucrose 200mg on days 1, 8, 15, 22 and 29	Ferrous sulfate 325mg x3/day	Mean changes in Hb and ferritin from baseline to day 43	(i) Changes in Tsat; (ii) number of patients achieving Hb >11 g/dL; (iii) adverse events

© 2021 Shah AA et al. *JAMA Network Open.*

Charytan et al,[68] 2012	Multicenter RCT (n = 56) (USA)	Adults aged 18-85 years, 3/12 history non-dialysis dependent CKD (Hb <11.5 g/dL) or dialysis dependent CKD (Hb <12.5 g/dL)<br><br>n = 254	Ferric carboxymaltose 15mg/kg (max 1g) in non-dialysis dependent CKD; 200mg in dialysis dependent CKD	Usual medical care<br><br>Both groups received EPO 2000 U	Safety of maximum administered dose	(a) Proportion of patient receiving Hb increase of >1g/dL; (b) proportion of patients achieving an Hb >12.0 g/dL ; (b) Mean changes in Hb, ferritin, Tsat
Cho et al,[70] 2016	Single center RCT (Korea) Crossover trial	Age >18 years, Primary restless legs syndrome, no co-morbidities<br><br>n = 65	Ferric carboxymaltose 1000mg	0.9% saline IV placebo (opaque infusion kits)	Change from baseline in the International RLS Severity scale at week 6 post-treatment	(i) Change in sleep (Pittsburg Sleep Quality Index) and health-related quality of life (SF-36)
Cho et al,[71] 2018	Single center RCT (Korea)	Restless leg syndrome, International RLS score >15, ferritin <300 ng/mL, Hb >12 g/dL, Tsat <45%<br><br>n = 65	Ferric carboxymaltose 500mg	0.9% saline IV placebo (opaque infusion kits)	Change from baseline in IRLS score at week six post-randomisation	(i) Changes in SF-36 and sleep quality
Coyne et al,[72] 2007	Multicenter RCT (n = 37) (USA)	Age >18 years, CKD on HD for >90 days, ferritin <1200 ng/mL, Tsat <25%, Hb <11 g/dL, established on EPO<br><br>n = 134	Ferric gluconate 1g adminstered over 8 consecutive doses of 125mg	Usual care	Change in Hb from baseline to week 6	(i) Percentage of with Hb response >2 g/dL; (ii) changes in iron profiles; (iii) safety
Dalal et al,[73] 2008	Single center RCT (India)	Pregnant women (26-34 weeks' gestation) with anaemia, Hb 7-11 g/dL<br><br>n = 150	Iron sucrose 200mg weekly - calculated to match iron deficit	Ferrous sulphate 100mg x2/day until delivery	Changes in Hb from baseline to 2 weeks, 4 weeks and delivery	(i) Safety; (ii) maternal and fetal outcomes
Damineni et al,[74] 2016	Single center RCT (India)	Postpartum anaemia, defined as Hb 7-10 g/dL<br><br>n = 90	Ferric carboxymaltose 1000mg	Ferrous ascorbate 100mg x2/day for 6 weeks	Change in Hb at 6 weeks post-treatment	(i) Proportion of patients with rise in Hb >3 g/L; (ii) tolerability and compliance

© 2021 Shah AA et al. *JAMA Network Open.*

Dangsuwan et al,[75] 2010	Single center RCT (Thailand)	Age 20-65 years, gynaecology malignancy who have undergoing surgery and receiving chemotherapy, Hb <10 g/dL<br><br>n = 44	Iron sucrose 200mg one dose	Ferrous sulphate 200mg x3/day	Incidence of RBC transfusion in subsequent chemotherapy cycle	(i) Changes in Hb; (ii) no. of RBC units; (iii) adverse events; (iv) QoL scores measured by FACT-An
Darwish et al,[76] 2019	Single center RCT (Egypt)	Pregnant women gestational >14 weeks, Hb 7-10 g/dL, ferritin <12mg/L<br><br>n = 66	Iron dextran, amount as per Ganzoni formula over multiple doses	Ferrous fumarate 60mg x3/day for 4 weeks	Change in Hb from baseline to 4 weeks	(i) Changes in iron stores; (ii) adverse effects
Deeba Shafi et al,[83] 2012	Single center RCT (India)	Pregnant women 28-37 weeks' gestation with established iron deficiency anaemia (Hb 6-10 g/dL and Ferritin <15ng/ml)<br><br>n = 200	Iron sucrose 200mg on alternate days, calculated to achieve Hb of 12 g/dL	Ferrous ascorbate 200mg daily	Hb at 2, 4 and 6 weeks post-treatment	Ferritin at 2, 4 and 6 weeks post-treatment
Deng et al,[77] 2017	Single center RCT (China)	CKD patients on haemodialysis with RLS, ferritin <200 ng/mL, Tsat <20%<br><br>n =32	Iron sucrose 100mg x3/week until total dose of 1000mg	0.9% saline IV placebo (opaque infusion kits)<br><br>Both groups received EPO	Changes in International RLS scores at two weeks post-treatment	(i) Changes in Hb and iron status; (ii) safety and adverse events
Drexler et al,[78] 2020	Single center RCT (Austria)	Iron deficient (ferritin <30 mcg/L) blood donors<br><br>n = 176	Ferric carboxymaltose 1000mg	Ferrous sulphate 100mg (x3 per week) for 10 weeks	Change in Tsat (%) at 8-12 weeks post-randomisation	(i)Changes in other markers of iron status and Hb; (ii) adverse events; (iii) tolerance; (iv) symptoms of restless leg syndrome, HRQoL and fatigue
Dubey et al,[79] 2016	Single center RCT (India)	Pregnant women gestation age 20-34 weeks, Hb 7-9 g/dL, Ferritin <15 ng/mL<br><br>n = 200	Iron sucrose calculated to target Hb of 11 g/dL	Oral iron 100mg x3/day throughout pregnancy	Changes in Hb on day 14 and day 28 post-randomisation	(i) Changes in reticulocyte count and ferritin; (ii) side effects; (iii) proportion of women achieving Hb >11 g/dL; (iv) neonatal outcomes; (v)

© 2021 Shah AA et al. *JAMA Network Open.*

						transfusion requirements
Edwards et al,[80] 2009	Single center RCT (UK)	Adults scheduled to undergo bowel resection for colorectal cancer<br><br>n =62	Iron sucrose 600mg split over 2 doses	0.9% saline IV placebo (opaque infusion kits)	Change in Hb between recruitment and day of admission	(i) RBC transfusion rate; (ii) changes in iron profiles; (iii) hospital LOS; (iv) adverse events
El Khouly et al,[81] 2017	Single center RCT (Egypt)	Postpartum iron deficiency anaemia, Hb <9 g/dL and ferritin < 15 mcg/L<br><br>n = 352	Iron sucrose 200mg x3/week	Oral ferrous sulphate 150mg x2/day for 6 weeks	Changes in Hb and ferritin up to day 60 postpartum	Safety, tolerability and compliance
Evstatiev et al,[82] 2013	Multicenter RCT (n = 69) (Europe)	Age >18 years, Non-anaemic patients with IBD<br><br>n = 206	Ferric carboxymaltose 500mg	0.9% saline IV placebo (opaque infusion kits)	Time to occurrence of anaemia (Hb <12 g/dL in females and 13 g/dL in males)	(i) Proportion of patients with anaemia at 2,4,6 and 8 months post-treatment; (ii) changes in HRQoL (SF-36); (iii) changes in Crohn's disease activity
Favrat et al,[84] 2014	Multicenter RCT (n = 21) (Europe)	Women aged>18 years (premenopausal), symptomatic fatigue, borderline anaemia (Hb >11.5 g/dL) with evidence of iron deficiency (ferritin <15 mcg/L or ferritin <50 mcg/L and Tsat <20%)<br><br>n = 290	Ferric carboxymaltose 1000mg	0.9% saline IV placebo (opaque infusion kits)	Proportion of patients who improved Piper Fatigue Score by >1 from baseline to day 56	(i) Changes in PFS total and subscale score and SF-12 QoL score; (ii) correction of anaemia and iron deficiency; (iii) adverse events
Ferrer-Barcelo et al,[85] 2019	Single center RCT (Spain)	Age >18 years, non-variceal acute upper gastrointestinal bleed (Hb <10 g/dL on day of hospital discharge)<br><br>n = 61	Ferric carboxymaltose 1500-2000mg	Ferrous sulphate 325mg x2/day for 6 weeks	Resolution of anaemia at day 42 (men, Hb >13 g/dL; women, Hb >12 g/dL)	(i) Partial response (Hb rise >2 g/dL); (ii) changes in iron profiles; (iii) adverse events

© 2021 Shah AA et al. *JAMA Network Open.*

Fishbane et al,[86] 1995	Single center RCT (USA)	Adults with CKD on haemodialysis for atleast 3 months, receiving EPO, serum ferritin >100 ng/mL, Tsat >15%<br><br>n = 28	Iron dextran 200mg twice weekly	Ferrous sulphate (325mg x3/day) or iron polysaccharide (150mg x2/day)	Not specified. Outcome measurements included haematocrit, ferritin and Tsat	
Friel et al,[87] 1995	Single center RCT (Canada)	Low birth weight newborns<br><br>n = 26	Iron dextran 250 mcg/kg/day	No iron	Not specified. Outcome measurements included haematologic and biochemical parameters, infection rates, red blood cell haemolysis and iron balance	
Froessler et al,[88] 2013	Single center RCT (Australia)	Women with iron deficiency anaemia (Hb <110 g/L and ferritin <12 mcg/L), antenatal between 28-36 weeks' gestation, or within 72 hours of birth or delivery with blood loss >500 mls.<br><br>n = 271	Iron sucrose 400mg (divided into 2 doses)	Ferrous sulphate 160mg daily for 6 weeks postpartum or until delivery (if started antenatal)	Incidence of severe anaemia (Hb<8 g/dL) prior to hospital discharge	RBC transfusion requirements
Froessler et al,[89] 2016	Single center RCT (Australia)	Age >18 years scheduled for abdominal surgery, Hb <12 g/dL (females) and <13 g/dL; (males), ferritin <300 mcg/L, Tsat <25%<br><br>n = 72	Ferric carboxymaltose 15mg/kg pre-operatively, and 0.5 mg per 1mL of blood loss postoperatively	Usual medical care	Incidence of RBC transfusion	(i) Changes in Hb from randomisation to admission; (ii) ICU admission; (iii) perioperative morbidity (infection, respiratory failure, renal impairment, DVT); (iv) discharge Hb; (v) length of stay; (vi) 30-day mortality; (vii) HRQoL scores

© 2021 Shah AA et al. *JAMA Network Open.*

Garrido-Martin et al,[90] 2012	Single center RCT (Spain)	Age >18 years, elective cardiac surgical ICU (postoperative)<br><br>n = 159	Iron sucrose 300mg (given over 3 separate doses) and oral placebo	Comparator 1: Ferrous fumarate 105mg daily for 1 month and IV placebo<br><br>Comparator 2: IV and oral placebo	Change in Hb from baseline up to day 30 post-hospital discharge	(i)changes in iron profiles; (ii) postoperative transfusion requirements
Grote et al,[91] 2009	Multicenter RCT (n = 3) (Sweden)	Age 18-70 years, Restless leg syndrome, ferritin <45 mcg/L<br><br>n = 60	Iron sucrose 200mg (x5 doses over 3 weeks)	0.9% saline IV placebo (opaque infusion kits)	Between group difference in International RLS score at the 11-week follow-up visit	(i) Treatment effects on daytime sleepiness; (ii) adverse events; (iii) changes in Hb and iron status
Gupta et al,[92] 2014	Single center RCT (India)	Singleton pregnancy between 24-34 weeks' gestation, Hb 7 - 9 g/dL and ferritin <15 ng/mL<br><br>n = 100	Iron sucrose calculated to target Hb of 11 g/dL	Ferrous sulphate 200 mg x3/day for 4 weeks	Increase in hemoglobin and ferritin over 4 weeks post-treatment	(i) Reticulocyte count rise; (ii) mean Hb at the time of delivery; (iii) side effect; (iv) perinatal outcomes
Hedenus et al,[93] 2007	Multicenter RCT (n = 15) (Sweden)	Adults with Non-Hodgkin's Lymphoma, Chronic Lymphocytic Leukaemia or myeloma, Hb 9-11 g/dL, evidence of stainable iron on bone marrow aspirate within 1 month prior to inclusion<br><br>n = 67	Iron sucrose 100mg weekly from weeks 0 to 6, every second week from weeks 8 to 14	No iron	Mean change in Hb from baseline to end of treatment	(i) Hb response (increase >2 g/dL); (ii) time to Hb response; (iii) changes in iron profiles; (iv) EPO requirements
Hedenus et al,[94] 2014	Multicenter RCT (n = 11) (Europe)	Adults with lymphoid malignancies, Hb 8.5-10.5 g/dL, Tsat <20% and ferritin >30 ng/mL, received chemotherapy for >8 weeks prior to inclusion<br><br>n = 17	Ferric carboxymaltose 1000mg	Usual medical care	Mean change in Hb from baseline to week 8 post-treatment	(i)Safety; (ii) Hb response (increase >1 g/dL) and correction (Hb >11 g/dL)

© 2021 Shah AA et al. *JAMA Network Open.*

Henry et al,[95] 2007	Multicenter study (USA)	Age >18 years, due to start chemotherapy for a non-myeloid malignancy, Hb <11 g/dL, ferritin >100 ng/ml or Tsat >15%.<br><br>n = 187	Ferrous gluconate IV 125mg once weekly for 8 weeks	Comparator 1: Ferrous sulphate 325mg t.d.s<br><br>Comparator 2: No iron	Mean change in Hb from baseline to endpoint, first RBC transfusion or study withdrawal	(i)Hb response (>2 g/dL); (ii) safety
Holm et al,[96] 2017	Single center RCT (Denmark)	Postpartum haemorrhage >700 ml and <1000 ml, or PPH >1000ml and Hb >6.5 g/dL at least 12 hours after delivery<br><br>n = 196	Iron isomaltoside 1200mg, single dose	Oral iron 100mg daily	Change in physical fatigue scores of MFI questionnaire within 12 weeks postpartum	(i) Changes in Hb, ferritin, iron; (ii) changes in other dimensions of MFI and symptoms of depression; (iii) time to lactogenesis; (iv) time to discontinuation of breastfeeding; (v) transfusion
Iyoke et al,[97] 2017	Multicenter RCT (n = 2) (Nigeria)	Postpartum anaemia Hb 6 - 7.9 g/dL, red cell features of iron deficiency at 48 hours or later following vaginal delivery<br><br>n = 284	Iron dextran, calculated as hemoglobin required to reach 10 g/dL multiplied by 250 mg of parenteral iron	100mg oral elemental iron for 6 weeks	Proportion of women who attained a Hb of at least 10 g/dL by 6 weeks postpartum	
Jain et al,[98] 2013	Single center RCT (India)	Postpartum Hb <8 g/dL within 48h of delivery<br><br>n = 40	Iron sucrose calculated to total iron deficit, given over three separate doses in one weeks	Ferrous fumarate 300 mg daily for 14 days	Change in Hb at14 days postpartum	Safety and tolerability
Johansson et al,[99] 2015	Single center RCT (Denmark)	Age >18 years, undergoing elective or subacute CABG, valve or combination without anaemia (Hb >12 g/dL for women, Hb >13 g/dL for men)	Iron isomaltoside 1000mg once only	0.9% saline IV placebo (opaque infusion kits)	Less decrease in postoperative Hb level in non-anaemic patients undergoing cardiac surgery	(i)RBC transfusion requirements; (ii) changes in iron profiles; (iii) safety

© 2021 Shah AA et al. *JAMA Network Open.*

		n = 60				
Kalra et al,[100] 2016	Multicenter RCT (n = 67) (Europa, Asia, USA)	Age >18 years, Hb <11 g/dL, serum ferritin mcg/L, Tsat <20%, not received EPO in previous 8 weeks<br><br>n = 351	Iron isomaltoside calculated according to Ganzoni formula	Iron sulphate 100 mg x2/day for 8 weeks	Change in Hb from baseline from baseline to week 4 post-randomisation	(i)Change in Hb from weeks 2 to 8; (ii) changes in iron profiles; (iii) changes in HRQoL from baseline to weeks 4 and 8; (iv) safety
Karkouti et al,[101] 2006	Single center RCT (Canada)	Adults >18 years, postoperative open-heart surgery, total hip arthroplasty or spinal fusion, Hb 70-90 g/dL<br><br>n = 38	Intervention 1: Iron sucrose 600mg (given over 3 split doses)<br><br>Intervention 2: Iron sucrose 600mg (given over 3 split doses) and EPO (1200 IU/kg)	0.9% saline IV placebo (opaque infusion kits)	Change in Hb from postoperative day 1 to day 7	(i) Hb up to 6 weeks postoperatively; (ii) changes in iron profiles on day 7; (iii) RBC transfusion requirements; (iv) hospital length of stay; (v) changes in HRQoL (SF-36)
Kasper et al,[102] 1998	Single center RCT (Germany)	Age >18 years, Hb <12.0 g/dL, scheduled to undergo major surgery and eligible for autologous blood donation<br><br>n = 108	Ferric gluconate 102.5 mg IV (once) and oral iron (ferric succinate) 92.5mg x3/day for 21 days	Oral iron (ferric succinate) 92.5mg x3/day for 21 days and IV placebo	Improvement in Hb during the study period	(i) Changes in iron profiles; (ii) volume of blood donated; (iii) adverse events
Keeler et al,[103] 2017	Multicenter RCT (n = 7) (UK)	Scheduled to undergo colorectal cancer excision with anaemia, defined as Hb <11 g/dL in women and <12 g/dL in men<br><br>n = 116	Ferric carboxymaltose 1000mg, stratified on Hb (if Hb <10 g/dL, 2000mg could be given	Ferrous sulphate 200mg x2/day until surgery	Transfusion requirements from preoperative recruitment until postoperative outpatient review	(i) Changes in hemoglobin and haematinic profiles; (ii) postoperative complications; (iii) RBC transfusion requirements

© 2021 Shah AA et al. *JAMA Network Open.*

Keller et al,[104] 2020	Single center RCT (Switzerland)	Blood donors with evidence of non-anaemic iron deficiency (ferritin <50 mcg/L)<br><br>n = 203	Ferric carboxymaltose 800mg	0.9% saline IV placebo (opaque infusion kits)	Self-rated average fatigue during the past 7 days, assessed 6-8 weeks post-randomisation	(i) Changes in other self-reported fatigue and HRQoL measures; (ii) changes in Hb and ferritin; (iii) safety reporting
Khalafallah et al,[105] 2010	Single center RCT (Australia)	Pregnant women <28 weeks' gestation with Hb 8.5 – 11.5 g/dL and ferritin <30 mcg/L<br><br>n = 200	Iron polymaltose, dose calculated according to weight and target Hb	Ferrous sulphate 80mg daily until delivery	Predelivery Hb and iron stores	Safety, tolerability and compliance
Khalafallah et al,[107] 2016	Multicenter RCT (n = 2) (Australia)	Age >18 years, requiring major elective surgery with at least planned hospital stay of 2 nights, Hb 70-120 g/L, ferritin <100 or Tsat 20%<br><br>n = 201	Ferric carboxymaltose 1000mg	Usual medical care	Changes in Hb and iron stores at 4 weeks postoperatively	(i) Safety and tolerability; (ii) length of hospital stay; (iii) infections; (iv) HRQoL at 4 and 12 weeks postoperatively
Khalafallah et al,[106] 2018*	Single center RCT (Australia)	Pregnant women in second/third trimester of pregnancy, Hb 8.5-12 g/dL, ferritin <100 mcg/L or Tsat <20%<br><br>n = 166	Intervention 1: Ferric carboxymaltose 1000mg<br><br>Intervention 2: Iron polymaltose 1000mg	Ferrous sulphate 105mg daily until delivery	Change in Hb and ferritin from baseline to 4 weeks post-randomisation	(i) Tolerability and adverse events; (ii) transfusion requirements; (iii) changes in HRQoL; (iv) fetal outcomes
Kim et al,[110] 2007	Single center RCT (Korea)	Age>18 years, Hb <12 g/dL, undergoing chemotherapy for cervical cancer<br><br>n = 75	Iron sucrose 200mg at each chemotherapy cycle	Usual medical care	RBC transfusion requirements	(i) Changes in Hb; (ii) proportion of patients with resolution of anaemia (defined as Hb >12 g/dL)
Kim et al,[109] 2009	Multicenter RCT (n = 3) (South Korea)	Women experiencing menorrhagia with Hb <9 g/dL, scheduled to undergo elective surgery	Iron sucrose 200mg 3 days a week, for 3 weeks prior to surgery	Iron succinylate 80mg once daily for 3 weeks prior to surgery	Changes in Hb preoperatively	Safety

© 2021 Shah AA et al. *JAMA Network Open.*

		n = 76				
Kim et al,[111] 2017	Multicenter RCT (n = 7) (South Korea)	Adults (age >20 years) with acute isovolemic anaemia (Hb 7-10 g/dL) at 5-7 days post-gastrectomy<br><br>n = 454	Ferric carboxymaltose 1000mg	0.9% saline IV placebo (opaque infusion kits)	No. of hemoglobin responders (Hb rise >2 g/dL) at 12 weeks post-randomisation	(i) Percentage of patients with Hb >10 g/dL, 11 g/dL and 12 g/dL by weeks 3 and 12; (ii) self-reported HRQoL; (iii) changes in Hb and iron profiles over the study duration
Kochhar et al,[112] 2013	Single center RCT (India)	Singleton pregnant women between 24-34 weeks' gestation, moderate iron deficiency anaemia (Hb 7 -9 g/dL, MCV <85 fl, ferritin <15 ng/mL)<br><br>n = 100	Iron sucrose, calculated to total iron deficit and targeted to Hb >12 g/dL, given over three separate doses	Ferrous sulphate 200 mg x3/day for 4 weeks	Change in Hb from baseline up to day 30 post-randomisation	(i) Requirement for RBC transfusion; (ii) adverse events; (iii) neonatal outcomes
Krayenbuehl et al,[113] 2020	Multicenter RCT (n = 4) (Switzerland)	Premenopausal, menstruating women aged 18 years with fatigue and non-anaemic iron deficiency (Hb >12 g/dL, ferritin <50 mcg/L)<br><br>n = 90	Iron sucrose 800 mg over multiple doses)	0.9% saline IV placebo (opaque infusion kits)	Changes in fatigue scores 6 weeks after treatment initiation	(i) Changes in Hb and ferritin; (ii) Safety
Kulnigg et al,[114] 2008	Multicenter RCT (n = 36) (Europe and South America)	IBD with iron deficiency anaemia (Hb <10 g/dL and Tsat <20% or ferritin <100 mcg/L)<br><br>n = 200	Ferric carboxymaltose, dose calculated according to Ganzoni formula	Ferrous sulphate 100mg x2/day for 12 weeks	Change in Hb from baseline to week 12	(i) Changes in Hb and iron profiles from baseline to weeks 2, 4 and 8; (ii) treatment responses, defined as Hb rise >2 g/dL and/or proportion of participants with resolution of anaemia; (iii) tolerability

© 2021 Shah AA et al. *JAMA Network Open.*

Kulnigg-Dabsch et al,[115] 2013	Multicenter RCT (Austria and Germany)	IBD associated thrombocytosis and Hb >10.5 g/dL, age 18 and 60 years, ferritin <100 ng/mL, Tsat <30%<br><br>n = 26	Intervention 1: Ferric carboxymaltose 500mg single dose<br><br>Intervention 2: Ferric carboxymaltose 1000mg single dose	0.9% saline IV placebo (opaque infusion kits)	Decrease in platelet count of at least 25% at 6 weeks	(i) Effect on ferric carboxymaltose on platelet count, disease activity and HRQoL; (ii) safety at week 6
Kuo et al,[116] 2008	Multicenter RCT (n = 2) (Taiwan)	CKD on long-term haemodialysis, ferritin <800 mcg/L<br><br>n = 110	Iron sucrose 100mg once weekly for 12 weeks	0.9% saline IV placebo	Not specified. Outcomes measurements included: (i) oxidative damage to peripheral blood lymphocytes; (ii) changes in ferritin	
Li et al,[117] 2008	Single center RCT (China)	CKD on maintenance haemodialysis, Hb 6-9 g/dL, ferritin <500 mcg/L, Tsat <30%<br><br>n = 46	Iron sucrose 100mg twice a week for 8 weeks, once a week for 4 weeks after	Ferrous succinate 200mg x3/day for 12 weeks	Increase in Hb >3 g/dL or Hct >10% during therapy	
Lindgren et al,[118] 2009	Multicenter RCT (n = 11) (Sweden)	Age 18-85 years, IBD, Hb <11.5 g/dL, ferritin <300 mcg/L<br><br>n = 91	Iron sucrose 1000mg split over 200mg doses on alternate days	Ferrous sulphate 200mg x2/day over 20 weeks	Hb response (>2 g/dL) at 20 weeks post-treatment	
Litton et al,[108] 2016	Multicenter RCT (n = 4) (Australia)	Age >18 years, within 48hrs of ICU admission, anticipated to require ICU beyond next calendar day, Hb <100 g/L at any time in the preceding 24 hours<br><br>n = 140	Ferric carboxymaltose 500mg, redosing 4 days later if still eligible (max 4 doses allowed)	0.9% saline IV placebo (opaque infusion kits)	Number of RBCs transfused per patient between randomisation and hospital discharge	(i) Hb at hospital discharge; (ii) ICU and hospital LOS; (iii) proportion of patients receiving a transfusion; (iv) mortality; (v) infection

© 2021 Shah AA et al. *JAMA Network Open.*

Maccio et al,[119] 2010	Single center RCT (Italy)	Age >18 years, advanced cancer, Hb <10 g/dL, ferritin 100-800 ng/ml, Tsat >15%<br><br>n = 148	Ferric gluconate 125mg once weekly for 12 weeks	Oral lactoferrin 200mg daily for 12 weeks	Change in Hb from baseline until end of study period	(i) Hb response (Hb rise of >2 g/dL or Hb >12 g/dL); (ii) changes in iron profiles, CRP and ESR
Macdougall et al,[121] 1996	Single center RCT (UK)	CKD on regular haemodialysis, Hb <8.5 g/dL, ferritin 100-800 mcg/L<br><br>n = 37	Iron dextran 250mg every two weeks	Comparator 1: Ferrous sulphate 200mg x3/day<br><br>Comparator 2: No iron	Changes in Hb, iron status and EPO dosage requirements from baseline until end of study period (16 weeks)	
Macdougall et al,[120] 2014	Multicenter RCT (n = 193) (USA, Europe)	Age >18 years, non-dialysis CKD, Hb 9-11 g/dL, ferritin <100 ng/ml or <200 ng/mL with Tsat 20%, eGFR <60<br><br>n = 626	Ferric carboxymaltose adjusted to target ferritin in two groups: (i) High: 400-600; (ii) Low: 100-20	Ferrous sulfate 304mg x2/day for 52 weeks	Time to initiation of other anaemia management of occurrence of an Hb trigger (Hb <10 g/dL during weeks 8-52)	(i) Changes in Hb and iron profiles; (ii) transfusion requirements (iii) safety
Madi-Jebara et al,[122] 2004	Single center RCT (Lebanon)	Postoperative cardiac surgery critical care, Hb 7-10 g/dL<br><br>n = 120	Iron sucrose 200mg daily until calculated total iron deficit reached	0.9% saline IV placebo (opaque infusion kits)	Change in Hb from baseline until postoperative day 30	(i) Changes in iron profiles; (ii) RBC transfusion requirements; (iii) In-hospital mortality
McMahon et al,[123] 2010	Multicenter RCT (n = 6) (Australia)	Age 18-80 years, CKD stages 3-5, Hb >11 g/dL, ferritin <300 mcg/L, Tsat <25%<br><br>n = 100	Iron sucrose 200mg every 2 months to maintain ferritin between 300-500 mcg/L	Ferrous sulphate 105mg daily to maintain ferritin between 100-150 mcg/L	Changes in Hb at 12 months or at termination (e.g. starting dialysis, adverse event, starting ESA)	(i) Changes in renal function; (ii) HRQoL; (iii) time to commencement of dialysis/ESA
Meyer et al,[124] 1996	Single center RCT (South Africa)	Pre-term infants (<33 weeks' gestation, <1.5kg), venous Hct <0.38<br><br>n = 42	Iron sucrose 6mg/kg per week	Ferrous sulphate 12 mg/kg/day	Specific a priori endpoints not reported	

© 2021 Shah AA et al. *JAMA Network Open.*

Michael et al,[125] 2002	Multicenter RCT (n = 69) (USA)	Adults with CKD on chronic haemodialysis, established on EPO, Hb <12.3 g/dL, ferritin <800 ng/mL, Tsat <50%<br><br>n = 2534	Ferric gluconate 125mg	0.9% saline IV placebo (opaque infusion kits)	Drug intolerance and life-threatening adverse events	(i) Rate of all adverse events; (ii) cross-reactivity to previously exposed iron dextran
Montano-Pedroso et al,[126] 2018	Multicenter RCT (n = 2) (Brazil)	Adult women (18-55 years), post-bariatric surgery with a BMI <32 kg/m^2 and stabilised weight loss for at least 6 months<br><br>n = 56	Iron sucrose 400mg (given over two doses)	Iron hydroxide 100mg x2/day for 8 weeks	Hb at postoperative day 56	(i) Changes in iron profiles; (ii) adverse effects; (iii) postoperative complications on days 7,28 and 56; (iv) changes in HRQoL (SF-36 and FACIT-F)
Moppett et al,[127] 2019	Multicenter RCT (n = 2) (UK)	Age >70 years, emergency admission with hip fracture<br><br>n = 80	Iron sucrose 200mg on three separate occasions perioperatively	Usual medical care	Difference in mean reticulocyte count at day 7 after randomization	(i) Cardiovascular or infective complications; (ii) changes in Hb and iron parameters; (iii) length of stay; (iv) mortality; (v) transfusion requirements; (vi) postoperative mobility
Mudge et al,[128] 2012	Single center RCT (Australia)	Adults (age >18 years) scheduled to undergo living-donor or deceased donor kidney transplant surgery<br><br>n = 104	Iron polymaltose 500mg single dose on postoperative day 4	Ferrous sulphate 210 mg x2/day until Hb >11 g/dL	Time to Hb more than or equal to 11 g/dL	(i) Adverse events; (ii) acute rejection episodes; (iii) infections; (iv) blood transfusions or EPO
Na et al,[129] 2011	Single center RCT (South Korea)	Scheduled for bilateral total knee arthroplasty, Hb >10 g/dL, ferritin 100-300 mcg/L and Tsat <20%<br><br>n = 108	Iron sucrose 200mg – one dose intraoperatively, followed by single dose on post-op days 1,2,3 and 5 if postoperative Hb	No iron	Changes in postoperative Hb and RBC transfusion requirements up to 6 weeks postoperatively	Changes in iron profiles

© 2021 Shah AA et al. *JAMA Network Open.*

			was between 7-8 g/dL			
Nanthini et al,[130] 2017	Single center RCT (India)	Age 18-45 years, singleton pregnancy 12-32 weeks' gestation, Hb 7-9 g/dL<br><br>n = 127	Iron sucrose 48 hourly, dose calculated to iron deficit	Intramuscular iron every 8 weeks	Proportion achieving target Hb of 11 g/dL up to 8 weeks post-treatment	Safety and tolerability
Neeru et al,[131] 2012	Single center RCT (India)	Pregnant women 14-36 weeks' gestation, Hb 6.5-10.9 g/dL, ferritin <27 ng/dL<br><br>n = 100	Iron sucrose on alternate days, dose calculated to iron deficit	Ferrous fumarate 300mg	Efficacy defined as improvement in Hb by 1 g/dL at 4 weeks post-treatment	Tolerability
Neogi et al,[132] 2019	Multicenter RCT (n = 4) (India)	Pregnant women age >18 years, Hb 5-8 g/dL if gestation 20 and 28 weeks, Hb 5-9 g/dL if gestation >28 weeks<br><br>n = 2018	Iron sucrose on alternate days, dose calculated to target Hb	Oral iron 100mg x2/day until 6 weeks postpartum	Composite of postpartum haemorrhage, puerperal sepsis, shock, prolonged hospital LOS, need for transfusion, ICU admission and referral to another centre	(i) Components of maternal primary outcomes; (ii) composite fetal outcome (low birthweight, preterm labour, perinatal death); (iii) changes in Hb and ferritin
Ng et al,[133] 2018	Multicenter RCT (n = 2) (UK)	Adult with oesophageal cancer and treatment decision for palliative chemotherapy (Hb <12 g/dL in females, Hb <13 g/dL in men)<br><br>n = 27	Iron maltoside - dose calculated according to Ganzoni formula	Usual medical care	Feasibility (screen failure rates, acceptability, non-concordance, retention rates)	(i) Changes in Hb and iron profiles post-chemotherapy; (ii) transfusion requirements; (iii) mortality; (iv) HRQoL scores
Noronha et al,[134] 2018	Single center RCT (India)	Age >18 years, malignancy requiring chemotherapy, Hb <12 g/dL, ferritin <100 ng/mL or Tsat <20%<br><br>n = 192	Iron sucrose every 3 weeks, calculated as per iron deficit formula	Ferrous sulfate 100mg x3/day for 6 weeks	Change in hemoglobin from baseline to 6 weeks post-treatment	(i) Proportion of patients with Hb rise >1g/dL; (ii) RBC transfusion requirements; (iii) quality of life (FACT-An questionnaires); (iv) response to cancer

© 2021 Shah AA et al. *JAMA Network Open.*

						therapy and overall survival; (v) safety profile of iron
Okonko et al,[135] 2008	Multicenter RCT (n = 2) (UK and Poland)	Age >21 years, NYHA Class II or III, Hb <12.5 g/dL (anaemic group) or Hb 12.5 to 14.5 g/dL (non-anaemic group), ferritin <100 mcg/L or 100-300 mcg/L if Tsat <20%, LVEF <45%<br><br>n = 35	Iron sucrose administered every 4 weeks up to 16 weeks, calculated to iron deficit	Usual medical care	Change in absolute pVO2 from baseline to week 18 post-treatment	(i) Changes in Hb, iron profiles; (ii) changes in LVEF, NYHA class; (iii) changes in global HRQoL and fatigue
Olijhoek et al,[136] 2001	Multicenter RCT (n = 18) (Europe)	Age >18 years, scheduled to undergo orthopaedic surgery that was estimated to require 2 to 4 units of blood, Hb 10-13 g/dL, ferritin >50 mcg/L<br><br>n = 110	Intervention 1: EPO and iron saccharate 200mg on days 1 and 8<br><br>Intervention 2: Placebo and iron saccharate 200mg on days 1 and 8	Comparator 1: EPO + oral iron 200mg daily for 14 days<br><br>Comparator 2: Placebo + oral iron 200mg daily for 14 days	Total RBC production from day 1 to day 14 (day before scheduled surgery)	Changes in Hb
Onken et al,[137] 2014#	Multicenter RCT (n = 84) (USA)	Age >18 years, iron deficiency anaemia of varying aetiology (heavy uterine bleeding, GI disorders, nutritional deficiencies),<br><br>Hb <11 g/dL, ferritin <100 ng/mL or ferritin <300 ng/mL with Tsat <30%, who did not respond to a two-week run-in with oral iron<br><br>n = 507	Ferric carboxymaltose 30 mg/kg (max 1.5 kg)	Ferrous sulphate 325 mg x3/day for two weeks	Mean change from baseline to highest observed Hb	(i) Proportion of patients achieving Hb >12 g/dL; (ii) proportion of patients achieving an increase of at least 2 g/dL at any point (iii) changes in ferritin; (iv) adverse events
Padmanabhan et al,[138] 2018	Single center RCT (UK)	Scheduled for open heart surgery with WHO definition of	Ferric carboxymaltose 1000mg	Ferrous sulphate 200mg daily until surgery	Change in Hb 3 weeks after iron therapy	(i) Changes in iron profiles and CRP; (ii) transfusion

© 2021 Shah AA et al. *JAMA Network Open.*

		anaemia (male, Hb <13 g/dL; female, Hb <12 g/dL)<br><br>n = 50				requirements; (iii) postoperative complications (atrial fibrillation, renal failure, infection); (iv) HRQoL (EQ-5D-5L, SF-36)
Park et al,[139] 2019	Single center RCT (South Korea)	Scheduled to undergo elective hip or knee arthroplasty, Hb >10 g/dL within 4 weeks of surgery<br><br>n = 58	Ferric carboxymaltose 1000mg (following induction of anaesthesia)	0.9% saline IV placebo (opaque infusion kits)	Change in Hb from preoperative value to 1 month after surgery	(i) Changes in Hb and iron profiles from preoperative values to postoperative day 5; (ii) RBC transfusion requirements; (iii) changes in perioperative oxygenation; (iv) adverse events
Pedrazolli et al,[140] 2008	Multicenter RCT (n = 33) (Italy)	Adults with solid organ malignancy planned to have chemotherapy for 12 weeks, Hb <11 g/dL, ferritin>100 mcg/L and Tsat >40%<br><br>n = 149	Ferric gluconate 125mg per week for 6 weeks	No iron	Proportion of patients with response at 12 weeks (Hb rise >2 g/dL or Hb >12 g/dL)	(i) Time to response; (ii) transfusion requirements; (iii) changes in Hb over time; (iv) safety
Pieracci et al,[142] 2014	Multicenter RCT (n = 4) (USA)	Adults >18 years, critically ill trauma with Hb <12 g/dL, expected ICU length of stay >5 days<br><br>n = 150	Iron sucrose 100mg thrice weekly for up to six doses or until ICU discharge	0.9% saline IV placebo (opaque infusion kits)	Number of participants requiring an RBC transfusion	(i) Changes in iron profiles and markers of erythropoiesis; (ii) infection at 28 days; (iii) 28-day mortality
Perello et al,[141] 2014	Single center RCT (Spain)	Postpartum anaemia within 48 hours of delivery, Hb 6-8 g/dL<br><br>n = 72	Iron sucrose 200mg on two consecutive days and ferrous sulphate 105mg x2/day for 30 days	0.9% saline on two consecutive days (opaque infusion kits) and ferrous sulphate 105mg x2/day for 30 days	Changes in Hb at one, two and six weeks postpartum	(i) changes in iron profiles (ferritin, TIBC, retic count); (ii) Symptoms of anaemia, depression and anxiety; (iii) tolerability

© 2021 Shah AA et al. *JAMA Network Open.*

Pollak et al,[143] 2001	Single center RCT (Austria)	Preterm infants gestational age <31 weeks, Hct >30%, phlebotomy <1 ml/day<br><br>N = 29	Iron sucrose 2mg/kg/day	Comparator 1: Oral iron<br><br>Comparator 2: Oral iron + IV EPO	Unclear	
Ponikowski et al,[145] 2015	Multicenter RCT (n = 41) (Europe)	Age >18 years, Stable NYHA II-III congestive heart failure, LVEF <45%, BNP > 100 pg/ml, able to perform 6-MWT, ferritin < 100 ng/mL or < 300 ng/mL with Tsat < 20%<br><br>n = 304	Ferric carboxymaltose, calculated on Hb and body weight (administered 6-weekly for three doses, then 12 weekly for 36 weeks	0.9% saline IV placebo (opaque infusion kits)	Change in 6-MWT from baseline to week 24	(i) Patient global assessments regularly up to week 52; (ii) changes in NYHA class; (iii) changes in fatigue scores and HRQoL (EQ-5D; (iv) adverse events; (v) hospitalisation rate; (vi) mortality; (v) changes in laboratory markers (Hb, iron status, cardiac biomarkers); (vi) adverse events
Ponikowski et al,[144] 2020	Multicenter RCT (n = 121) (Europe, South America, Singapore)	Age >18 years, hospitalised for acute heart failure with LVEF <50%, evidence of iron deficiency defined as ferritin <100 ng/mL or 100-299 ng/mL with Tsat <20%<br><br>n = 1132	Ferric carboxymaltose 500mg, two doses (repletion and maintenance), subsequent doses based if ID persisted	0.9% saline IV placebo (opaque infusion kits)	Composite of recurrent heart failure hospitalisations and cardiovascular death up to 52 weeks post-randomisation	(i) Composite of recurrent cardiovascular hospitalisations and cardiovascular death; (ii) heart failure hospitalisation; (iii) cardiovascular mortality; (iv) days lost due to heart failure hospitalisations or cardiovascular death; (v) safety
Price et al,[146] 2014	Multicenter RCT (USA)	Age >65 year, Hb 9.0-11.5 g/dL (females), Hb 9.0-12.7 g/dL (males), unexplained anemia,	Iron sucrose 200mg per week for five 5 weeks	No iron	Change in 6-MWT from baseline to 12	(i) Change in Hb; (ii) changes in Geriatric Evaluation Panel

© 2021 Shah AA et al. *JAMA Network Open.*

		serum ferritin between 20 and 200 ng/mL<br><br>n = 19			weeks post-randomisation	
Provenzano et al,[147] 2009	Multicenter RCT (n = 50) (USA)	Adults with CKD requiring haemodialysis for at least 90 days, Hb <11.5 g/dL, Tsat <30%<br><br>n = 230	Ferumoxytol 510mg, two infusions	Ferrous fumarate 50mg daily for 21 days	Change in Hb from baseline to day 35	(i) Proportion of patients achieving Hb >1 g/dL; (ii) changes in iron profiles
Quinibi et al,[148] 2011	Multicenter RCT (n = 47) (USA, Australia)	Age >12 years, non-dialysis dependent CKD, Hb <11 g/dL, ferritin <300 mcg/L, Tsat <25%<br><br>n = 255	Ferric carboxymaltose 1000mg (further 500mg doses on day 17 and day 31 if Tsat <30% and ferritin <500 mcg/L)	Ferrous sulphate 195mg x3/day for 56 days	Percentage of patients with Hb response >1 g/dL	(i) Changes in Hb during the course of the study; (ii) adverse events
Rathod et al,[149] 2015^	Single center RCT (India)	Postpartum anaemia, Hb <10 g/dL<br><br>n = 366	Intervention 1: Iron sucrose, dose calculated for total iron deficit using Ganzoni formula<br><br>Intervention 2: Ferric carboxymaltose 1000mg single dose	Ferrous ascorbate 100mg daily for six weeks	Changes in Hb and ferritin at two and six weeks	Adverse effects
Razzaq et al,[168] 2017	Single center RCT (Pakistan)	Postpartum anaemia, Hb <10 g/dL<br><br>n = 82	Formulation not reported, up to 1000mg weekly	Ferrous sulfate 325mg x3/day for 6 weeks	Proportion of participants with rise in Hb >3.5 g/dL	

Reinisch et al,[150] 2013	Multicenter RCT (n = 36) (Europe and Asia)	Age >18 years with IBD, Hb <12 g/dL, Tsat <20%<br><br>n = 338	Iron isomaltoside, dose calculated according to Ganzoni formula	Iron sulfate 200 m.g. once daily for 8 weeks	Change in Hb from baseline to week 8	(i) Change in Hb at weeks 2 and 4; (ii) changes in ferritin and Tsat at week 8; (iii) HRQoL (IBD-Q); (iv) safety and adverse events
Richards et al,[151] 2020	Multicenter RCT (n = 46) (UK)	Age >18 years scheduled to undergo major abdominal surgery with WHO definition of anaemia (Hb <12 g/dL for females, <13 g/dL for males)<br><br>n = 487	Ferric carboxymaltose 1000mg (between 10 – 42 days before surgery)	0.9% saline IV placebo (opaque infusion kits)	Composite of RBC transfusion or death 30 days after the index operation	(i)) Total number of RBC units transfused; (ii) changes in Hb from randomisation to day of surgery and 8 weeks and 6 months postoperatively; (iii) ICU and hospital LOS; (iv) postoperative complications; (v) readmissions; (vi) HRQoL
Rudra et al,[152] 2016	Single center RCT (India)	Pregnant women 24 to 34 weeks' gestation, Hb 7-9 g/dL<br><br>n = 200	Iron sucrose, dose calculated to achieve target Hb of 11 g/dL	Ferrous ascorbate 100mg x2/day until 6 weeks postpartum	Efficacy, safety and acceptability of iron sucrose	
Santer et al,[153] 2020	Single center RCT (UK)	Age >18 years, COPD with no exacerbation in previous 4 weeks<br><br>n = 48	Ferric carboxymaltose 1000mg	0.9% saline IV placebo (opaque infusion kits)	Change in resting peripheral oxygen saturations from baseline to 1 week after infusion	(i) Changes from baseline to follow-up visits in oxygenation; (ii) 6-MWT; (iii) laboratory parameters (Hb, CRP, iron profiles); (iv) COPD exacerbations; (v) HRQoL (MRC Dyspnoea, Fatigue Severity); (vii) echocardiography changes

© 2021 Shah AA et al. *JAMA Network Open.*

Schijns et al,[154] 2020	Multicenter RCT (n = 3) (Netherlands)	Women aged 18-65 years, preoperative ferritin >20 mcg/L and then developed iron deficiency post-gastric bypass<br><br>n = 120	Ferric carboxymaltose 1000mg	Comparator 1: Ferrous fumarate 200mg x3/day<br><br>Comparator: Ferrous gluconate 695mg x2/day	Percentage of patients with ferritin >20 mcg/L at 3- and 12-months following treatment	
Schroder et al,[155] 2005	Multicenter RCT (Germany)	Adult patients aged 18-85 years with IBD, iron deficiency anaemia defined as Hb <10.5 g/dL (females) or <11 g/dL (males) and Tsat <20% or ferritin <20 mcg/L<br><br>n = 46	Iron sucrose 7 mg/kg loading dose, followed by 200mg weekly for 5 weeks	Iron sulfate 100-200mg daily for 6 weeks	Maximal change in Hb from baseline until 6 weeks	(i) Adverse events; (ii) changes in HRQoL; (iii) changes in Tsat or ferritin
Sedighi et al,[156] 2013	Multicenter RCT (n = 3) (Iran)	Age >18 years, CKD established on haemodialysis for more than 3 months, Hb <11 g/dL, ferritin >500 ng/mL, Tsat <25%<br><br>n = 40	Iron sucrose 100mg twice weekly for 5 weeks	Ascorbic Acid for 12 weeks	Change in Hb from baseline to week 12	Changes in ferritin, serum iron, TIBC and Tsat
Seid et al,[158] 2008	Multicenter RCT (n = 28) (USA)	Postpartum anaemia (Hb <10 g/dL) within 10 days of delivery<br><br>n = 291	Ferric carboxymaltose, dose calculated according to Ganzoni formula	Ferrous sulphate 65mg x3/day for 6 weeks	Percentage of participants with Hb >12g/dL between baseline and end of study	(i) Percentage of participants with Hb rise >3 g/dL; (ii) time taken to achieve Hb rise >3 g/dL; (iii) changes in Hb, ferritin, and Tsat; (iv) Safety
Seid et al,[157] 2017	Multicenter RCT (n = 130)	Heavy menstrual bleeding or post-partum anaemia, Hb <11	Ferric carboxymaltose	Usual medical care	Safety (Incidence of AEs, death, hospitalisation, disability, congenital	Changes in Hb and ferritin from baseline

© 2021 Shah AA et al. *JAMA Network Open.*

		g/dL, iron deficiency (definition not provided)<br><br>n = 2045	15mg/kg once only		anomaly/birth defect, life-threatening events)	to highest post-randomisation value
Serrano-Trenas et al,[159] 2011	Single centre RCT (Spain)	Age >65 years, scheduled to undergo hip fracture surgery<br><br>n = 200	Iron sucrose 600mg - given in 200mg every 48 hours (first dose pre-operatively)	Usual medical care	RBC transfusion requirements	(i) Changes in iron profiles; (ii) hospital length of stay; (iii) infection; (iv) 30-day mortality; (v) side-effects
Singh et al,[161] 1998	Single center RCT (Singapore)	Pregnant women age >16 years, 20-28 weeks' gestation, Hb <9 g/dL, ferritin <20 mg/L<br><br>n = 100	Iron polymaltose, dose calculated to Hb deficit	Iron fumarate 200mg x3/day for 12 weeks	Changes in Hb at 36 weeks, delivery and 6 weeks postpartum	Changes in iron profiles
Singh et al,[160] 2006	Multicenter RCT (n = 21) (USA & Mexico)	Age >18 years, CKD on peritoneal dialysis, Hb 9.5-11.5 g/dL, established on EPO, Tsat <25%, ferritin <500 ng/ml<br><br>n = 121	Iron sucrose 1000mg, divided over a 28-day period	No iron	Change from baseline to highest Hb during study period	(i) Changes in Tsat and ferritin; (ii) time to anaemia intervention; (iii) safety events
Sloand et al,[162] 2004	Single center RCT (USA)	CKD (end stage) and Restless leg syndrome<br><br>n = 25	Iron dextran 1000mg (over multiple doses)	0.9% saline IV placebo (opaque infusion kits)	Changes in International RLS score from baseline to 4 weeks post-infusion	Adverse events
Spahn et al,[163] 2019	Single center RCT (Switzerland)	Age >18 years, undergoing CABG and/or valve surgery, Hb <130 g/L (males) or <120 g/L (females) or ferritin <100 mcg/L<br><br>n = 1006	Ferric carboxymaltose 1000mg once only and Eprex 40,000 units (preoperatively)<br><br>B12 and folate also supplemented	0.9% saline IV placebo (opaque infusion kits)	Numbers of RBCs transfused within 7 days	(i) Percentage with any RBC transfusion in 90 days; (ii) duration of mechanical ventilation; (iii) acute kidney injury; (iv) infection (7 days); (v) VTE (90 days); (vi) 30 and 90-day mortality; (vii) hospital LOS; (viii) changes in peri-

© 2021 Shah AA et al. *JAMA Network Open.*

						operative Hb concentrations
Spinowitz et al,[164] 2008	Multicenter RCT (n = 13) (USA)	Age >18 years, CKD stages 1-5, Hb <11 g/dL<br><br>n = 304	Ferumoxytol 510mg – two doses	Ferrous fumarate 200mg daily for 21 days	Changes in Hb from baseline to day 35	(i) Changes in iron profiles; (ii) proportion of patients increasing Hb by >1 g/dL
Steensma et al,[165] 2011	Multicenter RCT (USA)	Age >18 years, receiving chemotherapy for non-myeloid neoplasm, Hb <11 g/dL, ferritin >20 mcg/L, Tsat <60%<br><br>n = 502	Ferric gluconate 187.mg every 3 weeks (for 5 doses)	Comparator 1: Ferrous sulphate 325mg for 16 weeks<br><br>Comparator 2: Oral placebo for 16 weeks	Proportion of patients with Hb response >2 g/dL from baseline	(i) Transfusion requirements; (ii) ESA dose; (iii) changes in HRQoL; (iv) adverse events
Stoves et al,[166] 2001	Single center RCT (UK)	Adults with progressive CKD and worsening anaemia Hb <11 g/dL<br><br>n = 45	Iron sucrose 300mg - repeated monthly according to serum ferritin	Ferrous sulphate 200mg x3/day	Changes in ferritin and EPO dose requirements<br><br>Average follow-up: 5.2 months	
Toblli et al,[168] 2007	Single center RCT (Argentina)	Adults with LVEF <35%, anaemia (Hb <12.5 g/dL for men, <11.5 g/dL for women) with ferritin <100 ng/mL and Tsat <20%<br><br>n = 40	Iron sucrose 200mg weekly for 5 weeks	0.9% saline IV placebo (opaque infusion kits)	Improvement in Hb and renal function	(i) Changes in BNP and inflammation; (ii) no. of hospitalisations; (iii) changes in exercise tolerance and changes in HRQoL
Trenkwalder et al,[169] 2017	Multicenter RCT (n = 13) (Finland, Germany, Switzerland)	Adults with restless leg syndrome, International RLS >15, Hb <11.5 g/dL (females); <12.5 g/dL (males)<br><br>n = 110	Ferric carboxymaltose 1000mg	0.9% saline IV placebo (opaque infusion kits)	Mean change in International RLS score from baseline to week 4 post-randomisation	(i) Changes in IRLS score from baseline to week 12; (ii) changes in ferritin, Tsat; (iii) Adverse events

© 2021 Shah AA et al. *JAMA Network Open.*

Vadhan-Raj et al,[170] 2013	Multicenter RCT (n = 192) (Europe, Asia, North America)	Anaemia (Hb 7-10 g/dL) secondary to any underlying condition (gastric bleed, cancer, postpartum anaemia, heart failure, rheumatoid arthritis)<br><br>n = 812	Ferumoxytol 510mg - two doses	0.9% saline IV placebo (opaque infusion kits)	Proportion of patients achieving Hb rise of >2 g/dL at any point from baseline to week 5	(i) Proportion of patients with Hb >12 g/dL; (ii) changes in iron profiles; (iii) changes in fatigue scales and SF-36; (iv) safety
Van Iperen et al,[171] 2000	Single center RCT (Netherlands)	Age >18 years, expected ICU length of stay >7 days, Hb <11.2 g/dL (or 12.1 g/dL if ischaemic heart disease)<br><br>n = 36	Iron sucrose 20mg daily from day 1-14	No iron	Changes in Hb and reticulocyte counts at 14 days	(i) Changes in EPO and iron profiles; (ii) blood loss; (iii) RBC transfusion requirements; (iv) ICU length of stay; (v) mortality
van Veldhuisen et al,[172] 2017	Single center RCT (n = 28) (Europe)	Age >18 years, clinically stable heart failure NYHA II to III, LVEF <45%, Ferritin <100 ng/ml or <300 ng/ml with Tsat <20%<br><br>n = 172	Ferric carboxymaltose, administered at week 0 and week 6, based on screening Hb and weight	Usual medical care	Change in peak oxygen consumption from baseline to week 24	(i) Effect on haematinic and cardiac biomarkers; (ii) HRQoL; (iii) safety
Van Wyck et al,[175] 2005	Multicenter RCT (n = 35) (USA)	Adults, Stage 3-5 CKD, Hb <11 g/dL, Tsat <25%, ferritin <300 ng/mL<br><br>n = 188	Iron sucrose 1000mg in divided doses over 14 days	Ferrous sulphate 195mg x3/day for 56 days	Proportion of patients with an Hb increase of 1 g/dL	Changes in Hb and iron profiles
Van Wyck et al,[174] 2007	Multicenter RCT (n = 43) (USA and Mexico)	Postpartum anaemia, Hb <10 g/dL within 10 days of delivery<br><br>n = 361	Ferric carboxymaltose, dose calculated to target Hb	Ferrous sulphate 65mg x3/day for 42 days	Change in Hb by > 2g/dL after treatment	(i) Proportion of patients with Hb >12 g/dL; (ii) time to achieve to primary outcome; (iii) changes in iron profiles; (iv) transfusion requirements; (v) HRQoL

© 2021 Shah AA et al. *JAMA Network Open.*

Van Wyck et al,[173] 2009	Multicenter RCT (n = 79) (USA and Mexico)	Adult females age >18 years, Hb <11 g/dL due to heavy uterine bleeding, Tsat <25%, ferritin <100 ng/mL<br><br>n = 477	Ferric carboxymaltose, dose calculated to target Hb	Ferrous sulphate 65mg x3/day for 42 days	Proportion of patients with Hb increase >2 g/dL	i) Proportion of patients with Hb >12 g/dL; (ii) time to achieve to primary outcome; (iii) changes in iron profiles; (iv) RBC transfusion requirements; (v) HRQoL
Vanobberghen et al,[176] 2020	Multicenter RCT (n = 2) (Tanzania)	Pregnant women within 14 days of delivery with IDA (Hb <11 g/dL, ferritin <50 mcg/L)<br><br>n = 230	Ferric carboxymaltose, doses based on weight and Hb	Ferrous sulphate 60mg once daily for 6 weeks	Proportion of patients with normalisation of Hb (>11.5 g/dL)	(i) Proportion of patients with corrected ID (raised serum ferritin >100 mcg/L); (ii) adverse events; (iii) SF-36; (iv) adherence to study medication
Verma et al,[184] 2011	Single center RCT (India)	Postpartum anaemia Hb <8 g/dL, within 24 hours of delivery<br><br>n = 150	Iron sucrose 200mg every 48 hours (3 doses)	Oral iron 200 mg ferrous sulphate x2/day for one month	Changes in Hb for the first 4 weeks postpartum	
Warady et al,[177] 2004	Multicenter RCT (n = 5) (USA)	Paediatric patients with ESRD (age 1-20 years), haemodialysis for >2 months, Tsat >20%<br><br>n = 35	Iron dextran, (weight based) weekly for 6 weeks	Ferrous fumarate 4-6 mg/kg/day for 6 weeks	Maintenance of target Hct between 33% and 36%, reduction of EPO dose and prevention of iron deficiency	(i) Changes in iron status; (ii) parathyroid hormone levels and blood urea nitrogen levels during the study
Weisbach et al,[178] 1999	Single center RCT (Germany)	Age >18 years, scheduled for autologous blood donation (minimum of 3 units) prior to major orthopaedic or cardiovascular surgery<br><br>n = 123	Iron sucrose 200mg after each donation	Comparator 1: Ferrous fumarate 100mg x3/day up to day before surgery<br><br>Comparator 2: No iron	Net RBC production during the donation period	(i) Changes in Hb values and iron profiles; (ii) postoperative erythropoiesis; (iii) adverse events

Westad et al,[179] 2008	Multicenter RCT (n = 5) (Norway)	Postpartum anaemia, Hb 6.5-8.5 g/dL within 48 hours after delivery<br><br>n = 129	Iron sucrose 600mg (split over 3 doses)	Ferrous sulphate 100 mg x2/day from inclusion until 12 weeks	Change in Hb at 4 weeks postpartum	(i) Changes in Hb and iron profiles at 8 and 12 weeks postpartum; (ii) HRQoL (SF-36) after four, eight and 12 weeks
Woods et al,[180] 2014	Single center RCT (Australia)	Elite runners with non-anaemic iron deficiency (Hb >12 g/dL, ferritin 30-100 mcg/L)<br><br>n = 14	Ferric carboxymaltose 100mg twice a week for 4 weeks	0.9% saline IV placebo (opaque infusion kits)	Changes in fatigue (Brief Fatigue Inventory) and mood (Brunel Mood Scale)	(i) Changes in exercise performance; (ii) changes in Hb and iron profiles
Xu et al,[181] 2020	Single center RCT (China)	Age 20-70 years, postoperative anaemia following cardiac surgery WHO criteria (male, Hb <13 g/dL; female, Hb <12 g/dL), ferritin 30-100 mcg/L or Tsat <20%<br><br>n = 150	Iron sucrose 200mg on alternate days until calculated total iron deficit met, started day after surgery on cardiac ICU	0.9% saline IV placebo (opaque infusion kits)	Changes in Hb on postoperative day 7 and 14	(i) Changes in iron profiles; (ii) clinical – infection, length of ventilation, ICU and hospital stay; (iii) adverse events
Yeo et al,[182] 2018	Multicenter RCT (n = 2) (Singapore)	Adults admitted with decompensated heart failure, ferritin <100 ng/ml, Tsat <20%, Hb <14 g/dL<br><br>n = 50	Ferric carboxymaltose 1000mg	0.9% saline (unblinded)	Change in 6-MWT from baseline to 12 weeks	(i) Changes in NYHA class and Kansas City Cardiomyopathy Questionnaire (KCCQ) (ii) hospital admission for heart failure; (iii) serious adverse events
Yoo et al,[183] 2011	Single center RCT (South Korea)	Adults scheduled for valvular heart surgery, preoperative anaemia according WHO definition<br><br>n = 74	Iron sucrose 200mg and EPO (500 IU/kg) 16-24 prior to surgery	0.9% saline IV placebo (opaque infusion kits)	RBC transfusion requirements during surgery and for first 4 postoperative days	(i) Changes in Hb and iron profiles; (ii) postoperative complications

Abbreviations: 6-MWT, six minute walk test; BI, Barthel's index; CRP, C-reactive protein; COPD, Chronic Obstructive Pulmonary Disease; DVT, deep vein thrombosis; CKD, chronic kidney disease; EPO, erythropoietin; ESA, erythropoiesis-stimulating agent; ESR, Erythrocyte sedimentation rate; ESRD, End-stage renal disease; FIQR, fibromyalgia impact questionnaire; Hb, haemoglobin; HD, haemodialysis; HRQoL, health-related quality of life; IBD, inflammatory bowel disease; ICU, intensive care unit;

© 2021 Shah AA et al. *JAMA Network Open.*

IV, intravenous; LOS, length of stay; LVEF, left ventricular ejection fraction; MFI, Multidimensional fatigue inventory; MI, myocardial infarction; s.c., subcutaneous; NYHA, New York Heart Association; PFS, Piper Fatigue Scale; RBC, red blood cell; RLS, restless leg syndrome; SF-36, Short-Form 36; Tsat, transferrin saturation; VTE, venous thromboembolism

*Trial analysed as two separate studies
^ Data for both IV iron groups were combined
# This trial had an oral iron run-in period with two parallel randomisations after this based on tolerance of oral therapy -- in the results we have only included the participants in the randomisation of oral-tolerant participants (FCM vs oral iron) and not the other randomisation (FCM vs other IV standard of care)

© 2021 Shah AA et al. *JAMA Network Open.*

eTable 2. Characteristics of ongoing RCTs

Study details and status	Sample size	Participants	Intervention(s)			Primary outcome	Secondary outcome	Infection reporting
			Arm 1	Arm 2	Arm 3			
Surgery								
NCT03817957; IDA-II; Germany (Recruiting)	407	Age >18 years; following major surgery (orthopedic/trauma vascular, visceral, cardiac), Hb <12 g/dL for females and <13 g/dL for males or non-anemic ID (ferritin <100 mcg/L, confirmed 28 days before surgery)	Ferric carboxymaltose 1000mg	Polyglucoferron 500-2000 mg IV	Ferrous sulphate 50-200mg x2/day for 28 days	Proportion of patients who achieve normal Hb or Hb rise >1.5 g/dL at 30 days: pre- and post-volume-corrected urine iron levels	Changes in Hb and iron profiles; adverse events and tolerability; all-cause mortality; changes in SF-36	Not specified
NCT02972294; HiFIT; France (Recruiting)	780	Age >18 years, NOF fracture; Hb 9.5-13 g/dL	Iron isomaltoside 1000mg + tranexamic acid	Iron isomaltoside + IV placebo	IV placebo + tranexamic acid<br><br>Arm 4: IV placebo x2	Peri-operative transfusion requirements	No. of blood products per patients; changes in Hb up to day 30 post-op; peri-operative blood loss; proportion of patients home at day 30; all-cause mortality; postoperative complications (cardiovascular, infection, transfusion-related, anaphylaxis); QoL (EQ-5D-5L, PQOL, IADL);	Yes – 'infectious complications'

© 2021 Shah AA et al. *JAMA Network Open.*

							all-cause mortality	
KCT0004020; South Korea; (Recruiting)	80	Age >60 years, scheduled to undergo total shoulder arthroplasty; post-op Hb <10 g/dL or fall greater than 3 g/dL from pre-operative value.	Ferric carboxymaltose 1000mg	IV placebo (0.9% saline)		Changes in Hb up to 3 months postoperatively	Changes in ferritin, iron, total iron binding capacity, Tsat	Not specified
NCT04083755 IRONPAD; Spain (Recruiting)	230	Age >18 years; Hb <130 g/L (males); Hb <120 g/L (females), symptomatic lower limb ischaemia; >1 week prior to surgery	Ferric carboxymaltose 1000mg	No treatment or oral iron		Transfusion requirements	Changes in Hb; length of stay; hospital mortality; adverse events; SF-36 at day 30 post-op	Not specified
NCT03528564; HOPE-Hb; Canada (Recruiting)	74	Age >18 years, scheduled to undergo primary total hip or knee arthroplasty; Hb <12 g/dL	Iron sucrose 900mg + EPO 40000 IU	Iron sucrose 900mg + SC placebo		Feasibility and pre-operative Hb on day of surgery	Postoperative Hb; changes in Hb and iron profiles; transfusion requirements; DVT; postoperative morbidity; surgical wound infection; HADS; cognitive assessments; cost analysis	Yes – part of composite of postoperative morbidity up to 3 months post-surgery and surgical wound infection (superficial and deep) 6 weeks from surgery
EudraCT 2018-004213-41; RIPAC; Belgium (Recruiting)	60	Age >18 years, scheduled to undergo colonic cancer resection; Hb <12 g/dL in women and <13	Ferric carboxymaltose 1000mg	Usual care		Change in Hb at time of surgery	Peri-operative transfusion requirements; change in Hb at 1 month postoperative;	Yes – as part of postoperative morbidity assessment using Clavien-

© 2021 Shah AA et al. *JAMA Network Open.*

		g/dL in men; Tsat <20%					postoperative complications; length of stay; QoL (EORTC QLQ-C30)	Dindo classification
NCT03565354; Hong Kong (Completed – results awaited)	40	Age >18 years, scheduled to undergo colorectal cancer surgery; Hb <13 g/dL, ferritin <30 mcg/L or ferritin 100-300 mcg/L with Tsat <20%	Iron isomaltoside 1000mg	Usual care		Changes in pre-operative Hb and ferritin	Peri-operative transfusion requirements; hospital length of stay, quality of recovery; surgical complication; DAOH-30	Yes – as part of postoperative morbidity assessment using Clavien-Dindo classification
NCT03574311; PREFER-CABG; Finland (Recruiting)	210	Age >18 years; open heart surgery	Ferric carboxymaltose 1000mg pre-operatively	IV placebo (0.9% saline)		Composite of incidence of RBC transfusion and/or nosocomial infection	Mortality at 90-days; length of stay; peri-operative MI; organ support requirements; HRQoL at one year	Yes – part of composite of primary outcome (incidence of RBC transfusion and/or nosocomial infection)
ACTRN12618001437257; ADEPT; Australia (Recruiting)	40	Scheduled to undergo elective open/laparoscopic abdominal surgery; Non-anemic iron deficiency	Ferric carboxymaltose 1000mg pre-operatively	IV placebo (0.9% saline)		Changes in peak VO_2 measured by CPEX	Changes in AT; changes in Hb; changes in EQ-5D-5L and WHODAS V2.0 (30 days post-operatively); peri-operative transfusion requirements; post-operative complications (including infection); DAOH-90	Yes – part of postoperative complication assessment

© 2021 Shah AA et al. *JAMA Network Open.*

ICTRP EUCTR2018-002571-18-NL; EFFECT KTx; Netherlands (Recruiting)	158	Age >18 years; Kidney transplant recipients (at least 4 months after transplant); ID (defined as ferritin < 100µg/L or ferritin between 100 and 300 µg/L in combination with a transferrin saturation < 20%);	Ferric carboxymaltose 1000mg	IV placebo (0.9% saline)		Changes in 6MWT at 24 weeks post-intervention	Changes in Hb and iron profiles; changes in LVEF; HRQoL (SF-36, EQ-5D, KDQOL-SF); infection; cardiovascular events; graft rejection; mortality	Yes – defined as 'occurrence of infectious diseases'
NCT03662789; IronIC; Norway (Complete – results awaited)	102	Age >18 years, presentation at least one year post-cardiac transplant; ID (ID (defined at Tsat <20% with ferritin 100-300 µg/L or ferritin <100 µg/L)	Iron isomaltoside 20mg/kg	IV placebo (0.9% saline)		Peak oxygen consumption at 6 months	Changes in iron profiles; muscle strength (hand-grip dynamometer); changes in HRQoL (SF-36); changes in laboratory CRP, troponin and NT-proBNP	Not specified
NCT04268849; USA (Recruiting)	104	Age >18 years, undergone bariatric surgery >3 months ago, IDA (ferritin<30 mcg/L, Tsat <20%, Hb <13 g/dL)	Ferumoxytol 1020mg + oral placebo	Ferrous sulphate 60mg and vitamin C for 6 weeks + IV placebo (0.9% saline)		Changes in Clinical Global Impression at 6 weeks post-treatment; Changes in Hb	Changes in ferritin and Tsat; QoL measured by visual linear analogue scale	Not specified
**Oncology**								
ICTRP KCT0004311; South Korea (Recruiting)	341	Adults >19 years, solid cancer or lymphoma, Hb 8.0-10.5 g/dL, recent chemotherapy	Ferric carboxymaltose 1000mg	Usual care		Change in Hb	Adverse events, quality of life with EORTC QLQ-C30	Not specified

© 2021 Shah AA et al. *JAMA Network Open.*

ISRCTN13370767 ICARAS; UK (Recruiting)	40	Age ≥ 18 years; Solid tumours not amenable to curative treatment; Hb <130 g/L (males) / <120 g/L (females)' moderate-severe fatigue	Iron isomaltoside (20 mg/kg)	IV placebo (0.9% saline)		Feasibility (recruitment, randomisation, protocol adherence, retention)	Quality of life (FACIT-F, EQ-5D, EORTC QOQ-C30 and baseline, 4 and 8 weeks post-infusion); transfusion requirements; changes in Hb, iron profiles, cytokine activity; gut microbiome; daily steps	Not specified
Eudra CT 2014-000246-30; FERINJECT; Germany (Complete – results awaited)	64	Age >18 years; inoperable colorectal cancer; iron deficiency anaemia: Hb ≤ 10.5 g/dL and transferrin saturation < 20 % and/or serum ferritin < 20 ng/mL	Ferric carboxymaltose 1000mg	Ferrous sulphate 100mg		Rise in Hb by 2 g/dL at 12 weeks	QoL (EORTC-QLQ-C30), hand grip via dynamometer; transfusion requirements; changes in iron profiles; adverse events; overall survival	Not specified
NCT04206228 IIISAS; Norway (Recruiting)	100	Age>18 years, Aortic stenosis and scheduled for TAVI, ID (ferritin < 100 µg/L or ferritin between 100 and 300 µg/L in combination with a transferrin saturation < 20%)	Iron isomaltoside (20mg/kg)	IV placebo (0.9% saline)		Baseline-adjusted 6-minute walk test (6 months after intervention)	Changes in iron status, NT-proBNP, and Troponin; muscle strength; SF-36; EQ-VAS, HADS, Kansas City Cardiomyopathy Questionnaire; adverse events	Not specified
**Obstetrics / Women's health**								
NCT03188445; Denmark (Recruiting)	200	Women aged >18 years; Pregnant >14 weeks gestation;	Iron isomaltoside 1000mg	Ferrous fumarate + ascorbic acid		Time to achieve Hb >11 g/dL	Changes in Hb and iron biomarkers	Not specified

© 2021 Shah AA et al. *JAMA Network Open.*

		Ferritin <30 µg/L after 4 weeks of oral iron				(baseline to 18 weeks after treatment)		
ACTRN12619000283178p; IRONWOMAN; Australia (Not yet open)	50	Pregnant women aged >18 years; Hb 80-105 g/dL and ferritin <30 µg/L; between 26-33 weeks' gestation	Ferric carboxymaltose 1000mg + oral placebo capsules	Oral iron 80 mg once daily + IV placebo		Feasibility – adequacy of blinding	Patient and clinician acceptability; changes in SF-36 from baseline to 4 weeks; treatment side effects; oral iron compliance; proportion of women with Hb <105 g/dL at 4 weeks; changes in Hb and iron profiles; maternal outcomes (PPH, mode of delivery, transfusion requirements); fetal outcomes (SGA, birth weight, preterm labour, death, NICU admission)	Not specified
ACTRN12618001268235; REVAMP; Malawi (Recruiting)	862	Pregnant women 13-26 weeks' gestation; Hb <10 g/dL; negative malaria test	Ferric carboxymaltose 1000mg	Ferrous sulphate 200mg x2/day for 90 days		Prevalence of Hb <10 g/dL at timepoint closest to delivery	Maternal fatigue (1 month postpartum); changes in Hb and iron profiles; incidence of PPH, length of stay post-delivery; maternal cognitive function; transfusion requirements;	Yes – incidence of malaria and /or parasitaemia

© 2021 Shah AA et al. *JAMA Network Open.*

							adverse events; neonatal outcomes (birth weight, preterm birth, still birth)	
NCT04505514; IVIronPPH; Malaysia (Recruiting)	60	Age >18 years; PPH >500 mls; Hb <10 g/dL	Iron isomaltoside 1000mg + oral iron preparation (Iberet-Folic 500)	IV placebo + oral iron preparation (Iberet-Folic 500)		Changes in Hb, serum iron and ferritin at 6 weeks	General fatigue score; adverse events; transfusion requirements	No
NCT04205266; USA (Not yet recruiting)	76	Women aged 18-50; heavy menstrual bleeding; Hb <11 g/dL	Ferumoxytol 510 mg (2 infusion)	Ferrous sulphate 325mg once daily for 60 days		Changes in Hb at 60 points	Patient satisfaction with treatment; quality of life score	No
NCT03957057; Slovenia (Recruiting)	300	PPH with Hb 7-10 g/dL within 48 hours after delivery	Ferric carboxymaltose 1000mg	Iron isomaltoside 1500mg	Ferrous sulphate 160 mg once daily for 6 weeks	MFI score at 6 weeks postpartum	Edinburgh Postnatal Depression score; changes in Hb and iron profiles; side effects; compliance with oral iron	Yes – part of composite of adverse events listed as 'upper respiratory tract infection'
CTRI/2020/02/023125; India (Recruiting)	80	Women age 18-45 years, postpartum Hb 8-10 g/dL	Ferric carboxymaltose 1000mg	Ferrous fumarate 152mg x2/day for 6 weeks		Changes in Hb at 2 and 6 weeks postpartum	Safety and acceptability	No
**Cardiology / heart failure**								
ISRCTN16403302; IRONMAN; UK (Recruiting)	1300	Age >18 years, LVEF <45%; NYHA class II-IV; ID (defined at Tsat <20% with ferritin 100-300 µg/L or ferritin <100 µg/L)	Iron isomaltoside (calculated to body weight)	Usual care		Cardiovascular mortality or hospitalisation for heart failure throughout follow-up period (approx. 3 years)	All-cause mortality; all-cause hospitalisation; HRQoL (Minnesota Living with Heart Failure; EQ-5D) at 4 and 20	Yes - Hospitalisation primarily for infection is assessed through review of case notes and reporting of patient, relative,

© 2021 Shah AA et al. *JAMA Network Open.*

							months; QALY at 2.5 years; Death / hospitalisation primarily due to infection; 6MWT	carer or clinician throughout the follow up period
EudraCT 2019-002912-25; HEART-FID; USA (Recruiting)	1508	Age >18 years, reduced LVEF; ID ((defined at Tsat <20% and/or ferritin <100 µg/L); Hb between 9 g/dL and 13.5 g/dL, able to perform 6-MWT	Ferric carboxymaltose 1000mg	IV placebo (0.9% saline)		Composite of death, hospitalisation for heart failure (1 year) or change in 6-MWT (6 months)	Time to first composite event; cardiovascular death; time to cardiovascular death; cardiovascular hospitalization; urgent heart failure visits	No
NCT03833336; PREFER-HF; Spain (Recruiting)	72	Stable, chronic HF; LVEF >45%; ID (defined at Tsat <20% with ferritin 100-300 µg/L or ferritin <100 µg/L), able to perform 6-MWT	Ferric carboxymaltose 500-1000 mg at 0,6,12 and 24 weeks	Oral iron: Ferrous sulphate 100mg b.d. until week 24<br>OR<br>Sucrosomial iron 30mg until week 24	IV and oral placebo	Changes in 6-MWT at 24 weeks	Changes in NYHA; hospitalisation; mortality; QoL (Kansas City Cardiomyopathy Questionnaire) – all at 24 weeks	No
ACTRN126200000285954; IRON-AF; Australia (Recruiting)	84	Age >18 years, persistent or paroxysmal AF, Hb <15 g/dL, ID (defined at Tsat <20% with ferritin 100-300 µg/L or ferritin <100 µg/L)	Ferric carboxymaltose 1000mg at weeks 0,1,4 and 8	IV placebo (0.9% saline)		Changes in VO_2 at 12 weeks	Changes in QoL (SF-36, AF Effect of QoL, Patient Global Assessment, AF severity scale); changes in 6-MWT; changes in NYHA and echocardiography; mortality; hospitalisations	No

© 2021 Shah AA et al. *JAMA Network Open.*

NCT04291690; TARGET-EFT; Canada (Recruiting)	144	Age >65 years, frail or pre-frail defined by EFT score >1, admission to cardiovascular unit	Multi-component- IV iron (Iron sucrose 300mg daily for 3 doses) if presence of IDA (Hb<13 g/dL, ferritin <100 µg/L or ferritin <300 µg/L and Tsat <20%) physical training; cognitive stimulation; nutritional support	Usual care		Changes in EQ-5D-5L from randomisation to 6 weeks	Changes in hospital-acquired disability; all-cause mortality; delirium; length of stay, mobility, step counts; HADS	Yes – part of composite of adverse events assessed up to 6 weeks post treatment
**Paediatrics**								
NCT03523117; USA (Recruiting)	72	Age 1-17 years; Hb <11 g/dL; ferritin <300 ng/mL, Tsat <30%, inadequate response to oral iron	Ferric carboxymaltose 15mg/kg (max 750 mg) at day 0 and day 7	Ferrous sulphate dose adjusted for age, for 28 days		Changes in Hb at day 35	Changes in ferritin, Tsat and Retic Hb	No
ChiCTR18000019815; China (Recruiting)	268	Children aged <14 years, scheduled to undergo major abdominal surgery; ferritin <15 mcg/L, Tsat <15%	I.v iron infusion (no further details)	Usual care		Incidence of RBC transfusion		No
**Chronic Kidney Disease**								
NCT04464850; IVO-IRON Thailand (Recruiting)	124	Age >18 years; Haemodialysis for at least 3 months; Hb 8-11.5 g/dL; Tsat <50% and ferritin <800 mg/dL	Iron sucrose 200mg every 2 weeks for 24 weeks	Ferrous fumarate 600 mg/day for 24 weeks		Change / reduction in EPO dose at 24 weeks	Changes in Hb; MACE; hospitalisations due to infection; Changes in HRQoL	Yes – secondary outcome of hospitalizations due to infection

© 2021 Shah AA et al. *JAMA Network Open.*

							(KDQOL, EQ-5D-5L), cost-effectiveness of oral	
**Critical care**								
NCT02276690; HEPCIDANE France (Completed – results awaited)	408	Age >18 years, required ICU care for at least 5 days, anaemia as per WHO definition	Intravenous iron +/- EPO according ferritin and hepcidin levels	Usual care		Hospital costs	Hospital and ICU LOS; fatigue; changes in Hb and iron profiles; 90-day mortality	No
ISRCTN13721808; INTACT UK (Completed – results awaited)	130	Age >18 years, required ICU care for at least 24 hours, Hb <100 g/L prior to hospital discharge	Ferric carboxymaltose 1000mg	Usual care		Feasibility – recruitment, randomisation and follow-up rates	Changes in Hb and iron profiles; nosocomial infection, in-hospital mortality; changes in fatigue and HRQoL up to 90 days post-randomisation; readmissions	Yes – secondary outcome defined as ‘commencement of new antibiotic therapy or escalation from prophylactic antibiotics for a confirmed or strongly suspected new infection.

Abbreviations: 6-MWT, 6-minute walk test; CPEX, Cardiopulmonary Exercise Testing; CRP, C-reactive protein; DAOH, Days Alive and Out of Hospital; DVT, Deep Vein Thrombosis; EFT, Essential Frailty Toolset; EORTC, European Organisation for Research and Treatment of Cancer; EPO, erythropoietin; EQ-5D-5L, Euroqol-5D-5L; EQ-VAS, EuroQol Visual Analogue Scale; Hb, HADS, Hospital Anxiety and Depression Scale; Hemoglobin; HRQoL, Health-related quality of life; IADL, Instrumental Activities of Daily Living; ICU, Intensive Care Unit; ID, Iron deficiency; IDA, Iron deficiency anemia; IV, intravenous; KDQOL, Kidney Disease Quality of Life Instrument; LOS, Length of stay; LVEF, left ventricular ejection fraction; MACE, Major adverse cardiovascular events; MFI; Multidimensional Fatigue Inventory; MI, myocardial infarction; NICU, Neonatal Intensive Care Unit; NOF, Neck of femur; NYHA, New York Heart Association; PPH, Postpartum Hemorrhage; PQOL, Perceived Quality of Life; QALY, Quality adjusted life year; RBC, red blood cell; SF-36, Short Form-26; TAVI, Transcatheter Aortic Valve Implantation; Tsat, Transferrin saturation; WHO, World Health Organization; WHODAS, WHO Disability Assessment Schedule.

© 2021 Shah AA et al. *JAMA Network Open.*

eTable 3. Characteristics of included NRS

Study ID	Study design	Participants	Exposure	Comparator	Key findings
Ikuta et al,[183] 2019	Prospective cohort study<br><br>Multicenter (n = 12) (Japan)	Iron deficiency anaemia (Hb <12 g/dL in women and <13 g/dL in men, ferritin <12 ng/mL) secondary to digestive diseases<br><br>n = 39	Ferric carboxymaltose 1000mg or 1500mg depending on weight	N/A	10/39 (25.6%) experienced an ‘infection or infestation’<br><br>MedDRA coding definition
Ishida et al,[184] 2015	Retrospective observational registry-based cohort<br><br>Multicenter (USA)	CKD patients who had received IV iron in the 14 days preceding their first hospitalisation<br><br>n = 2463	Receipt of IV iron of any dose, frequency, or type	No IV iron received	IV iron was not associated with hospital admission for infection or death within 30 days (OR 1.08; 95% CI: 0.96 to 1.22)<br><br>ICD-9 Classification used to define infection
Kim et al,[185] 2018	Retrospective propensity-matched cohort study<br><br>Single center (South Korea)	Elective hip arthroplasty and hip fracture with postoperative anaemia (Hb <10 g/dL)<br><br>n = 300	Ferric carboxymaltose 1000mg	Usual medical care	No statistically significant differences in postoperative infections between those who received iron compared with usual care (7/150 (4%) vs. 6/150 (3%), p>0.05)<br><br>Investigator defined – ‘positive bacterial culture and the need for antibiotics’
Kuragano et al,[186] 2014	Prospective observational study<br><br>Single center (Japan)	Stable CKD on maintenance dialysis for a period of 1 year<br><br>n = 1086	IV iron, high dose (>50mg per week) or low dose (<50mg per week)	No iron	IV iron (both cohorts) associated with increased risk of infection when compared with no iron (HR 1.78; 95% CI: 2.24 to 12.14). High ferritin levels also associated with increased risk of infection when compared with low ferritin (HR 1.76; 95% CI: 1.29 to 2.4)<br><br>No definition provided

© 2021 Shah AA et al. *JAMA Network Open.*

Munoz et al,[190] 2014 (1)	Retrospective study, data obtained from previous publications, doctorate theses and unpublished databases<br><br>Multicenter (n = 4) (Spain)	Underwent either hip fracture surgery, total knee replacement or total hip replacement<br><br>n = 2547	IV iron, either iron sucrose (100-200mg, 3 doses) or ferric carboxymaltose (600mg single dose)<br><br>+/- EPO	Oral iron or no iron	Lower infection rates in patients who received IV iron (+/- EPO) when compared with control (107/1000 (10.7%) vs. 97/361 (26.9%), p<0.01)<br><br>CDC definition used
Munoz et al,[187] 2014 (2)	Retrospective matched cohort study<br><br>Single center (Spain)	Lower limb arthroplasty<br><br>n = 394	IV iron, either iron sucrose (100-200mg, 3 doses) or ferric carboxymaltose (600mg single dose)	Usual medical care	No statistically significant differences in postoperative infection between those who received iron compared with usual care (3/182 (1.6%) vs. 6/182 (3.3%), p=0.502)<br><br>No definition provided
Peters et al,[188] 2008	Retrospective, matched cohort study<br><br>Multicenter (n = 5) (Germany)	Elective / emergency to perioperative ICU with anaemia<br><br>n = 95	Ferric carboxymaltose 500mg	Usual medical care	No statistically significant differences in postoperative infections between those who received iron compared with usual care (n=78) (6/26 (23%) vs. 14/52 (27%), p>0.05)<br><br>No definition provided
Zitt et al,[189] 2014	Prospective, observational cohort study<br><br>Single center (Austria)	CKD scheduled to start chronic dialysis treatment<br><br>n = 222	IV iron, ferric gluconate weekly	No iron	Iron therapy was associated with reduced cardiovascular and sepsis-related mortality (HR 1.76; 95% CI: 1.29 to 2.4)<br><br>No definition provided

Abbreviations: CDC, Centers for Disease Control and Prevention; CI, Confidence Interval; CKD, Chronic Kidney Disease; EPO, erythropoietin; HR, Hazard Ratio; ICD, International Classification of Diseases; ICU, Intensive Care Unit; IV, intravenous; OR, Odds Ratio.

© 2021 Shah AA et al. *JAMA Network Open.*

eTable 4. Summary of risk of bias assessment for NRS

Study ID	Confounding	Selection	Intervention classification	Deviation from intervention	Missing data	Measurement of outcome	Selection of reported result	Overall
Munoz 2014 (1)	Serious	Moderate	Low	Low	Low	Moderate	Low	Moderate
Munoz 2014 (2)	Serious	Moderate	Low	Low	Low	Moderate	Low	Moderate
Zitt 2014	Moderate	Low	Low	Low	Low	Moderate	Low	Moderate
Kurugano 2014	Moderate	Low	Moderate	Low	Low	Moderate	Low	Moderate
Ishida 2015	Moderate	Low	Low	Low	Low	Low	Low	Low
Kim 2018	Moderate	Low	Low	Low	Low	Moderate	Low	Moderate
Peters 2018	Moderate	Low	Low	Moderate	Low	Moderate	Low	Moderate
Ikuta 2019	Serious	Moderate	Low	Low	Low	Moderate	Low	Moderate

© 2021 Shah AA et al. *JAMA Network Open.*

eTable 5. Risk of bias assessments for individual RCTs

Study ID	Random sequence generation	Allocation concealment	Blinding of participants and personnel	Blinding of outcome assessment	Incomplete outcome data	Selective reporting	Other
Abdelazim 2017							
Abhilashini 2014							
Adhikary 2011							
Agarwal 2006							
Agarwal 2015							
Aggarwal 2003							
Akhtar 2018							
Al 2005							
Allen 2011							
Al-Momen 1996							
Anker 2009							
Athibovonsuk 2013							
Auerbach 2004							
Auerbach 2010							
Bager 2014							
Baillie 2010							
Barish (1) 2012							
Barish (2) 2012							
Bastit 2008							
Batool 2018							
Bayoumeu 2002							
Beck-da-Silva 2013							
Beguin 2013							
Bencaiova 2009							
Bernabeu-Wittel 2016							
Bhandal 20006							
Bhavi 2017							
Biboulet 2018							
Bielza 2020							
Birgegard 2010							
Birgegard 2016							
Bisbe 2014							

© 2021 Shah AA et al. *JAMA Network Open.*

Boomershine 2018							
Breymann 2008							
Breymann 2016							
Burden 2015							
Burns 1996							
Charles-Edwards 2019							
Charytan 2005							
Charytan 2012							
Cho 2016							
Cho 2018							
Coyne 2007							
Dalal 2018							
Damineni 2016							
Dangsuwan 2010							
Darwish 2019							
Deeba 2012							
Deng 2017							
Drexler 2020							
Dubey 2013							
Edwards 2009							
El Khouly 2017							
Evstatiev 2013							
Favrat 2014							
Ferrero-Barcelo 2019							
Fishbane 1995							
Friel 1995							
Froessler 2013							
Froessler 2016							
Garrido-Martin 2012							
Grote 2009							
Gupta 2014							
Hedenus 2007							
Hedenus 2014							
Henry 2007							
Holm 2017							
Iyoke 2017							
Jain 2013							

© 2021 Shah AA et al. *JAMA Network Open.*

Johansson 2015							
Kalra 2016							
Karkouti 2006							
Kasper 1998							
Keeler 2017							
Keller 2020							
Khalafallah 2010							
Khalafallah 2016							
Khalafallah 2018 (1)							
Khalafallah 2018 (2)							
Kim 2007							
Kim 2009							
Kim 2017							
Kochhar 2013							
Krayenbuehl 2011							
Kulnigg 2008							
Kulnigg-Dabsch 2013							
Kuo 2008							
Li 2008							
Lindgren 2009							
Litton 2016							
Maccio 2010							
MacDougall 1996							
MacDougall 2014							
Madi-Jebara 2004							
McMahon 2010							
Meyer 1996							
Michael 2002							
Montano-Pedroso 2018							
Moppett 2019							
Mudge 2012							
Na 2011							
Nanthini 2017							
Neeru 2012							
Neogi 2019							
Ng 2018							
Noronha 2018							

© 2021 Shah AA et al. *JAMA Network Open.*

Okonko 2008	Green	Green	Red	Green	Green	Green	Green
Olijhoek 2001	Green	Yellow	Green	Yellow	Green	Green	Green
Onken 2014	Green	Green	Green	Green	Green	Green	Green
Padmanabhan 2019	Green	Green	Red	Yellow	Green	Green	Green
Park 2019	Green	Green	Green	Yellow	Green	Green	Green
Pedrazzoli 2008	Yellow	Yellow	Green	Yellow	Red	Green	Green
Perello 2014	Green	Green	Green	Yellow	Green	Green	Green
Pieracci 2014	Green	Yellow	Green	Green	Red	Green	Green
Pollak 2001	Yellow	Green	Green	Yellow	Red	Yellow	Green
Ponikowski 2015	Green	Green	Green	Green	Green	Green	Green
Ponikowski 2020	Green	Green	Green	Green	Green	Green	Green
Price 2014	Green	Yellow	Red	Red	Green	Yellow	Green
Provenzano 2009	Green	Green	Green	Green	Green	Green	Green
Quinibi 2011	Green	Green	Green	Yellow	Green	Green	Yellow
Rathod 2015	Yellow	Yellow	Green	Yellow	Red	Red	Green
Razzaq 2017	Yellow	Yellow	Green	Yellow	Yellow	Yellow	Yellow
Reinisch 2013	Green	Green	Red	Yellow	Green	Green	Red
Richards 2020	Green	Green	Green	Green	Green	Green	Green
Roger 2017	Green	Green	Green	Yellow	Yellow	Red	Green
Rudra 2016	Yellow	Yellow	Green	Yellow	Green	Red	Green
Santer 2020	Green	Green	Green	Yellow	Green	Green	Green
Schijns 2020	Green	Yellow	Green	Yellow	Yellow	Green	Green
Schroder 2005	Green	Yellow	Red	Yellow	Red	Green	Green
Sedighi 2003	Yellow	Yellow	Yellow	Yellow	Green	Yellow	Green
Seid 2008	Green	Yellow	Green	Yellow	Green	Green	Green
Seid 2017	Green	Yellow	Red	Yellow	Green	Green	Red
Serrano-Trenas 2011	Green	Green	Green	Green	Green	Green	Green
Singh 1998	Yellow	Yellow	Green	Yellow	Yellow	Green	Yellow
Singh 2006	Yellow	Yellow	Green	Yellow	Green	Green	Green
Sloand 2004	Yellow	Yellow	Green	Yellow	Green	Green	Green
Spahn 2019	Green	Green	Green	Green	Green	Green	Green
Spinowitz 2008	Green	Green	Green	Yellow	Green	Green	Green
Steensma 2011	Green	Green	Red	Yellow	Green	Green	Yellow
Stoves 2001	Green	Green	Yellow	Yellow	Green	Green	Green
Toblli 2007	Green	Yellow	Green	Green	Green	Green	Green
Trenkwalder 2017	Yellow	Yellow	Green	Green	Green	Green	Green
Vadhan-Raj 2013	Green	Green	Green	Green	Green	Green	Green

© 2021 Shah AA et al. *JAMA Network Open.*

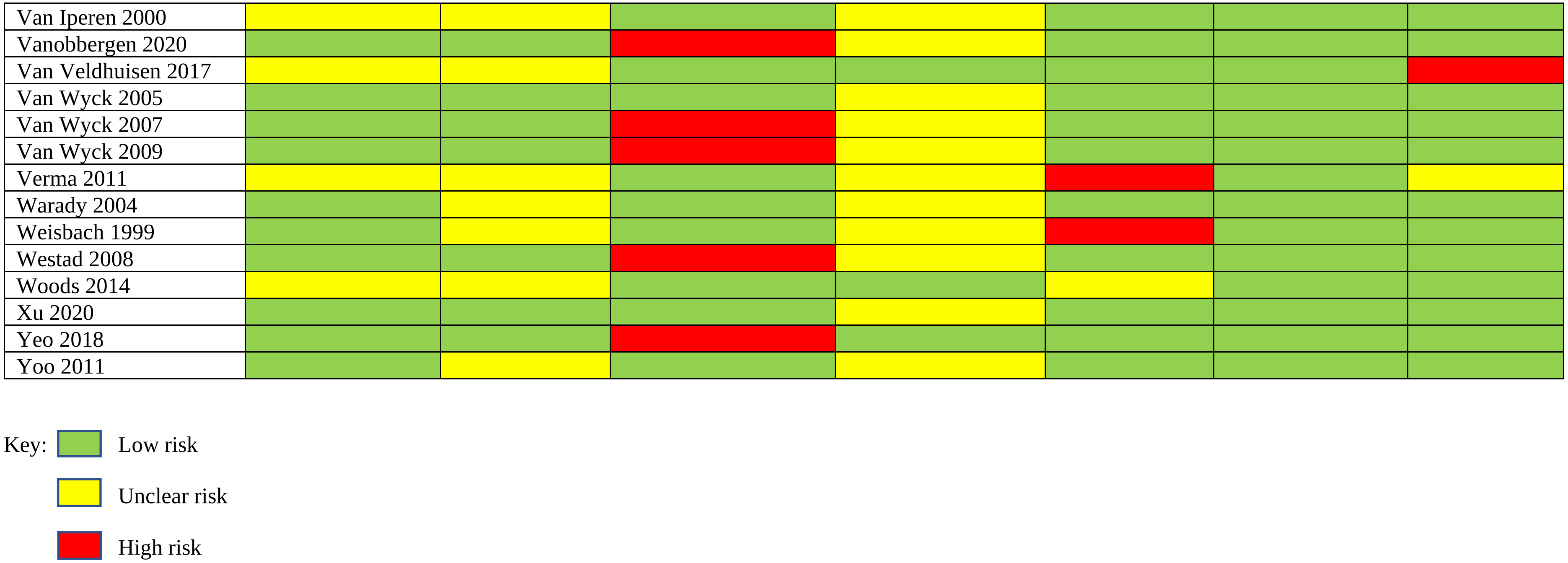

© 2021 Shah AA et al. *JAMA Network Open.*

eTable 6. Meta-regression analyses

Variable	No. of studies	Coefficient	95% Confidence Interval	p value
Ferritin	40	-.0000359	-0.0011785 to 0.0011067	0.950
Transferrin saturation	29	.0084419	-0.022902 to 0.0397858	0.585
Hemoglobin	36	-.0005157	-0.0054991 to 0.0044677	0.835
Study year	51	-.0083708	-0.0341953 to 0.0174536	0.518

Meta-regression analyses of ferritin, transferrin saturation, haemoglobin and study year on log risk ratio on infection.

© 2021 Shah AA et al. *JAMA Network Open.*

eTable 7. Summary of reporting of infection in included RCTs

Definition	Intravenous iron vs. oral iron, n	Intravenous iron vs. no iron, n	Total
Clinical discretion / not specified	23	26	49
Investigator defined	1	6	7
Classification system			
- Clavien Dindo	1		1
- MedDra	1	3	4
- CDC		2	2
- POMS		1	1
Characterisation			
- Anatomical sites (any)	9	18	24
Lung	6	15	20
Gastrointestinal	2	3	4
Genitourinary	6	7	35
Skin	4	1	4
Surgical wound	1	4	5
Other	4	3	6
Line related		1	1
- Pathogens	0		
- Antibiotic use	1		1

Abbreviations: CDC, Centers for Disease Control; MedDRA, Medical Dictionary for Regulatory Activities; POMS, Postoperative Morbidity Survey

Examples of investigator-defined definitions:

Administration of IV antibiotic along with H/o fever, abdominal pain and foul-smelling vaginal discharge

One of 'bronchitis, cellulitis, conjunctivitis, fungal infections, furuncles, C.difficile, line infections, pneumonia, nasopharyngitis, sepsis, skin infections, URI, UTI

'If clinical symptoms were evident, or positive blood CSF culture, or local lesions

Increase in body temperature to >38 degrees C with a requirment for IV antibiotics

Commencement, escalation or change of IV antibiotics for a confirmed or strongly suspected infection

Commencement, escalation or change of IV antibiotics for a confirmed or strongly suspected infection

Antibiotic upgrade

© 2021 Shah AA et al. *JAMA Network Open.*

eFigure 1. Risk of bias summary for RCTs

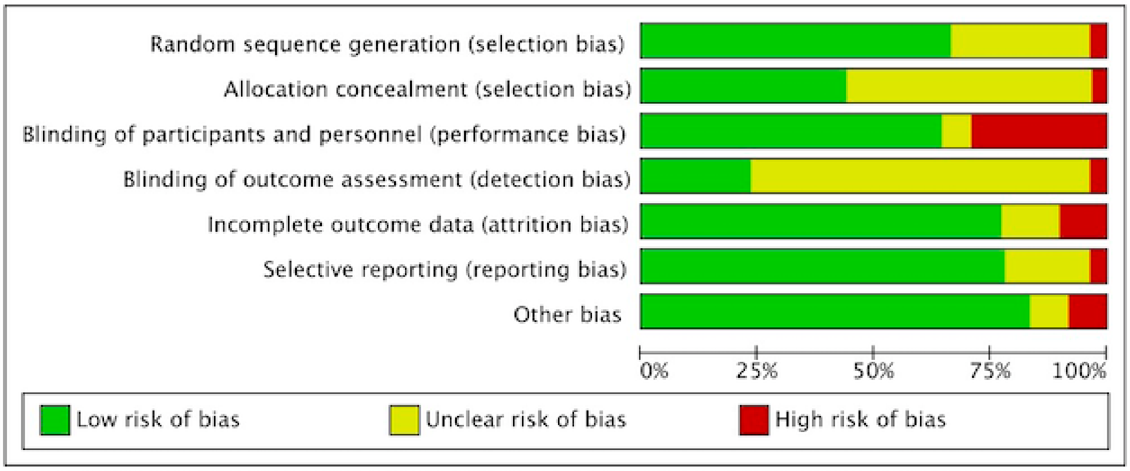

© 2021 Shah AA et al. *JAMA Network Open.*

eFigure 2. Funnel plot for primary outcome

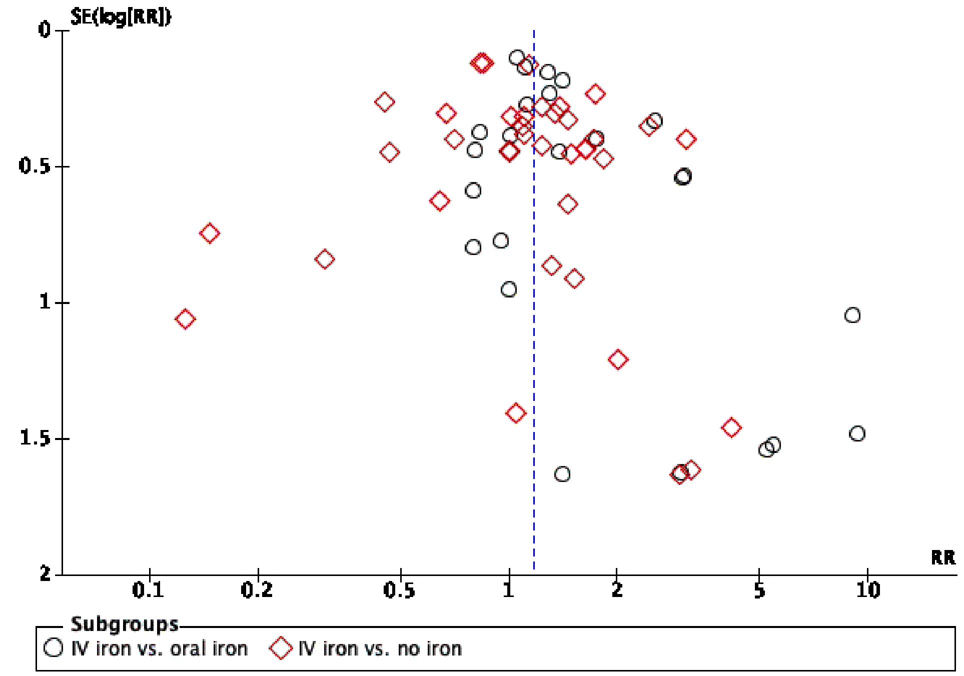

Funnel plot of standard error by log risk ratio for infection for the included studies in the meta-analysis.

Abbreviations: SE, Standard error; RR, Risk ratio

© 2021 Shah AA et al. *JAMA Network Open.*

eFigure 3. Sensitivity analysis for primary outcome

Study or Subgroup	Experimental Events	Experimental Total	Control Events	Control Total	Weight	Risk Ratio M-H, Random, 95% CI	A	B	C	D	E	F	G
**1.9.1 IV iron vs. oral iron**													
Keeler 2017	36	55	31	61	11.7%	1.29 [0.94, 1.76]	+	+	+	?	+	+	+
MacDougall 2014	114	304	111	312	15.6%	1.05 [0.86, 1.30]	+	+	+	?	+	+	+
Mudge 2012	10	51	12	51	3.7%	0.83 [0.40, 1.75]	+	+	+	?	+	+	+
Subtotal (95% CI)		410		424	31.0%	1.10 [0.93, 1.31]							
Total events	160		154										
Heterogeneity: Tau² = 0.00; Chi² = 1.73, df = 2 (P = 0.42); I² = 0%													
Test for overall effect: Z = 1.15 (P = 0.25)													
**1.9.2 IV iron vs. no iron**													
Anker 2009	50	305	24	254	7.7%	1.73 [1.10, 2.74]	+	+	+	+	+	+	+
Baillie 2010	27	559	22	559	6.0%	1.23 [0.71, 2.13]	+	?	+	+	+	+	+
Bernabeu-Wittel 2016	20	203	9	100	3.7%	1.09 [0.52, 2.32]	+	?	+	+	+	+	+
Coyne 2007	13	145	8	145	3.0%	1.63 [0.69, 3.80]	+	+	+	?	+	+	+
Kim 2017	1	228	0	226	0.2%	2.97 [0.12, 72.62]	+	+	+	+	+	+	+
Krayenbuehl 2011	10	43	6	47	2.6%	1.82 [0.72, 4.59]	+	+	+	+	+	+	+
Litton 2016	22	70	16	70	5.9%	1.38 [0.79, 2.39]	+	+	+	+	+	+	+
Michael 2002	3	1240	2	1250	0.8%	1.51 [0.25, 9.03]	+	?	+	+	+	+	+
Moppett 2019	8	39	12	41	3.5%	0.70 [0.32, 1.53]	+	+	+	+	+	+	+
Ponikowski 2020	103	559	121	551	14.5%	0.84 [0.66, 1.06]	+	+	+	+	+	+	+
Richards 2020	7	234	15	234	2.8%	0.47 [0.19, 1.12]	+	+	+	+	+	+	+
Serrano-Trenas 2011	16	100	13	199	4.2%	2.45 [1.23, 4.89]	+	+	+	+	+	+	+
Spahn 2019	88	243	77	241	14.0%	1.13 [0.88, 1.45]	+	+	+	+	+	+	+
Subtotal (95% CI)		3968		3917	69.0%	1.19 [0.95, 1.49]							
Total events	368		325										
Heterogeneity: Tau² = 0.06; Chi² = 22.30, df = 12 (P = 0.03); I² = 46%													
Test for overall effect: Z = 1.54 (P = 0.12)													
**Total (95% CI)**		4378		4341	100.0%	1.15 [0.98, 1.35]							
Total events	528		479										
Heterogeneity: Tau² = 0.03; Chi² = 23.97, df = 15 (P = 0.07); I² = 37%													
Test for overall effect: Z = 1.73 (P = 0.08)													
Test for subgroup differences: Chi² = 0.29, df = 1 (P = 0.59), I² = 0%													

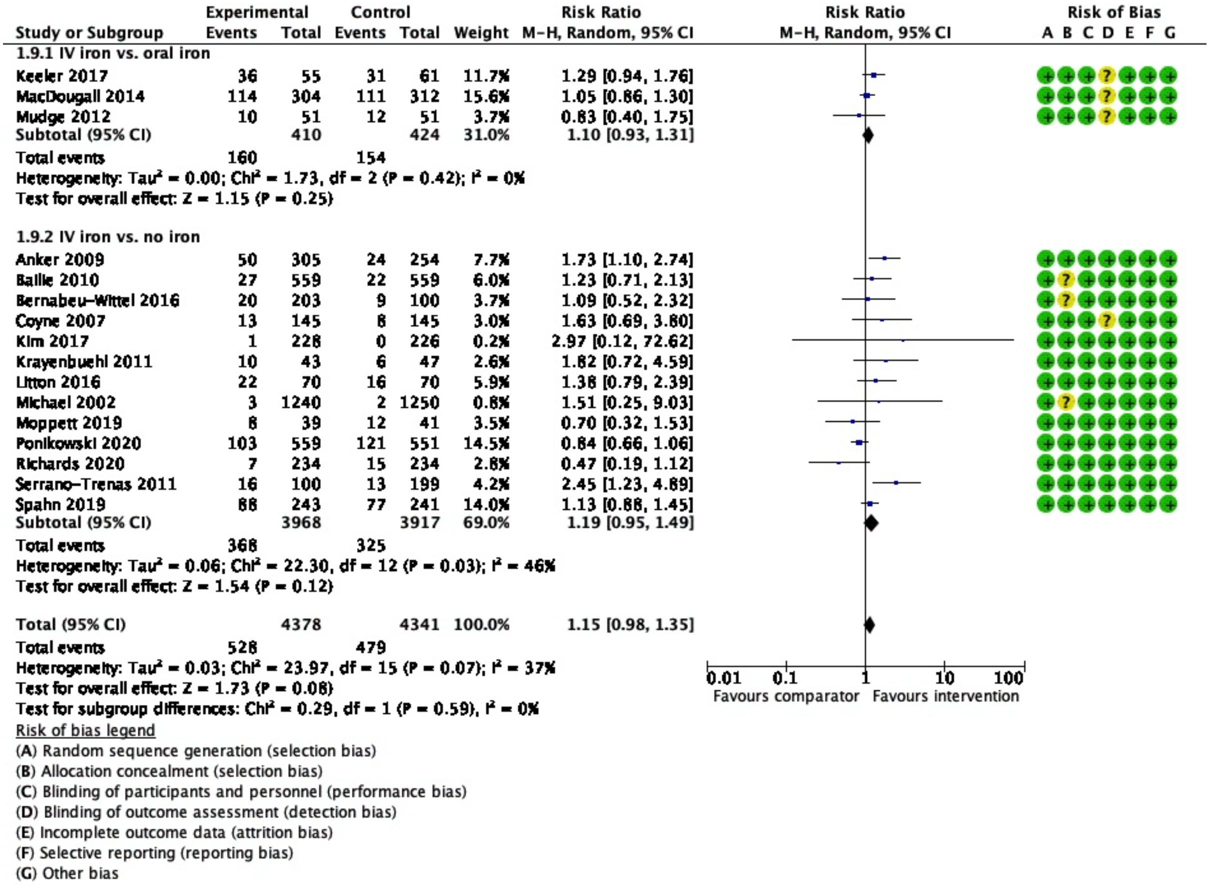

Risk of bias legend
(A) Random sequence generation (selection bias)
(B) Allocation concealment (selection bias)
(C) Blinding of participants and personnel (performance bias)
(D) Blinding of outcome assessment (detection bias)
(E) Incomplete outcome data (attrition bias)
(F) Selective reporting (reporting bias)
(G) Other bias

Association between risk of infection and intravenous iron by removing studies at high risk of bias. The risk ratios were calculated using a random-effects model with Mantel-Haenszel weighting. The size of the data markers indicates the weight of the study. Error bars indicate 95% CIs. Risk of bias assessments for each study are displayed on the right

© 2021 Shah AA et al. *JAMA Network Open.*

eFigure 4. Subgroup analysis by clinical setting

Study or Subgroup	Experimental Events	Experimental Total	Control Events	Control Total	Weight	Risk Ratio M-H, Random, 95% CI
**1.10.1 Heart failure**						
Anker et al,[41] 2009	50	305	24	254	3.1%	1.73 [1.10, 2.74]
Okonko et al,[134] 2008	2	24	3	11	0.4%	0.31 [0.06, 1.58]
Ponikowski et al,[illegible] 2020	102	559	121	551	4.9%	0.83 [0.66, 1.05]
**Subtotal (95% CI)**		**888**		**816**	**8.5%**	**0.98 [0.50, 1.95]**
Total events	154		148			
Heterogeneity: Tau^2 = 0.25; Chi^2 = 9.78, df = 2 (P = 0.008); I^2 = 80%						
Test for overall effect: Z = 0.05 (P = 0.96)						
**1.10.2 Chronic Kidney Disease**						
Agarwal et al,[34] 2015	37	67	27	69	3.8%	1.41 [0.98, 2.03]
Coyne et al,[72] 2007	13	145	8	145	1.4%	1.63 [0.69, 3.80]
Fishbane et al,[86] 1995	2	25	5	50	0.5%	0.80 [0.17, 3.84]
Kalra et al,[99] 2016	56	112	53	117	4.6%	1.10 [0.84, 1.45]
Kuo et al,[115] 2008	2	55	1	55	0.2%	2.00 [0.19, 21.42]
MacDougall et al,[119] 2014	114	304	111	312	5.2%	1.05 [0.86, 1.30]
Michael et al,[124] 2002	3	1240	2	1250	0.4%	1.51 [0.25, 9.03]
Qunibi et al,[147] 2011	20	147	8	103	1.6%	1.75 [0.80, 3.82]
Singh et al,[159] 2006	9	75	9	75	1.3%	1.00 [0.42, 2.38]
Van Wyck et al,[173] 2005	2	79	0	82	0.1%	5.19 [0.25, 106.38]
**Subtotal (95% CI)**		**2249**		**2258**	**19.1%**	**1.15 [1.00, 1.33]**
Total events	258		224			
Heterogeneity: Tau^2 = 0.00; Chi^2 = 5.32, df = 9 (P = 0.81); I^2 = 0%						
Test for overall effect: Z = 1.97 (P = 0.05)						
**1.10.3 Critical care**						
Litton et al,[107] 2016	22	70	16	70	2.5%	1.38 [0.79, 2.39]
Pieracci et al,[141] 2014	44	75	52	75	4.9%	0.85 [0.66, 1.08]
Xu et al,[179] 2020	14	75	21	75	2.3%	0.67 [0.37, 1.21]
**Subtotal (95% CI)**		**220**		**220**	**9.7%**	**0.90 [0.65, 1.26]**
Total events	80		89			
Heterogeneity: Tau^2 = 0.04; Chi^2 = 3.48, df = 2 (P = 0.18); I^2 = 43%						
Test for overall effect: Z = 0.59 (P = 0.56)						
**1.10.4 Inflammatory bowel disease**						
Evstatiev et al,[82] 2013	20	105	13	99	2.1%	1.45 [0.76, 2.76]
Kulnigg et al,[113] 2008	18	137	6	63	1.3%	1.38 [0.58, 3.31]
Kulnigg-Dabsch et al,[114] 2013	3	16	0	9	0.2%	4.12 [0.24, 71.78]
Lindgren et al,[117] 2009	12	45	4	46	1.0%	3.07 [1.07, 8.80]
Reinisch et al,[149] 2013	1	233	0	109	0.1%	1.41 [0.06, 34.34]
Schroder et al,[154] 2005	2	22	0	24	0.1%	5.43 [0.28, 107.33]
**Subtotal (95% CI)**		**558**		**350**	**4.8%**	**1.73 [1.11, 2.71]**
Total events	56		23			
Heterogeneity: Tau^2 = 0.00; Chi^2 = 2.64, df = 5 (P = 0.76); I^2 = 0%						
Test for overall effect: Z = 2.40 (P = 0.02)						
**1.10.5 Mixed/Medical**						
Baillie et al,[46] 2010	27	559	22	559	2.5%	1.23 [0.71, 2.13]
Barish (1) et al,[47] 2012	23	343	18	360	2.3%	1.34 [0.74, 2.44]
Barish (2) et al,[47] 2012	1	366	8	369	0.3%	0.13 [0.02, 1.00]
Burns et al,[65] 1996	4	11	3	12	0.7%	1.45 [0.42, 5.10]
Favrat et al,[84] 2014	13	145	8	145	1.4%	1.63 [0.69, 3.80]
Krayenbuehl et al,[112] 2011	10	43	6	47	1.2%	1.82 [0.72, 4.59]
Santer et al,[152] 2020	7	24	7	24	1.3%	1.00 [0.41, 2.42]
Vadhan-Raj et al,[168] 2013	33	608	10	200	1.9%	1.09 [0.54, 2.16]
**Subtotal (95% CI)**		**2099**		**1716**	**11.6%**	**1.24 [0.94, 1.64]**
Total events	118		82			
Heterogeneity: Tau^2 = 0.00; Chi^2 = 6.32, df = 7 (P = 0.50); I^2 = 0%						
Test for overall effect: Z = 1.53 (P = 0.13)						
**1.10.6 Obstetrics**						
Bencaiova et al,[53] 2009	9	130	1	130	0.3%	9.00 [1.16, 70.03]
Breymann et al,[63] 2008	19	277	4	177	1.0%	3.04 [1.05, 8.77]
Khalafallah et al,[104] 2010	0	92	0	91		Not estimable
Neogi et al,[131] 2019	13	934	13	947	1.6%	1.01 [0.47, 2.18]
Singh et al,[160] 1998	0	30	0	30		Not estimable
Van Wyck et al,[172] 2007	24	174	22	178	2.6%	1.12 [0.65, 1.91]
Vanobberghen et al,[174] 2020	32	114	25	116	3.1%	1.30 [0.83, 2.05]
Verma et al,[182] 2011	1	75	0	75	0.1%	3.00 [0.12, 72.49]
**Subtotal (95% CI)**		**1826**		**1744**	**8.7%**	**1.42 [0.95, 2.13]**
Total events	98		65			
Heterogeneity: Tau^2 = 0.07; Chi^2 = 7.03, df = 5 (P = 0.22); I^2 = 29%						
Test for overall effect: Z = 1.72 (P = 0.09)						
**1.10.7 Oncology**						
Bastit et al,[48] 2008	18	200	39	196	2.7%	0.45 [0.27, 0.76]
Beguin et al,[52] 2013	10	50	7	52	1.3%	1.49 [0.61, 3.60]
Hedenus et al,[93] 2007	18	33	6	34	1.6%	3.09 [1.40, 6.81]
Henry et al,[94] 2007	6	41	16	88	1.4%	0.80 [0.34, 1.91]
Pedrazzoli et al,[139] 2008	1	73	1	76	0.2%	1.04 [0.07, 16.34]
Steensma et al,[164] 2011	28	164	11	163	2.0%	2.53 [1.30, 4.91]
**Subtotal (95% CI)**		**561**		**609**	**9.1%**	**1.30 [0.61, 2.76]**
Total events	81		80			
Heterogeneity: Tau^2 = 0.65; Chi^2 = 24.67, df = 5 (P = 0.0002); I^2 = 80%						
Test for overall effect: Z = 0.67 (P = 0.50)						
**1.10.8 Paediatrics**						
Friel et al,[87] 1995	9	14	7	12	2.2%	1.10 [0.59, 2.04]
Meyer et al,[123] 1996	2	21	2	21	0.4%	1.00 [0.16, 6.45]
Pollak et al,[142] 2001	2	10	4	19	0.5%	0.95 [0.21, 4.32]
**Subtotal (95% CI)**		**45**		**52**	**3.1%**	**1.07 [0.62, 1.85]**
Total events	13		13			
Heterogeneity: Tau^2 = 0.00; Chi^2 = 0.04, df = 2 (P = 0.98); I^2 = 0%						
Test for overall effect: Z = 0.25 (P = 0.80)						
**1.10.9 Restless leg syndrome**						
Allen et al,[40] 2011	3	24	2	21	0.4%	1.31 [0.24, 7.12]
Grote et al,[91] 2009	1	29	0	31	0.1%	3.20 [0.14, 75.55]
Trenkwalder et al,[166] 2017	11	58	8	52	1.4%	1.23 [0.54, 2.83]
**Subtotal (95% CI)**		**111**		**104**	**2.0%**	**1.31 [0.63, 2.71]**
Total events	15		10			
Heterogeneity: Tau^2 = 0.00; Chi^2 = 0.33, df = 2 (P = 0.85); I^2 = 0%						
Test for overall effect: Z = 0.73 (P = 0.46)						
**1.10.10 Surgery/perioperative**						
Bernabeu-Wittel et al,[54] 2016	20	203	9	100	1.7%	1.09 [0.52, 2.32]
Bielza et al,[58] 2020	17	126	17	127	2.2%	1.01 [0.54, 1.88]
Bisbe et al,[61] 2014	4	60	0	62	0.1%	9.30 [0.51, 169.01]
Froessler et al,[illegible] 2016	4	40	5	32	0.8%	0.64 [0.19, 2.19]
Johansson et al,[98] 2015	12	30	7	30	1.6%	1.71 [0.78, 3.75]
Keeler et al,[102] 2017	36	55	31	61	4.2%	1.29 [0.94, 1.76]
Khalafallah et al,[106] 2016	2	103	13	98	0.6%	0.15 [0.03, 0.63]
Kim et al,[110] 2017	1	228	0	226	0.1%	2.97 [0.12, 72.62]
Moppett et al,[126] 2019	8	39	12	41	1.6%	0.70 [0.32, 1.53]
Mudge et al,[127] 2012	10	51	12	51	1.7%	0.83 [0.40, 1.75]
Padmanabhan et al,[137] 2019	4	20	5	20	0.8%	0.80 [0.25, 2.55]
Richards et al,[150] 2020	7	234	15	234	1.3%	0.47 [0.19, 1.12]
Serrano-Trenas et al,[158] 2011	16	100	13	199	1.9%	2.45 [1.23, 4.89]
Spahn et al,[162] 2019	88	243	77	241	4.8%	1.13 [0.88, 1.45]
**Subtotal (95% CI)**		**1532**		**1522**	**23.4%**	**1.04 [0.80, 1.36]**
Total events	229		216			
Heterogeneity: Tau^2 = 0.09; Chi^2 = 24.05, df = 13 (P = 0.03); I^2 = 46%						
Test for overall effect: Z = 0.31 (P = 0.76)						
**Total (95% CI)**		**10089**		**9391**	**100.0%**	**1.17 [1.04, 1.31]**
Total events	1102		950			
Heterogeneity: Tau^2 = 0.05; Chi^2 = 96.38, df = 61 (P = 0.003); I^2 = 37%						
Test for overall effect: Z = 2.66 (P = 0.008)						
Test for subgroup differences: Chi^2 = 7.53, df = 9 (P = 0.58), I^2 = 0%						

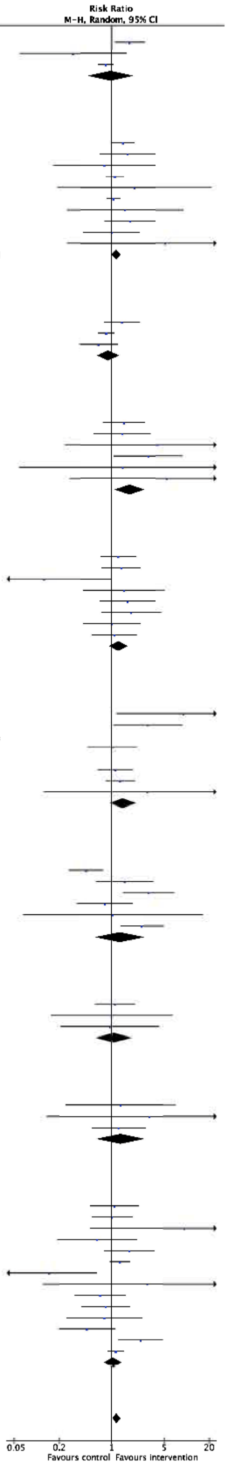

eFigure 5. Subgroup analysis by (a) dosing schedule (b) type of preparation and (c) iron profile at enrolment on the risk of infection.

(a) Dosing schedule

Study or Subgroup	Experimental Events	Experimental Total	Control Events	Control Total	Weight	Risk Ratio M-H, Random, 95% CI
**1.12.1 Single dosing**						
Baille 2010	27	559	22	559	2.5%	1.23 [0.71, 2.13]
Barish (1) 2012	23	343	18	360	2.3%	1.34 [0.74, 2.44]
Bernabeu-Wittel 2016	20	203	9	100	1.7%	1.09 [0.52, 2.32]
Bisbe 2014	4	60	0	62	0.1%	9.30 [0.51, 169.01]
Breymann 2008	19	277	4	177	1.0%	3.04 [1.05, 8.77]
Favrat 2014	13	145	8	145	1.4%	1.63 [0.69, 3.80]
Froessler 2016	4	40	5	32	0.8%	0.64 [0.19, 2.19]
Johansson 2015	12	30	7	30	1.6%	1.71 [0.78, 3.75]
Kalra 2016	56	112	53	117	4.6%	1.10 [0.84, 1.45]
Keeler 2017	36	55	31	61	4.2%	1.29 [0.94, 1.76]
Khalafallah 2010	0	92	0	91		Not estimable
Khalafallah 2016	2	103	13	98	0.6%	0.15 [0.03, 0.63]
Kim 2017	1	228	0	226	0.1%	2.97 [0.12, 72.62]
Kulnigg 2008	18	137	6	63	1.3%	1.38 [0.58, 3.31]
Kulnigg-Dabsch 2013	3	16	0	9	0.2%	4.12 [0.24, 71.78]
Michael 2002	3	1240	2	1250	0.4%	1.51 [0.25, 9.03]
Mudge 2012	10	51	12	51	1.7%	0.83 [0.40, 1.75]
Padmanabhan 2019	4	20	5	20	0.8%	0.80 [0.25, 2.55]
Reinisch 2013	1	233	0	109	0.1%	1.41 [0.06, 34.34]
Richards 2020	7	234	15	234	1.3%	0.47 [0.19, 1.12]
Santer 2020	7	24	7	24	1.3%	1.00 [0.41, 2.42]
Singh 1998	0	30	0	30		Not estimable
Spahn 2019	88	243	77	241	4.8%	1.13 [0.88, 1.45]
Trenkwalder 2017	11	58	8	52	1.4%	1.23 [0.54, 2.83]
Van Wyck 2007	24	174	22	178	2.6%	1.12 [0.65, 1.91]
Subtotal (95% CI)		4707		4319	36.9%	1.15 [1.01, 1.32]
Total events	393		324			
Heterogeneity: $Tau^2 = 0.00$; $Chi^2 = 22.94$, df = 22 (P = 0.41); $I^2 = 4\%$						
Test for overall effect: Z = 2.11 (P = 0.04)						
**1.12.2 Multiple dosing**						
Agarwal 2015	37	67	27	69	3.8%	1.41 [0.98, 2.03]
Allen 2011	3	24	2	21	0.4%	1.31 [0.24, 7.12]
Anker 2009	50	305	24	254	3.1%	1.73 [1.10, 2.74]
Barish (2) 2012	1	366	8	369	0.3%	0.13 [0.02, 1.00]
Bastit 2008	18	200	39	196	2.7%	0.45 [0.27, 0.76]
Beguin 2013	10	50	7	52	1.3%	1.49 [0.61, 3.60]
Bencaiova 2009	9	130	1	130	0.3%	9.00 [1.16, 70.03]
Bielza 2020	17	126	17	127	2.2%	1.01 [0.54, 1.88]
Burns 1996	4	11	3	12	0.7%	1.45 [0.42, 5.10]
Coyne 2007	13	145	8	145	1.4%	1.63 [0.69, 3.80]
Evstatiev 2013	20	105	13	99	2.1%	1.45 [0.76, 2.76]
Fishbane 1995	2	25	5	50	0.5%	0.80 [0.17, 3.84]
Friel 1995	9	14	7	12	2.2%	1.10 [0.59, 2.04]
Grote 2009	1	29	0	31	0.1%	3.20 [0.14, 75.55]
Hedenus 2007	18	33	6	34	1.6%	3.09 [1.40, 6.81]
Henry 2007	6	41	16	88	1.4%	0.80 [0.34, 1.91]
Krayenbuehl 2011	10	43	6	47	1.2%	1.82 [0.72, 4.59]
Kuo 2008	2	55	1	55	0.2%	2.00 [0.19, 21.42]
Lindgren 2009	12	45	4	46	1.0%	3.07 [1.07, 8.80]
Litton 2016	22	70	16	70	2.5%	1.38 [0.79, 2.39]
MacDougall 2014	114	304	111	312	5.2%	1.05 [0.86, 1.30]
Meyer 1996	2	21	2	21	0.4%	1.00 [0.16, 6.45]
Moppett 2019	8	39	12	41	1.6%	0.70 [0.32, 1.53]
Neogi 2019	13	934	13	947	1.6%	1.01 [0.47, 2.18]
Okonko 2008	2	24	3	11	0.4%	0.31 [0.06, 1.58]
Pedrazzoli 2008	1	73	1	76	0.2%	1.04 [0.07, 16.34]
Pieracci 2014	44	75	52	75	4.9%	0.85 [0.66, 1.08]
Pollak 2001	2	10	4	19	0.5%	0.95 [0.21, 4.32]
Ponikowski 2020	102	559	121	551	4.9%	0.83 [0.66, 1.05]
Qunibi 2011	20	147	8	103	1.6%	1.75 [0.80, 3.82]
Schroder 2005	2	22	0	24	0.1%	5.43 [0.28, 107.33]
Serrano-Trenas 2011	16	100	13	199	1.9%	2.45 [1.23, 4.89]
Singh 2006	9	75	9	75	1.3%	1.00 [0.42, 2.38]
Steensma 2011	28	164	11	163	2.0%	2.53 [1.30, 4.91]
Vadhan-Raj 2013	33	608	10	200	1.9%	1.09 [0.54, 2.16]
Van Wyck 2005	2	79	0	82	0.1%	5.19 [0.25, 106.38]
Vanobberghen 2020	32	114	25	116	3.1%	1.30 [0.83, 2.05]
Verma 2011	1	75	0	75	0.1%	3.00 [0.12, 72.49]
Xu 2020	14	75	21	75	2.3%	0.67 [0.37, 1.21]
Subtotal (95% CI)		5382		5072	63.1%	1.20 [1.02, 1.40]
Total events	709		626			
Heterogeneity: $Tau^2 = 0.09$; $Chi^2 = 73.02$, df = 38 (P = 0.0005); $I^2 = 48\%$						
Test for overall effect: Z = 2.19 (P = 0.03)						
**Total (95% CI)**		10089		9391	100.0%	1.17 [1.04, 1.31]
Total events	1102		950			
Heterogeneity: $Tau^2 = 0.05$; $Chi^2 = 96.38$, df = 61 (P = 0.003); $I^2 = 37\%$						
Test for overall effect: Z = 2.66 (P = 0.008)						
Test for subgroup differences: $Chi^2 = 0.11$, df = 1 (P = 0.74), $I^2 = 0\%$						

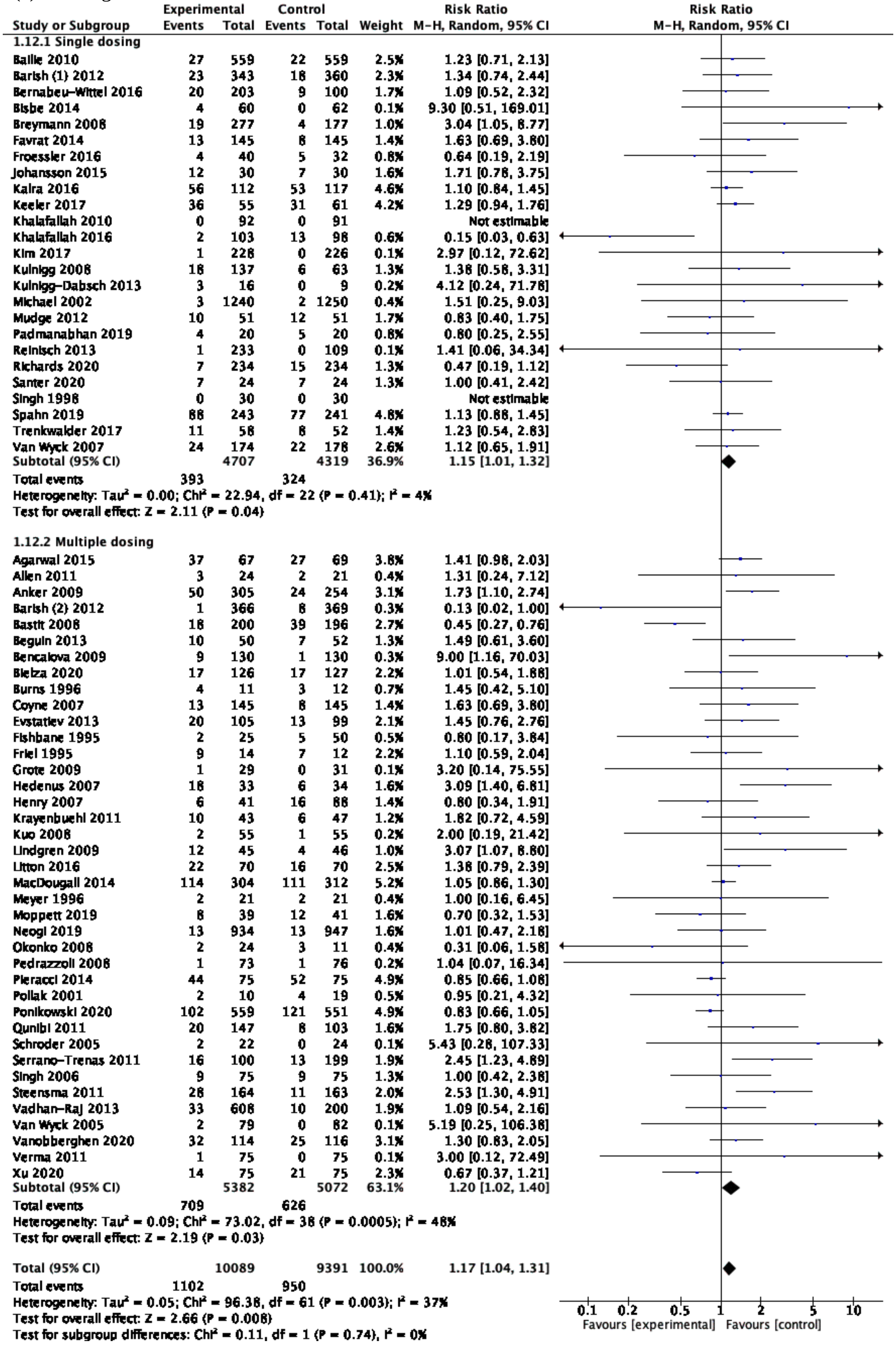

© 2021 Shah AA et al. *JAMA Network Open.*

(b) Type of preparation

Study or Subgroup	Experimental Events	Experimental Total	Control Events	Control Total	Weight	Risk Ratio M-H, Random, 95% CI
**1.11.1 Ferric carboxymaltose**						
Allen 2011	3	24	2	21	0.4%	1.31 [0.24, 7.12]
Anker 2009	50	305	24	254	3.1%	1.73 [1.10, 2.74]
Baille 2010	27	559	22	559	2.5%	1.23 [0.71, 2.13]
Barish (1) 2012	23	343	18	360	2.3%	1.34 [0.74, 2.44]
Barish (2) 2012	1	366	8	369	0.3%	0.13 [0.02, 1.00]
Bernabeu-Wittel 2016	20	203	9	100	1.7%	1.09 [0.52, 2.32]
Bisbe 2014	4	60	0	62	0.1%	9.30 [0.51, 169.01]
Breymann 2008	19	277	4	177	1.0%	3.04 [1.05, 8.77]
Evstatiev 2013	20	105	13	99	2.1%	1.45 [0.76, 2.76]
Favrat 2014	13	145	8	145	1.4%	1.63 [0.69, 3.80]
Froessler 2016	4	40	5	32	0.8%	0.64 [0.19, 2.19]
Keeler 2017	36	55	31	61	4.2%	1.29 [0.94, 1.76]
Khalafallah 2016	2	103	13	98	0.6%	0.15 [0.03, 0.63]
Kim 2017	1	228	0	226	0.1%	2.97 [0.12, 72.62]
Kulnigg 2008	18	137	6	63	1.3%	1.38 [0.58, 3.31]
Kulnigg-Dabsch 2013	3	16	0	9	0.2%	4.12 [0.24, 71.78]
Litton 2016	22	70	16	70	2.5%	1.38 [0.79, 2.39]
MacDougall 2014	114	304	111	312	5.2%	1.05 [0.86, 1.30]
Padmanabhan 2019	4	20	5	20	0.8%	0.80 [0.25, 2.55]
Ponikowski 2020	102	559	121	551	4.9%	0.83 [0.66, 1.05]
Qunibi 2011	20	147	8	103	1.6%	1.75 [0.80, 3.82]
Richards 2020	7	234	15	234	1.3%	0.47 [0.19, 1.12]
Santer 2020	7	24	7	24	1.3%	1.00 [0.41, 2.42]
Spahn 2019	88	243	77	241	4.8%	1.13 [0.88, 1.45]
Trenkwalder 2017	11	58	8	52	1.4%	1.23 [0.54, 2.83]
Van Wyck 2007	24	174	22	178	2.6%	1.12 [0.65, 1.91]
Vanobberghen 2020	32	114	25	116	3.1%	1.30 [0.83, 2.05]
Xu 2020	14	75	21	75	2.3%	0.67 [0.37, 1.21]
Subtotal (95% CI)		4988		4611	54.0%	1.13 [0.98, 1.31]
Total events	689		599			
Heterogeneity: Tau² = 0.04; Chi² = 41.19, df = 27 (P = 0.04); I² = 34%						
Test for overall effect: Z = 1.71 (P = 0.09)						
**1.11.2 Iron sucrose**						
Agarwal 2015	37	67	27	69	3.8%	1.41 [0.98, 2.03]
Bastit 2008	18	200	39	196	2.7%	0.45 [0.27, 0.76]
Beguin 2013	10	50	7	52	1.3%	1.49 [0.61, 3.60]
Bencalova 2009	9	130	1	130	0.3%	9.00 [1.16, 70.03]
Bielza 2020	17	126	17	127	2.2%	1.01 [0.54, 1.88]
Grote 2009	1	29	0	31	0.1%	3.20 [0.14, 75.55]
Hedenus 2007	18	33	6	34	1.6%	3.09 [1.40, 6.81]
Krayenbuehl 2011	10	43	6	47	1.2%	1.82 [0.72, 4.59]
Kuo 2008	2	55	1	55	0.2%	2.00 [0.19, 21.42]
Lindgren 2009	12	45	4	46	1.0%	3.07 [1.07, 8.80]
Meyer 1996	2	21	2	21	0.4%	1.00 [0.16, 6.45]
Moppett 2019	8	39	12	41	1.6%	0.70 [0.32, 1.53]
Neogi 2019	13	934	13	947	1.6%	1.01 [0.47, 2.18]
Okonko 2008	2	24	3	11	0.4%	0.31 [0.06, 1.58]
Pieracci 2014	44	75	52	75	4.9%	0.85 [0.66, 1.08]
Schroder 2005	2	22	0	24	0.1%	5.43 [0.28, 107.33]
Serrano-Trenas 2011	16	100	13	199	1.9%	2.45 [1.23, 4.89]
Singh 2006	9	75	9	75	1.3%	1.00 [0.42, 2.38]
Van Wyck 2005	2	79	0	82	0.1%	5.19 [0.25, 106.38]
Verma 2011	1	75	0	75	0.1%	3.00 [0.12, 72.49]
Subtotal (95% CI)		2222		2337	26.9%	1.28 [0.95, 1.74]
Total events	233		212			
Heterogeneity: Tau² = 0.20; Chi² = 46.12, df = 19 (P = 0.0005); I² = 59%						
Test for overall effect: Z = 1.60 (P = 0.11)						
**1.11.3 Iron isomaltoside**						
Johansson 2015	12	30	7	30	1.6%	1.71 [0.78, 3.75]
Kalra 2016	56	112	53	117	4.6%	1.10 [0.84, 1.45]
Reinisch 2013	1	233	0	109	0.1%	1.41 [0.06, 34.34]
Subtotal (95% CI)		375		256	6.3%	1.16 [0.90, 1.50]
Total events	69		60			
Heterogeneity: Tau² = 0.00; Chi² = 1.12, df = 2 (P = 0.57); I² = 0%						
Test for overall effect: Z = 1.13 (P = 0.26)						
**1.11.4 Ferric gluconate**						
Coyne 2007	13	145	8	145	1.4%	1.63 [0.69, 3.80]
Henry 2007	6	41	16	88	1.4%	0.80 [0.34, 1.91]
Michael 2002	3	1240	2	1250	0.4%	1.51 [0.25, 9.03]
Pedrazzoli 2008	1	73	1	76	0.2%	1.04 [0.07, 16.34]
Steensma 2011	28	164	11	163	2.0%	2.53 [1.30, 4.91]
Subtotal (95% CI)		1663		1722	5.3%	1.59 [1.00, 2.53]
Total events	51		38			
Heterogeneity: Tau² = 0.03; Chi² = 4.38, df = 4 (P = 0.36); I² = 9%						
Test for overall effect: Z = 1.98 (P = 0.05)						
**1.11.5 Ferumoxytol**						
Vadhan-Raj 2013	33	608	10	200	1.9%	1.09 [0.54, 2.16]
Subtotal (95% CI)		608		200	1.9%	1.09 [0.54, 2.16]
Total events	33		10			
Heterogeneity: Not applicable						
Test for overall effect: Z = 0.23 (P = 0.82)						
**1.11.6 Iron dextran**						
Burns 1996	4	11	3	12	0.7%	1.45 [0.42, 5.10]
Fishbane 1995	2	25	5	50	0.5%	0.80 [0.17, 3.84]
Friel 1995	9	14	7	12	2.2%	1.10 [0.59, 2.04]
Singh 1998	0	30	0	30		Not estimable
Subtotal (95% CI)		80		104	3.4%	1.12 [0.66, 1.88]
Total events	15		15			
Heterogeneity: Tau² = 0.00; Chi² = 0.35, df = 2 (P = 0.84); I² = 0%						
Test for overall effect: Z = 0.41 (P = 0.68)						
**1.11.7 Iron polymaltose**						
Khalafallah 2010	0	92	0	91		Not estimable
Mudge 2012	10	51	12	51	1.7%	0.83 [0.40, 1.75]
Pollak 2001	2	10	4	19	0.5%	0.95 [0.21, 4.32]
Subtotal (95% CI)		153		161	2.2%	0.85 [0.44, 1.67]
Total events	12		16			
Heterogeneity: Tau² = 0.00; Chi² = 0.02, df = 1 (P = 0.88); I² = 0%						
Test for overall effect: Z = 0.46 (P = 0.65)						
Total (95% CI)		10089		9391	100.0%	1.17 [1.04, 1.31]
Total events	1102		950			
Heterogeneity: Tau² = 0.05; Chi² = 96.38, df = 61 (P = 0.003); I² = 37%						
Test for overall effect: Z = 2.66 (P = 0.008)						
Test for subgroup differences: Chi² = 3.18, df = 6 (P = 0.79), I² = 0%						

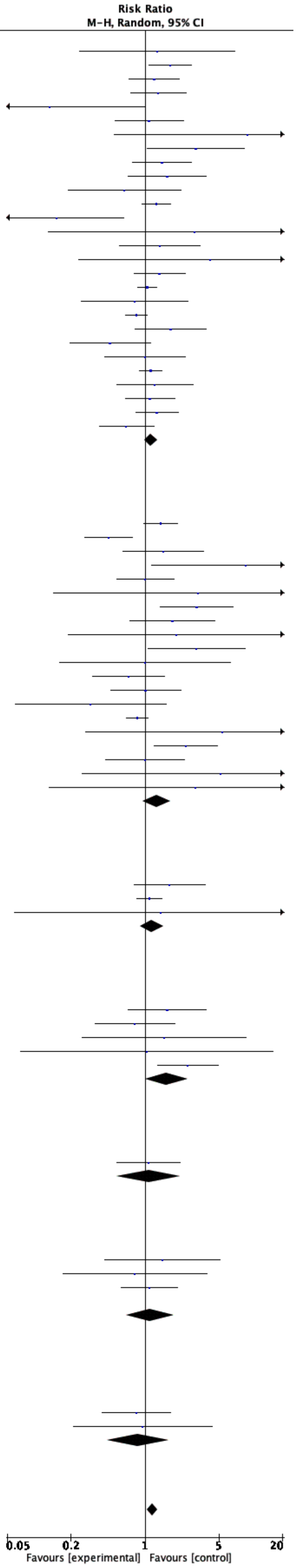

© 2021 Shah AA et al. *JAMA Network Open.*

(c) Iron profile at enrolment

Study or Subgroup	Experimental Events	Experimental Total	Control Events	Control Total	Weight	Risk Ratio M–H, Random, 95% CI
**1.13.1 Iron deficiency anaemia**						
Barish (1) 2012	23	343	18	360	2.4%	1.34 [0.74, 2.44]
Barish (2) 2012	1	366	8	369	0.3%	0.13 [0.02, 1.00]
Favrat 2014	13	145	8	145	1.5%	1.63 [0.69, 3.80]
Froessler 2016	4	40	5	32	0.8%	0.64 [0.19, 2.19]
Khalafallah 2010	0	92	0	91		Not estimable
Kulnigg–Dabsch 2013	3	16	0	9	0.2%	4.12 [0.24, 71.78]
Reinisch 2013	1	233	0	109	0.1%	1.41 [0.06, 34.34]
Schroder 2005	2	22	0	24	0.2%	5.43 [0.28, 107.33]
Singh 1998	0	30	0	30		Not estimable
Spahn 2019	88	243	77	241	4.8%	1.13 [0.88, 1.45]
Steensma 2011	28	164	11	163	2.1%	2.53 [1.30, 4.91]
Vanobberghen 2020	32	114	25	116	3.2%	1.30 [0.83, 2.05]
Subtotal (95% CI)		1808		1689	15.5%	1.32 [0.98, 1.77]
Total events	195		152			
Heterogeneity: $Tau^2 = 0.05$; $Chi^2 = 12.84$, df = 9 (P = 0.17); $I^2 = 30\%$						
Test for overall effect: Z = 1.86 (P = 0.06)						
**1.13.2 Functional iron deficiency**						
Agarwal 2015	37	67	27	69	3.8%	1.41 [0.98, 2.03]
Anker 2009	50	305	24	254	3.2%	1.73 [1.10, 2.74]
Bailie 2010	27	559	22	559	2.6%	1.23 [0.71, 2.13]
Bisbe 2014	4	60	0	62	0.2%	9.30 [0.51, 169.01]
Coyne 2007	13	145	8	145	1.5%	1.63 [0.69, 3.80]
Kalra 2016	56	112	53	117	4.6%	1.10 [0.84, 1.45]
Khalafallah 2016	2	103	13	98	0.6%	0.15 [0.03, 0.63]
Kulnigg 2008	18	137	6	63	1.4%	1.38 [0.58, 3.31]
Lindgren 2009	12	45	4	46	1.0%	3.07 [1.07, 8.80]
MacDougall 2014	114	304	111	312	5.1%	1.05 [0.86, 1.30]
Michael 2002	3	1240	2	1250	0.4%	1.51 [0.25, 9.03]
Okonko 2008	2	24	3	11	0.5%	0.31 [0.06, 1.58]
Ponikowski 2020	102	559	121	551	4.9%	0.83 [0.66, 1.05]
Qunibi 2011	20	147	8	103	1.7%	1.75 [0.80, 3.82]
Singh 2006	9	75	9	75	1.4%	1.00 [0.42, 2.38]
Van Wyck 2005	2	79	0	82	0.1%	5.19 [0.25, 106.38]
Xu 2020	14	75	21	75	2.4%	0.67 [0.37, 1.21]
Subtotal (95% CI)		4036		3872	35.4%	1.15 [0.94, 1.41]
Total events	485		432			
Heterogeneity: $Tau^2 = 0.07$; $Chi^2 = 32.94$, df = 16 (P = 0.008); $I^2 = 51\%$						
Test for overall effect: Z = 1.32 (P = 0.19)						
**1.13.3 Anaemia only**						
Bastit 2008	18	200	39	196	2.8%	0.45 [0.27, 0.76]
Bernabeu–Wittel 2016	20	203	9	100	1.8%	1.09 [0.52, 2.32]
Breymann 2008	19	277	4	177	1.0%	3.04 [1.05, 8.77]
Hedenus 2007	18	33	6	34	1.6%	3.09 [1.40, 6.81]
Henry 2007	6	41	16	88	1.4%	0.80 [0.34, 1.91]
Johansson 2015	12	30	7	30	1.7%	1.71 [0.78, 3.75]
Keeler 2017	36	55	31	61	4.3%	1.29 [0.94, 1.76]
Kim 2017	1	228	0	226	0.1%	2.97 [0.12, 72.62]
Litton 2016	22	70	16	70	2.6%	1.38 [0.79, 2.39]
Meyer 1996	2	21	2	21	0.4%	1.00 [0.16, 6.45]
Neogi 2019	13	934	13	947	1.7%	1.01 [0.47, 2.18]
Padmanabhan 2019	4	20	5	20	0.9%	0.80 [0.25, 2.55]
Pedrazzoli 2008	1	73	1	76	0.2%	1.04 [0.07, 16.34]
Pieracci 2014	44	75	52	75	4.8%	0.85 [0.66, 1.08]
Richards 2020	7	234	15	234	1.4%	0.47 [0.19, 1.12]
Trenkwalder 2017	11	58	8	52	1.5%	1.23 [0.54, 2.83]
Vadhan–Raj 2013	33	608	10	200	2.0%	1.09 [0.54, 2.16]
Verma 2011	1	75	0	75	0.1%	3.00 [0.12, 72.49]
Subtotal (95% CI)		3235		2682	30.3%	1.09 [0.85, 1.39]
Total events	268		234			
Heterogeneity: $Tau^2 = 0.10$; $Chi^2 = 32.82$, df = 17 (P = 0.01); $I^2 = 48\%$						
Test for overall effect: Z = 0.68 (P = 0.50)						
**1.13.4 Non-anaemic iron deficiency**						
Burns 1996	4	11	3	12	0.8%	1.45 [0.42, 5.10]
Grote 2009	1	29	0	31	0.1%	3.20 [0.14, 75.55]
Krayenbuehl 2011	10	43	6	47	1.3%	1.82 [0.72, 4.59]
Subtotal (95% CI)		83		90	2.2%	1.74 [0.84, 3.59]
Total events	15		9			
Heterogeneity: $Tau^2 = 0.00$; $Chi^2 = 0.23$, df = 2 (P = 0.89); $I^2 = 0\%$						
Test for overall effect: Z = 1.50 (P = 0.13)						
**1.13.5 Non-anaemic**						
Bencalova 2009	9	130	1	130	0.3%	9.00 [1.16, 70.03]
Evstatiev 2013	20	105	9	100	1.8%	2.12 [1.01, 4.42]
Fishbane 1995	2	25	5	50	0.5%	0.80 [0.17, 3.84]
Mudge 2012	10	51	12	51	1.8%	0.83 [0.40, 1.75]
Pollak 2001	2	10	4	19	0.6%	0.95 [0.21, 4.32]
Santer 2020	7	24	7	24	1.4%	1.00 [0.41, 2.42]
Subtotal (95% CI)		345		374	6.4%	1.29 [0.75, 2.23]
Total events	50		38			
Heterogeneity: $Tau^2 = 0.15$; $Chi^2 = 7.67$, df = 5 (P = 0.18); $I^2 = 35\%$						
Test for overall effect: Z = 0.93 (P = 0.35)						
**1.13.6 Unclear**						
Allen 2011	3	24	2	21	0.5%	1.31 [0.24, 7.12]
Beguin 2013	10	50	7	52	1.4%	1.49 [0.61, 3.60]
Bielza 2020	17	126	17	127	2.2%	1.01 [0.54, 1.88]
Friel 1995	9	14	7	12	2.3%	1.10 [0.59, 2.04]
Kuo 2008	2	55	1	55	0.2%	2.00 [0.19, 21.42]
Moppett 2019	8	39	12	41	1.7%	0.70 [0.32, 1.53]
Serrano–Trenas 2011	16	100	13	199	2.0%	2.45 [1.23, 4.89]
Subtotal (95% CI)		408		507	10.3%	1.24 [0.89, 1.73]
Total events	65		59			
Heterogeneity: $Tau^2 = 0.02$; $Chi^2 = 6.70$, df = 6 (P = 0.35); $I^2 = 10\%$						
Test for overall effect: Z = 1.28 (P = 0.20)						
**Total (95% CI)**		9915		9214	100.0%	1.18 [1.05, 1.33]
Total events	1078		924			
Heterogeneity: $Tau^2 = 0.06$; $Chi^2 = 98.70$, df = 60 (P = 0.001); $I^2 = 39\%$						
Test for overall effect: Z = 2.71 (P = 0.007)						
Test for subgroup differences: $Chi^2 = 2.35$, df = 5 (P = 0.80), $I^2 = 0\%$						

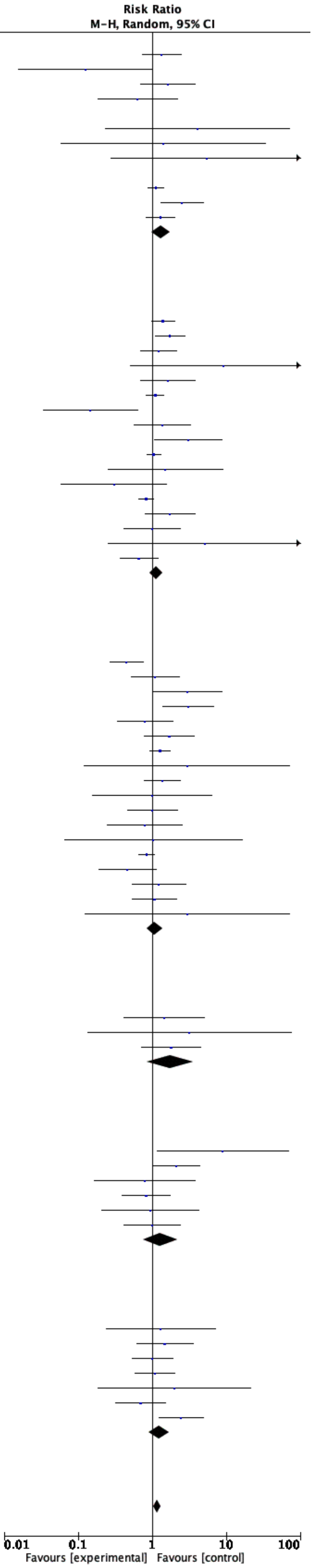

© 2021 Shah AA et al. *JAMA Network Open.*

eFigure 6. Secondary outcomes - hemoglobin forest plots

(a) Difference in haemoglobin at primary outcome timepoint or study endpoint

Study or Subgroup	Experimental Mean	Experimental SD	Experimental Total	Control Mean	Control SD	Control Total	Weight	Mean Difference IV, Random, 95% CI
**1.4.1 IV iron vs. oral iron**								
Westad 2008	11.9	1.4	58	12.3	1.3	70	0.8%	−0.40 [−0.87, 0.07]
Weisbach 1999	12.7	1	20	12.8	0.9	23	0.7%	−0.10 [−0.67, 0.47]
Warady 2004	11.2	1.32	17	11.44	0.84	18	0.5%	−0.24 [−0.98, 0.50]
Verma 2011	11.5	0.76	75	10.09	1.02	75	1.0%	1.41 [1.12, 1.70]
Steensma 2011	12.5	1.55	164	12.3	1.61	163	1.0%	0.20 [−0.14, 0.54]
Spinowitz 2008	10.88	1.27	228	10.15	1.07	76	1.0%	0.73 [0.44, 1.02]
Singh 1998	11	1	50	9.9	2	50	0.6%	1.10 [0.48, 1.72]
Seid 2017	12.18	1.06	1023	11.55	1.3	1022	1.2%	0.63 [0.53, 0.73]
Seid 2008	12.9	1.06	139	12.3	1.09	147	1.1%	0.60 [0.35, 0.85]
Rudra 2016	11.48	0.61	100	10.9	0.62	100	1.2%	0.58 [0.41, 0.75]
Reinisch 2013	12.23	1.33	219	12.59	1.91	108	0.9%	−0.36 [−0.76, 0.04]
Rathod 2015	11.76	1.01	200	10.36	1.39	100	1.0%	1.40 [1.09, 1.71]
Qunibi 2011	11.19	1.11	147	10.75	1.18	103	1.0%	0.44 [0.15, 0.73]
Provenzano 2009	11.41	1.23	114	11.09	1.07	116	1.0%	0.32 [0.02, 0.62]
Perello 2014	12.2	0.9	36	12.2	1	36	0.8%	0.00 [−0.44, 0.44]
Padmanabhan 2019	12.01	0.98	22	11.83	1.2	22	0.6%	0.18 [−0.47, 0.83]
Onken 2014	12.16	1.11	246	11.42	1.16	253	1.2%	0.74 [0.54, 0.94]
Olijhoek 2001	12.5	0.7	25	12.5	0.7	27	0.9%	0.00 [−0.38, 0.38]
Noronha 2018	10	1.48	94	9.7	1.36	98	0.9%	0.30 [−0.10, 0.70]
Neogi 2019	10.5	1.3	666	9.6	1.7	629	1.2%	0.90 [0.73, 1.07]
Neeru 2012	11.24	0.35	50	11.06	0.32	50	1.2%	0.18 [0.05, 0.31]
Montano−Pedroso 2018	12.8	0.81	27	12.54	1.18	28	0.7%	0.26 [−0.27, 0.79]
McMahon 2010	12.1	1	43	11.7	3	42	0.4%	0.40 [−0.56, 1.36]
MacDougall 2014	12	0.1	153	11.5	0.1	308	1.3%	0.50 [0.48, 0.52]
Maccio 2010	11.4	1.6	73	11.6	1.2	75	0.8%	−0.20 [−0.66, 0.26]
Li 2008	12.01	1.26	70	10.02	1.08	66	0.9%	1.99 [1.60, 2.38]
Kochhar 2013	12.8	1.1	50	10.7	0.7	50	0.9%	2.10 [1.74, 2.46]
Kim 2009	10.5	1.4	30	8.6	1.4	26	0.5%	1.90 [1.16, 2.64]
Khalafallah 2018 (2)	12.26	0.7	77	11.95	0.61	76	1.1%	0.31 [0.10, 0.52]
Khalafallah 2018 (1)	12.22	0.89	83	11.95	0.61	76	1.1%	0.27 [0.03, 0.51]
Khalafallah 2010	12.66	0.97	92	12.18	0.87	91	1.1%	0.48 [0.21, 0.75]
Keeler 2017	13.23	1.66	55	12.59	1.51	61	0.7%	0.64 [0.06, 1.22]
Kasper 1998	11.8	1.2	41	12.2	1.2	45	0.8%	−0.40 [−0.91, 0.11]
Jain 2013	9.1	0.4	21	8	0.3	20	1.1%	1.10 [0.88, 1.32]
Iyoke 2017	10.86	0.53	115	10.77	0.57	122	1.2%	0.09 [−0.05, 0.23]
Henry 2007	12.5	1.1	41	11.9	1.7	44	0.6%	0.60 [−0.00, 1.20]
Gupta 2014	11.5	0.78	50	10.85	1.12	50	0.9%	0.65 [0.27, 1.03]
Ferrer−Barcelo 2019	14.4	2.6	29	13.1	2.6	31	0.2%	1.30 [−0.02, 2.62]
El Khouly 2017	11.5	0.43	162	10.15	0.59	154	1.2%	1.35 [1.24, 1.46]
Dubey 2013	10.8	1.03	100	9.87	0.86	100	1.1%	0.93 [0.67, 1.19]
Deeba 2012	10.79	0.8	100	9.9	0.88	100	1.1%	0.89 [0.66, 1.12]
Darwish 2019	10.29	0.86	33	9.51	0.77	33	0.9%	0.78 [0.39, 1.17]
Dangsuwan 2010	10	0.8	22	9.5	0.9	22	0.8%	0.50 [−0.00, 1.00]
Damineni 2016	11.93	0.87	45	11.15	0.74	45	1.0%	0.78 [0.45, 1.11]
Dalal 2018	10.16	0.73	75	9.05	0.9	75	1.1%	1.11 [0.85, 1.37]
Charytan 2012	11.08	0.92	246	10.78	0.88	240	1.2%	0.30 [0.14, 0.46]
Charytan 2005	11.1	0.8	48	10.7	0.6	48	1.0%	0.40 [0.12, 0.68]
Breymann 2016	11.55	1.18	123	10.32	1.54	124	1.0%	1.23 [0.89, 1.57]
Breymann 2008	13	1.2	179	12.8	1.3	89	1.0%	0.20 [−0.12, 0.52]
Bisbe 2014	11.5	1.2	59	11	1.1	62	0.9%	0.50 [0.09, 0.91]
Birgegard 2016	11.58	1.58	225	11.54	1.64	112	0.9%	0.04 [−0.33, 0.41]
Biboulet 2018	14.9	0.75	50	13.9	0.95	50	1.0%	1.00 [0.66, 1.34]
Bhavi 2017	10.64	1.3	56	10.65	1.03	56	0.8%	−0.01 [−0.44, 0.42]
Bhandal 2006	11.5	1.3	22	11.2	1.2	21	0.5%	0.30 [−0.45, 1.05]
Bencalova 2009	12.2	0.9	130	12.4	1.1	130	1.1%	−0.20 [−0.44, 0.04]
Beck−da−Silva 2013	12.04	0.6	10	12.99	0.5	7	0.7%	−0.95 [−1.47, −0.43]
Bayoumeu 2002	11.11	1.3	24	11	1.25	23	0.5%	0.11 [−0.62, 0.84]
Bager 2014	13.9	0.45	42	13.5	0.6	41	1.1%	0.40 [0.17, 0.63]
Auerbach 2004	12.2	0.8	37	11.2	0.7	43	1.0%	1.00 [0.67, 1.33]
Athibovonsuk 2013	11	0.2	32	10.6	0.1	32	1.3%	0.40 [0.32, 0.48]
Al−Momen 1996	12.85	0.66	52	11.14	1.24	59	0.9%	1.71 [1.35, 2.07]
Al 2005	10.7	0.6	45	10	0.5	45	1.1%	0.70 [0.47, 0.93]
Akhtar 2018	12.89	0.79	30	11.4	0.39	30	1.0%	1.49 [1.17, 1.81]
Aggarwal 2003	10.05	0.9	20	8.94	1.17	20	0.6%	1.11 [0.46, 1.76]
Agarwal 2015	11.39	0.51	67	11.11	0.51	67	1.2%	0.28 [0.11, 0.45]
Agarwal 2006	11.3	0.8	36	10.9	0.9	39	0.9%	0.40 [0.02, 0.78]
Adhikary 2011	9.87	1.09	45	9.82	1.02	45	0.8%	0.05 [−0.39, 0.49]
Abhilashini 2014	10.84	0.9	50	10.09	0.7	50	1.0%	0.75 [0.43, 1.07]
Abdelazim 2017	11.7	0.9	126	11.3	1.3	124	1.1%	0.40 [0.12, 0.68]
Subtotal (95% CI)			7034			6583	64.2%	0.57 [0.48, 0.66]
Heterogeneity: $Tau^2 = 0.12$; $Chi^2 = 908.29$, df = 68 (P < 0.00001); $I^2 = 93\%$								
Test for overall effect: Z = 12.04 (P < 0.00001)								
**1.4.2 IV iron vs. no iron/usual care**								
Yoo 2011	9.4	0.9	37	8.3	1.1	37	0.8%	1.10 [0.64, 1.56]
Xu 2020	11.37	1.46	75	10.82	1.47	73	0.8%	0.55 [0.08, 1.02]
Woods 2014	14.1	1.1	7	14.3	1	7	0.3%	−0.20 [−1.30, 0.90]
Weisbach 1999	12.7	1	20	12.5	1.6	17	0.4%	0.20 [−0.68, 1.08]
Van Iperen 2000	11.4	1.3	9	10.8	0.8	9	0.3%	0.60 [−0.40, 1.60]
Vadhan−Raj 2013	11.4	1.5	608	9	0.9	200	1.2%	2.40 [2.23, 2.57]
Toblli 2007	11.8	0.7	20	9.8	0.6	20	0.9%	2.00 [1.60, 2.40]
Serrano−Trenas 2011	10.3	1.3	100	10.5	1.3	100	0.9%	−0.20 [−0.56, 0.16]
Sedighi 2013	10.59	1.2	20	9.97	1.5	20	0.4%	0.62 [−0.22, 1.46]
Santer 2020	14.45	1.23	24	14.13	1.45	24	0.5%	0.32 [−0.44, 1.08]
Price 2014	11.19	0.46	9	10.91	0.85	10	0.6%	0.28 [−0.33, 0.89]
Ponikowski 2020	13.1	0.8	339	12.4	1.7	346	1.2%	0.70 [0.50, 0.90]
Park 2019	12.8	1.3	29	12.6	1.1	29	0.6%	0.20 [−0.42, 0.82]
Okonko 2008	13.2	1.1	20	12.6	1.1	10	0.4%	0.60 [−0.23, 1.43]
Ng 2018	10.6	1.19	7	10.7	1.49	10	0.2%	−0.10 [−1.38, 1.18]
Moppett 2019	4.64	0.09	39	4.61	0.14	41	1.3%	0.03 [−0.02, 0.08]
Litton 2016	10.67	1.38	70	10.15	1.5	70	0.8%	0.52 [0.04, 1.00]
Kuo 2008	10.5	1.2	45	10	1.9	44	0.6%	0.50 [−0.16, 1.16]
Kulnigg−Dabsch 2013	13.1	1.2	16	11	0.8	9	0.5%	2.10 [1.31, 2.89]
Krayenbuehl 2011	13.3	0.6	43	13.3	0.7	47	1.1%	0.00 [−0.27, 0.27]
Kim 2007	10.77	1.32	30	11.23	1.29	45	0.6%	−0.46 [−1.06, 0.14]
Khalafallah 2016	13.75	1.11	103	13.36	1.13	98	1.0%	0.39 [0.08, 0.70]
Keller 2020	14.27	1.09	203	13.72	1.2	202	1.1%	0.55 [0.33, 0.77]
Kasper 1998	11.8	1.2	41	11.5	1	22	0.7%	0.30 [−0.26, 0.86]
Karkouti 2006	12.4	0.7	11	11.5	1.5	10	0.3%	0.90 [−0.12, 1.92]
Johansson 2015	12.4	0.5	30	11.6	0.7	30	1.0%	0.80 [0.49, 1.11]
Henry 2007	12.5	1.1	41	12	1.2	44	0.8%	0.50 [0.01, 0.99]
Hedenus 2007	13	1.76	33	11.8	1.78	43	0.5%	1.20 [0.40, 2.00]
Grote 2009	13.45	1.18	29	13.25	1.1	31	0.7%	0.20 [−0.38, 0.78]
Garrido−Martin 2012	11.05	1.4	107	11	1.44	52	0.8%	0.05 [−0.42, 0.52]
Froessler 2016	12.2	1.2	36	11.1	1.2	28	0.7%	1.10 [0.51, 1.69]
Edwards 2009	13.4	2.2	34	13.7	2.6	26	0.2%	−0.30 [−1.54, 0.94]
Deng 2017	11.94	0.36	16	10.87	0.38	16	1.1%	1.07 [0.81, 1.33]
Coyne 2007	11.9	1.3	64	11.3	1.4	65	0.8%	0.60 [0.13, 1.07]
Charles−Edwards 2019	13	1.3	21	12.7	1.4	19	0.4%	0.30 [−0.54, 1.14]
Burns 1996	9.4	0.4	11	10.1	0.4	12	1.0%	−0.70 [−1.03, −0.37]
Burden 2015	13.72	1.38	7	13.87	1.17	8	0.2%	−0.15 [−1.45, 1.15]
Boomershine 2018	13.5	0.9	41	12.2	0.9	40	0.9%	1.30 [0.91, 1.69]
Bernabeu−Wittel 2016	12.36	1.5	103	11.9	1.3	100	0.9%	0.46 [0.07, 0.85]
Beck−da−Silva 2013	12.04	0.6	10	12	0.7	6	0.6%	0.04 [−0.63, 0.71]
Barish (2) 2012	11.95	1.13	366	11.85	1.05	369	1.2%	0.10 [−0.06, 0.26]
Barish (1) 2012	12.1	1.1	343	11.85	1.1	360	1.2%	0.25 [0.09, 0.41]
Bager 2014	13.9	0.45	42	11.5	1.2	14	0.6%	2.40 [1.76, 3.04]
Auerbach 2010	11.8	1.1	116	11.5	0.8	122	1.1%	0.30 [0.05, 0.55]
Auerbach 2004	12.2	0.8	37	10.5	0.9	36	0.9%	1.70 [1.31, 2.09]
Anker 2009	13	0.1	305	12.5	0.1	154	1.3%	0.50 [0.48, 0.52]
Subtotal (95% CI)			3717			3075	34.6%	0.58 [0.42, 0.74]
Heterogeneity: $Tau^2 = 0.22$; $Chi^2 = 1105.80$, df = 45 (P < 0.00001); $I^2 = 96\%$								
Test for overall effect: Z = 7.17 (P < 0.00001)								
**1.4.3 IV iron vs. IM iron**								
Nanthini 2017	11.38	0.5	65	11.16	0.48	62	1.2%	0.22 [0.05, 0.39]
Subtotal (95% CI)			65			62	1.2%	0.22 [0.05, 0.39]
Heterogeneity: Not applicable								
Test for overall effect: Z = 2.53 (P = 0.01)								
Total (95% CI)			10816			9720	100.0%	0.57 [0.50, 0.64]
Heterogeneity: $Tau^2 = 0.10$; $Chi^2 = 2048.80$, df = 115 (P < 0.00001); $I^2 = 94\%$								
Test for overall effect: Z = 16.26 (P < 0.00001)								
Test for subgroup differences: $Chi^2 = 13.47$, df = 2 (P = 0.001), $I^2 = 85.2\%$								

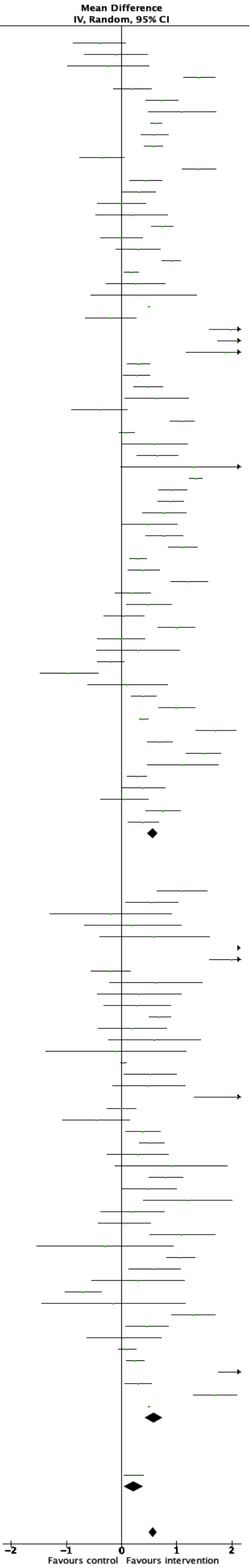

© 2021 Shah AA et al. *JAMA Network Open.*

(b) By treatment response, as defined by the study authors

Study or Subgroup	Experimental Events	Experimental Total	Control Events	Control Total	Weight	Risk Ratio M-H, Random, 95% CI
**1.5.1 IV iron vs. oral iron**						
Adhikary 2011	27	45	11	45	1.3%	2.45 [1.39, 4.32]
Auerbach 2004	53	78	15	43	1.5%	1.95 [1.26, 3.01]
Bager 2014	38	42	31	41	2.1%	1.20 [0.98, 1.46]
Batool 2018	36	41	27	41	2.0%	1.33 [1.04, 1.71]
Bayoumeu 2002	3	24	4	23	0.4%	0.72 [0.18, 2.87]
Beguin 2013	23	23	20	25	2.0%	1.24 [1.01, 1.53]
Bencalova 2009	97	130	104	130	2.2%	0.93 [0.82, 1.06]
Bisbe 2014	20	59	13	62	1.2%	1.62 [0.89, 2.95]
Breymann 2008	152	179	73	89	2.2%	1.04 [0.92, 1.16]
Breymann 2016	103	124	87	123	2.2%	1.17 [1.02, 1.35]
Charytan 2005	26	48	15	48	1.4%	1.73 [1.06, 2.84]
Charytan 2012	64	249	55	249	1.8%	1.16 [0.85, 1.59]
Dubey 2013	62	100	5	100	0.8%	12.40 [5.21, 29.54]
Evstatiev 2013	77	105	60	99	2.1%	1.21 [0.99, 1.47]
Ferrer–Barcelo 2019	29	39	19	31	1.8%	1.21 [0.87, 1.70]
Henry 2007	30	41	20	44	1.7%	1.61 [1.11, 2.34]
Iyoke 2017	110	115	115	122	2.3%	1.01 [0.96, 1.08]
Khalafallah 2010	15	92	26	91	1.3%	0.57 [0.32, 1.00]
Kim 2009	23	30	3	26	0.6%	6.64 [2.25, 19.61]
Kulnigg 2008	36	136	2	60	0.4%	7.94 [1.98, 31.92]
Li 2008	45	70	19	66	1.6%	2.23 [1.47, 3.39]
Lindgren 2009	30	45	22	46	1.7%	1.39 [0.97, 2.01]
Maccio 2010	37	73	42	75	1.8%	0.91 [0.67, 1.22]
MacDougall 2014	139	305	99	308	2.1%	1.42 [1.16, 1.74]
McMahon 2010	34	43	30	42	2.0%	1.11 [0.87, 1.41]
Neogi 2019	240	666	128	629	2.1%	1.77 [1.47, 2.13]
Noronha 2018	20	87	16	90	1.2%	1.29 [0.72, 2.33]
Onken 2014	139	244	73	251	2.0%	1.96 [1.57, 2.45]
Padmanabhan 2019	1	22	2	22	0.2%	0.50 [0.05, 5.12]
Provenzano 2009	56	114	29	116	1.7%	1.96 [1.36, 2.83]
Qunibi 2011	89	147	36	103	1.9%	1.73 [1.29, 2.32]
Rathod 2015	93	200	12	100	1.3%	3.88 [2.23, 6.72]
Razzaq 2017	36	41	27	41	2.0%	1.33 [1.04, 1.71]
Schijns 2020	26	33	18	26	1.8%	1.14 [0.83, 1.55]
Schroder 2005	12	22	13	24	1.3%	1.01 [0.59, 1.71]
Seid 2008	127	139	98	147	2.2%	1.37 [1.21, 1.55]
Spinowitz 2008	89	228	14	76	1.4%	2.12 [1.29, 3.49]
Steensma 2011	114	164	109	163	2.2%	1.04 [0.90, 1.21]
Stoves 2001	15	22	14	23	1.5%	1.12 [0.73, 1.73]
Van Wyck 2005	35	79	23	82	1.6%	1.58 [1.03, 2.42]
Van Wyck 2007	163	169	158	168	2.3%	1.03 [0.98, 1.08]
Van Wyck 2009	187	228	139	225	2.2%	1.33 [1.18, 1.50]
Vanobberghen 2020	75	94	47	92	2.0%	1.56 [1.25, 1.95]
Weisbach 1999	6	30	5	60	0.6%	2.40 [0.80, 7.23]
**Subtotal (95% CI)**		4965		4467	71.5%	1.41 [1.27, 1.56]
Total events	2832		1878			
Heterogeneity: Tau^2 = 0.09; Chi^2 = 454.74, df = 43 ($P < 0.00001$); I^2 = 91%						
Test for overall effect: Z = 6.36 ($P < 0.00001$)						
**1.5.2 IV iron vs. no iron/comparator**						
Auerbach 2004	53	78	9	36	1.2%	2.72 [1.51, 4.88]
Auerbach 2010	70	81	56	72	2.1%	1.11 [0.96, 1.29]
Bastit 2008	172	200	143	196	2.2%	1.18 [1.06, 1.31]
Bielza 2020	82	126	92	127	2.1%	0.90 [0.76, 1.06]
Coyne 2007	30	64	19	65	1.5%	1.60 [1.01, 2.54]
Edwards 2009	9	34	9	26	0.9%	0.76 [0.35, 1.65]
Favrat 2014	144	144	118	146	2.2%	1.24 [1.14, 1.34]
Hedenus 2007	31	33	18	34	1.8%	1.77 [1.28, 2.46]
Hedenus 2014	8	8	6	9	1.4%	1.45 [0.90, 2.35]
Henry 2007	30	41	18	44	1.6%	1.79 [1.20, 2.67]
Kim 2007	9	30	21	45	1.1%	0.64 [0.34, 1.21]
Kim 2017	200	217	115	213	2.2%	1.71 [1.50, 1.94]
Pedrazzoli 2008	56	73	47	76	2.0%	1.24 [1.00, 1.54]
Richards 2020	42	244	21	243	1.4%	1.99 [1.22, 3.26]
Serrano–Trenas 2011	18	50	11	60	1.1%	1.96 [1.03, 3.76]
Singh 2006	39	66	11	33	1.3%	1.77 [1.05, 2.99]
Vadhan–Raj 2013	493	608	11	200	1.2%	14.74 [8.29, 26.22]
Xu 2020	18	75	8	73	0.9%	2.19 [1.02, 4.72]
**Subtotal (95% CI)**		2172		1698	28.5%	1.58 [1.28, 1.95]
Total events	1504		733			
Heterogeneity: Tau^2 = 0.16; Chi^2 = 244.36, df = 17 ($P < 0.00001$); I^2 = 93%						
Test for overall effect: Z = 4.26 ($P < 0.0001$)						
**Total (95% CI)**		7137		6165	100.0%	1.46 [1.32, 1.60]
Total events	4336		2611			
Heterogeneity: Tau^2 = 0.10; Chi^2 = 743.78, df = 61 ($P < 0.00001$); I^2 = 92%						
Test for overall effect: Z = 7.73 ($P < 0.00001$)						
Test for subgroup differences: Chi^2 = 0.95, df = 1 ($P = 0.33$), I^2 = 0%						

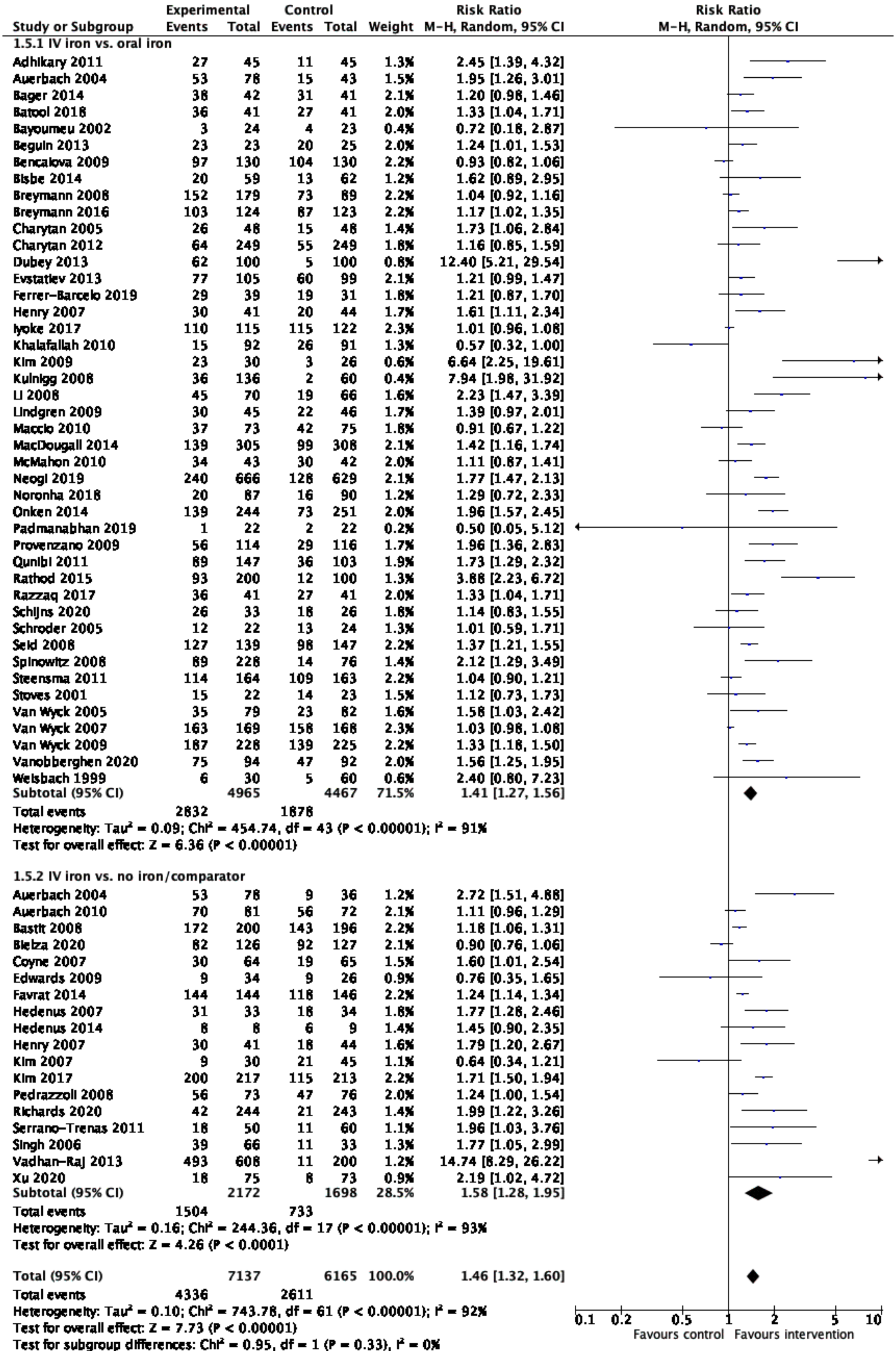

Association between changes in hemoglobin and intravenous iron. The mean differences and risk ratios were calculated using a random-effects model with Mantel-Haenszel weighting. The size of the data markers indicates the weight of the study. Error bars indicate 95% CIs.

© 2021 Shah AA et al. *JAMA Network Open.*

eFigure 7. Secondary outcomes – red blood cell (RBC) transfusion requirements

(a) Requirement for an RBC transfusion

Study or Subgroup	Experimental Events	Experimental Total	Control Events	Control Total	Weight	Risk Ratio M-H, Random, 95% CI
**1.5.1 IV iron vs. oral iron**						
Adhikary 2011	27	45	11	45	1.3%	2.45 [1.39, 4.32]
Auerbach 2004	53	78	15	43	1.6%	1.95 [1.26, 3.01]
Bager 2014	38	42	31	41	2.1%	1.20 [0.98, 1.46]
Batool 2018	36	41	27	41	2.0%	1.33 [1.04, 1.71]
Bayoumeu 2002	3	24	4	23	0.4%	0.72 [0.18, 2.87]
Beguin 2013	23	23	20	25	2.1%	1.24 [1.01, 1.53]
Bencaiova 2009	97	130	104	130	2.2%	0.93 [0.82, 1.06]
Bisbe 2014	20	59	13	62	1.2%	1.62 [0.89, 2.95]
Breymann 2008	152	179	73	89	2.2%	1.04 [0.92, 1.16]
Breymann 2016	103	124	87	123	2.2%	1.17 [1.02, 1.35]
Charytan 2005	26	48	15	48	1.4%	1.73 [1.06, 2.84]
Charytan 2012	64	249	55	249	1.9%	1.16 [0.85, 1.59]
Dubey 2013	62	100	5	100	0.8%	12.40 [5.21, 29.54]
Evstatiev 2013	77	105	60	99	2.1%	1.21 [0.99, 1.47]
Ferrer-Barcelo 2019	29	39	19	31	1.8%	1.21 [0.87, 1.70]
Henry 2007	30	41	20	44	1.7%	1.61 [1.11, 2.34]
Iyoke 2017	110	115	115	122	2.3%	1.01 [0.96, 1.08]
Khalafallah 2010	15	92	26	91	1.3%	0.57 [0.32, 1.00]
Kim 2009	23	30	3	26	0.6%	6.64 [2.25, 19.61]
Kulnigg 2008	36	136	2	60	0.4%	7.94 [1.98, 31.92]
Li 2008	45	70	19	66	1.6%	2.23 [1.47, 3.39]
Lindgren 2009	30	45	22	46	1.7%	1.39 [0.97, 2.01]
Maccio 2010	37	73	42	75	1.9%	0.91 [0.67, 1.22]
MacDougall 2014	139	305	99	308	2.1%	1.42 [1.16, 1.74]
McMahon 2010	34	43	30	42	2.0%	1.11 [0.87, 1.41]
Neogi 2019	240	666	128	629	2.1%	1.77 [1.47, 2.13]
Noronha 2018	20	87	16	90	1.2%	1.29 [0.72, 2.33]
Onken 2014	139	244	73	251	2.1%	1.96 [1.57, 2.45]
Padmanabhan 2019	1	22	2	22	0.2%	0.50 [0.05, 5.12]
Provenzano 2009	56	114	29	116	1.7%	1.96 [1.36, 2.83]
Qunibi 2011	89	147	36	103	1.9%	1.73 [1.29, 2.32]
Rathod 2015	93	200	12	100	1.3%	3.88 [2.23, 6.72]
Razzaq 2017	36	41	27	41	2.0%	1.33 [1.04, 1.71]
Schijns 2020	26	33	18	26	1.9%	1.14 [0.83, 1.55]
Schroder 2005	12	22	13	24	1.4%	1.01 [0.59, 1.71]
Seid 2008	127	139	98	147	2.2%	1.37 [1.21, 1.55]
Spinowitz 2008	89	228	14	76	1.4%	2.12 [1.29, 3.49]
Steensma 2011	114	164	109	163	2.2%	1.04 [0.90, 1.21]
Stoves 2001	15	22	14	23	1.6%	1.12 [0.73, 1.73]
Van Wyck 2005	35	79	23	82	1.6%	1.58 [1.03, 2.42]
Van Wyck 2007	163	169	158	168	2.3%	1.03 [0.98, 1.08]
Van Wyck 2009	187	228	139	225	2.2%	1.33 [1.18, 1.50]
Weisbach 1999	6	30	5	60	0.6%	2.40 [0.80, 7.23]
**Subtotal (95% CI)**		**4871**		**4375**	**70.9%**	**1.40 [1.26, 1.56]**
Total events	2757		1831			
Heterogeneity: Tau² = 0.09; Chi² = 446.50, df = 42 (P < 0.00001); I² = 91%						
Test for overall effect: Z = 6.21 (P < 0.00001)						
**1.5.2 IV iron vs. no iron/comparator**						
Auerbach 2004	53	78	9	36	1.2%	2.72 [1.51, 4.88]
Auerbach 2010	70	81	56	72	2.2%	1.11 [0.96, 1.29]
Bastit 2008	172	200	143	196	2.3%	1.18 [1.06, 1.31]
Bielza 2020	82	126	92	127	2.2%	0.90 [0.76, 1.06]
Coyne 2007	30	64	19	65	1.5%	1.60 [1.01, 2.54]
Edwards 2009	9	34	9	26	0.9%	0.76 [0.35, 1.65]
Favrat 2014	144	144	118	146	2.3%	1.24 [1.14, 1.34]
Hedenus 2007	31	33	18	34	1.8%	1.77 [1.28, 2.46]
Hedenus 2014	8	8	6	9	1.5%	1.45 [0.90, 2.35]
Henry 2007	30	41	18	44	1.7%	1.79 [1.20, 2.67]
Kim 2007	9	30	21	45	1.2%	0.64 [0.34, 1.21]
Kim 2017	200	217	115	213	2.2%	1.71 [1.50, 1.94]
Pedrazzoli 2008	56	73	47	76	2.1%	1.24 [1.00, 1.54]
Richards 2020	42	244	21	243	1.4%	1.99 [1.22, 3.26]
Serrano-Trenas 2011	18	50	11	60	1.1%	1.96 [1.03, 3.76]
Singh 2006	39	66	11	33	1.4%	1.77 [1.05, 2.99]
Vadhan-Raj 2013	493	608	11	200	1.3%	14.74 [8.29, 26.22]
Xu 2020	18	75	8	73	0.9%	2.19 [1.02, 4.72]
**Subtotal (95% CI)**		**2172**		**1698**	**29.1%**	**1.58 [1.28, 1.95]**
Total events	1504		733			
Heterogeneity: Tau² = 0.16; Chi² = 244.36, df = 17 (P < 0.00001); I² = 93%						
Test for overall effect: Z = 4.26 (P < 0.0001)						
**Total (95% CI)**		**7043**		**6073**	**100.0%**	**1.45 [1.32, 1.60]**
Total events	4261		2564			
Heterogeneity: Tau² = 0.10; Chi² = 739.13, df = 60 (P < 0.00001); I² = 92%						
Test for overall effect: Z = 7.61 (P < 0.00001)						
Test for subgroup differences: Chi² = 0.99, df = 1 (P = 0.32), I² = 0%						

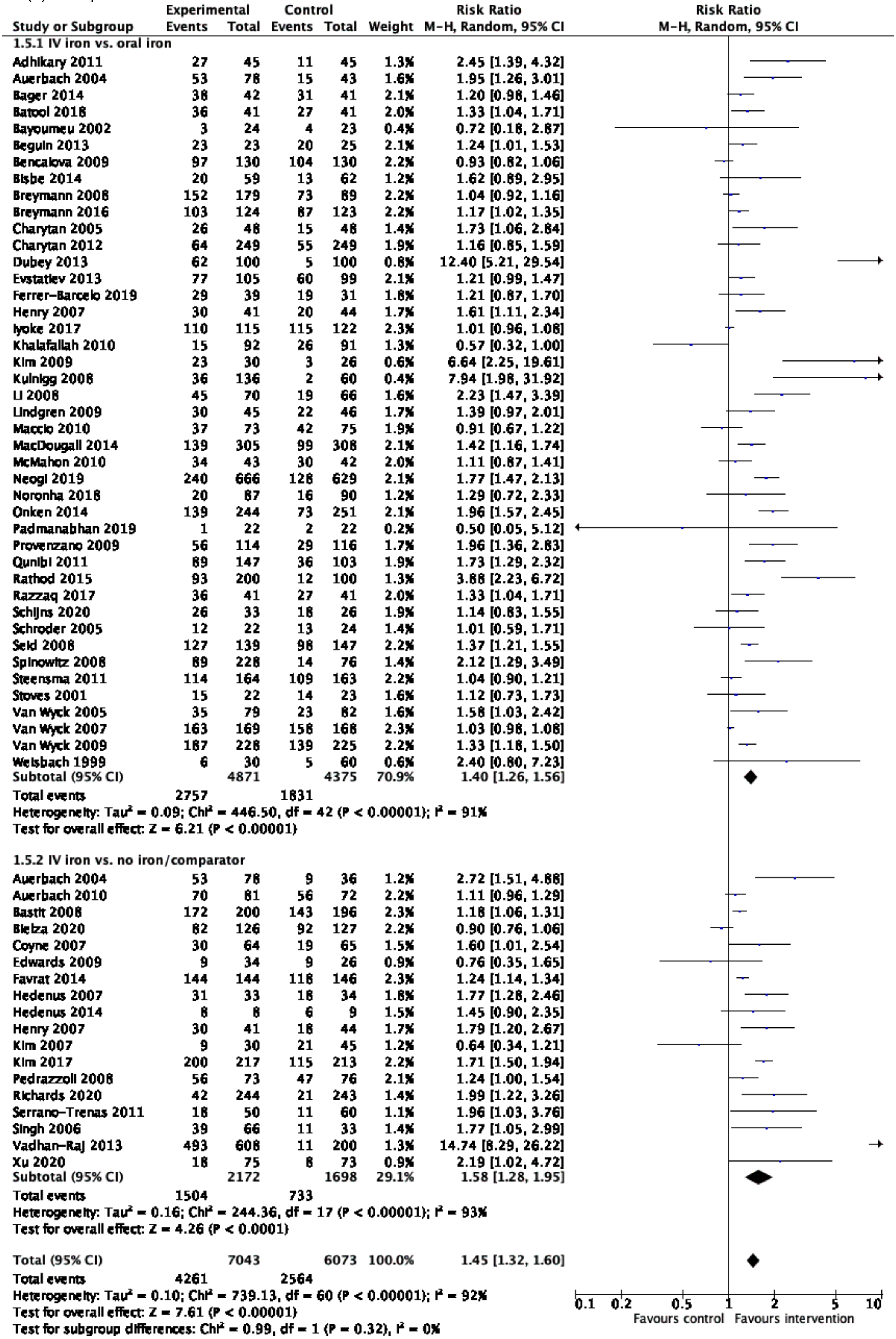

© 2021 Shah AA et al. *JAMA Network Open.*

(b) Mean number of RBCs transfused

Study or Subgroup	Experimental Mean	Experimental SD	Experimental Total	Control Mean	Control SD	Control Total	Weight	Mean Difference IV, Fixed, 95% CI
**1.6.1 IV iron vs. oral iron**								
Subtotal (95% CI)			0			0		Not estimable
Heterogeneity: Not applicable								
Test for overall effect: Not applicable								
**1.6.2 IV iron vs. no iron/usual care**								
Beguin 2013	0	0	23	0.6	1.9	25		Not estimable
Bernabeu-Wittel 2016	1.26	1.3	103	1.28	1.4	100	10.2%	−0.02 [−0.39, 0.35]
Garrido-Martin 2012	−0.085	0.74	107	−0.01	0.73	52	24.0%	−0.08 [−0.32, 0.17]
Johansson 2015	1.25	0.5	30	2.2	1.64	30	3.8%	−0.95 [−1.56, −0.34]
Kim 2007	1.87	2.7	30	3.58	3.89	45	0.6%	−1.71 [−3.20, −0.22]
Litton 2016	−0.27	1.1	70	0.016	1.15	70	10.2%	−0.29 [−0.66, 0.09]
Richards 2020	0.65	1.3	243	0.61	1.3	244	26.5%	0.04 [−0.19, 0.27]
Serrano-Trenas 2011	0.76	1.16	100	0.87	1.12	100	14.2%	−0.11 [−0.43, 0.21]
Spahn 2019	1.5	2.7	243	1.9	2.9	241	5.7%	−0.40 [−0.90, 0.10]
Van Iperen 2000	1.07	1.04	12	2.06	0.93	12	2.3%	−0.99 [−1.78, −0.20]
Yoo 2011	1.6	0.9	37	3.7	2.1	37	2.6%	−2.10 [−2.84, −1.36]
Subtotal (95% CI)			998			956	100.0%	−0.20 [−0.32, −0.08]
Heterogeneity: Chi^2 = 46.31, df = 9 (P < 0.00001); I^2 = 81%								
Test for overall effect: Z = 3.31 (P = 0.0009)								
**Total (95% CI)**			998			956	100.0%	−0.20 [−0.32, −0.08]
Heterogeneity: Chi^2 = 46.31, df = 9 (P < 0.00001); I^2 = 81%								
Test for overall effect: Z = 3.31 (P = 0.0009)								
Test for subgroup differences: Not applicable								

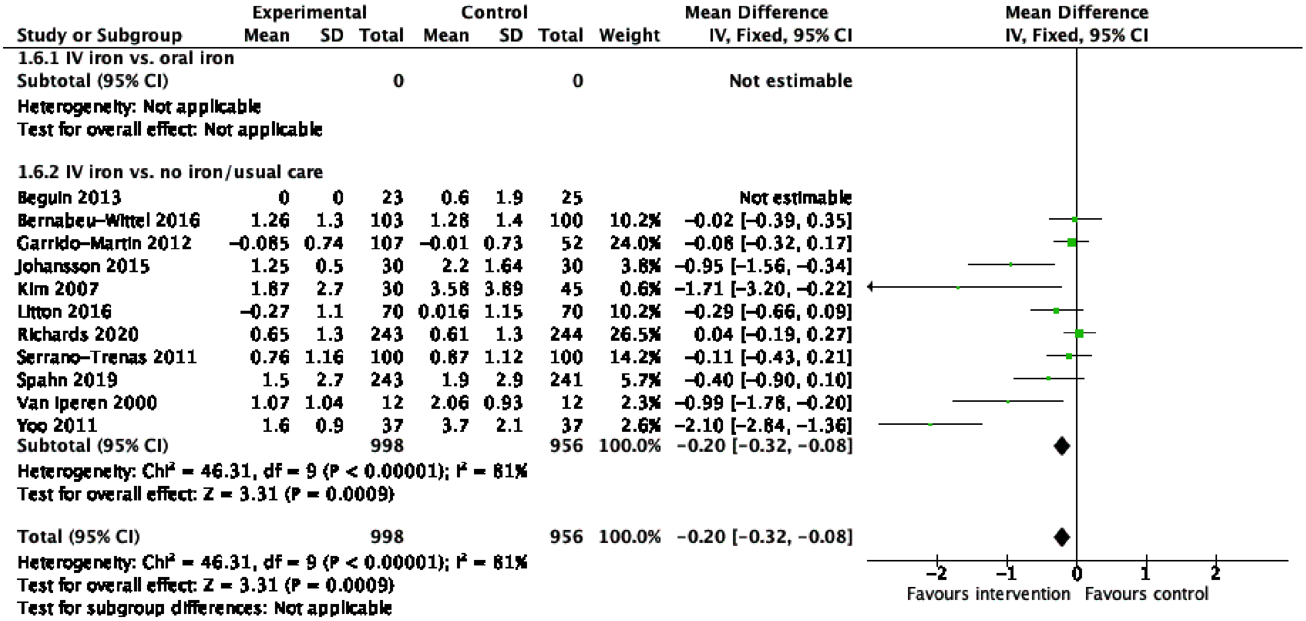

Association between transfusion requirements and intravenous iron. The mean differences and risk ratios were calculated using a random-effects model with Mantel-Haenszel weighting. The size of the data markers indicates the weight of the study. Error bars indicate 95% CIs

© 2021 Shah AA et al. *JAMA Network Open.*

eFigure 8. Secondary outcomes – mortality and hospital length of stay

(a) Short-term mortality (≤30 days)

Study or Subgroup	Experimental Events	Experimental Total	Control Events	Control Total	Weight	Risk Ratio M-H, Random, 95% CI
**1.2.1 IV iron vs. oral iron**						
Bielza 2020	7	126	7	127	12.5%	1.01 [0.36, 2.79]
Charytan 2012	0	246	2	240	1.4%	0.20 [0.01, 4.04]
Keeler 2017	5	55	4	61	8.2%	1.39 [0.39, 4.90]
Subtotal (95% CI)		427		428	22.1%	1.02 [0.47, 2.20]
Total events	12		13			
Heterogeneity: Tau^2 = 0.00; Chi^2 = 1.39, df = 2 (P = 0.50); I^2 = 0%						
Test for overall effect: Z = 0.05 (P = 0.96)						
**1.2.2 IV iron vs. no iron/usual care**						
Barish (2) 2012	0	366	1	369	1.3%	0.34 [0.01, 8.22]
Bernabeu-Wittel 2016	4	103	4	100	7.1%	0.97 [0.25, 3.78]
Froessler 2016	1	40	0	32	1.3%	2.41 [0.10, 57.35]
Litton 2016	7	70	6	70	12.1%	1.17 [0.41, 3.30]
Madi-Jebara 2004	0	40	0	40		Not estimable
Moppett 2019	4	39	0	41	1.6%	9.45 [0.53, 169.95]
Ng 2018	5	11	2	13	6.4%	2.95 [0.71, 12.34]
Richards 2020	2	237	2	237	3.4%	1.00 [0.14, 7.04]
Serrano-Trenas 2011	11	100	10	100	19.8%	1.10 [0.49, 2.47]
Spahn 2019	4	243	7	241	8.8%	0.57 [0.17, 1.91]
Van Iperen 2000	4	12	7	12	15.0%	0.57 [0.22, 1.45]
Yoo 2011	0	37	1	37	1.3%	0.33 [0.01, 7.93]
Subtotal (95% CI)		1298		1292	77.9%	0.99 [0.66, 1.48]
Total events	42		40			
Heterogeneity: Tau^2 = 0.00; Chi^2 = 8.20, df = 10 (P = 0.61); I^2 = 0%						
Test for overall effect: Z = 0.07 (P = 0.95)						
Total (95% CI)		1725		1720	100.0%	0.99 [0.69, 1.42]
Total events	54		53			
Heterogeneity: Tau^2 = 0.00; Chi^2 = 9.51, df = 13 (P = 0.73); I^2 = 0%						
Test for overall effect: Z = 0.04 (P = 0.97)						
Test for subgroup differences: Chi^2 = 0.01, df = 1 (P = 0.94), I^2 = 0%						

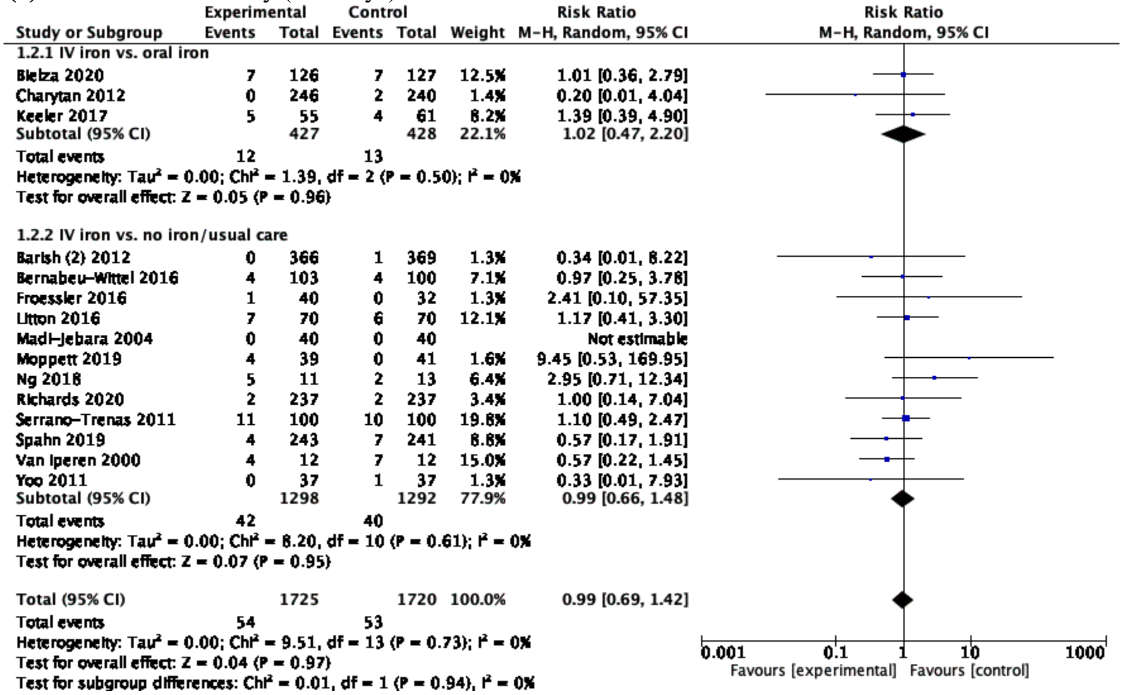

(b) Long-term mortality (>30 days)

Study or Subgroup	Experimental Events	Experimental Total	Control Events	Control Total	Weight	Odds Ratio M-H, Fixed, 95% CI
Anker 2009	5	305	4	154	3.4%	0.63 [0.17, 2.36]
Bastit 2008	21	200	15	196	8.9%	1.42 [0.71, 2.83]
Bielza 2020	12	126	16	127	9.5%	0.73 [0.33, 1.61]
Birgegard 2016	11	229	10	112	8.4%	0.51 [0.21, 1.25]
Kalra 2016	3	224	0	116	0.4%	3.68 [0.19, 71.88]
Onken 2014	0	246	2	253	1.6%	0.20 [0.01, 4.27]
Ponikowski 2015	12	152	14	152	8.5%	0.84 [0.38, 1.89]
Ponikowski 2020	77	558	78	550	44.6%	0.97 [0.69, 1.36]
Reinisch 2013	1	219	0	108	0.4%	1.49 [0.06, 36.87]
Spahn 2019	18	243	14	241	8.6%	1.30 [0.63, 2.67]
Steensma 2011	5	164	4	163	2.6%	1.25 [0.33, 4.74]
van Veldhuisen 2017	0	86	4	86	2.9%	0.11 [0.01, 2.00]
Total (95% CI)		2752		2258	100.0%	0.94 [0.75, 1.18]
Total events	165		161			
Heterogeneity: Chi^2 = 8.88, df = 11 (P = 0.63); I^2 = 0%						
Test for overall effect: Z = 0.56 (P = 0.58)						

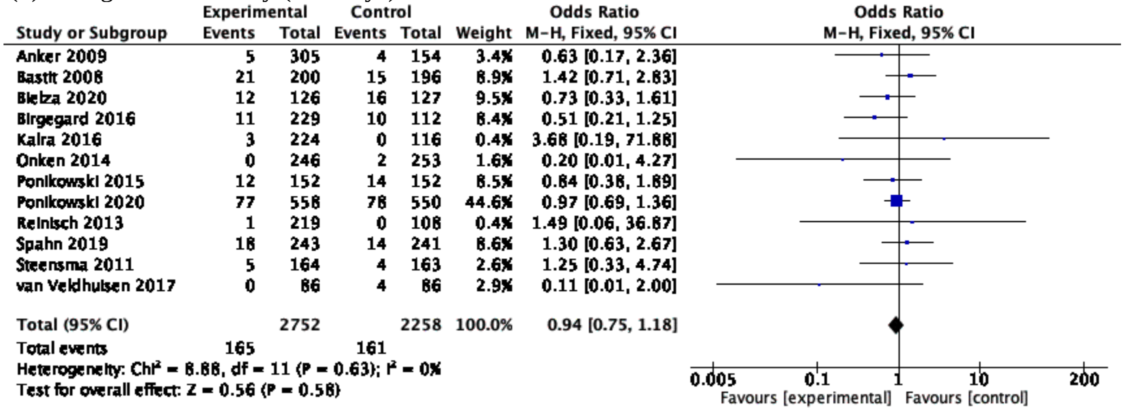

(c) Hospital length of stay

Study or Subgroup	Experimental Mean	Experimental SD	Experimental Total	Control Mean	Control SD	Control Total	Weight	Mean Difference IV, Random, 95% CI
**1.8.1 IV iron vs. oral iron**								
Bencalova 2009	5.6	2	55	5.8	1.9	139	29.3%	-0.20 [-0.82, 0.42]
Subtotal (95% CI)			55			139	29.3%	-0.20 [-0.82, 0.42]
Heterogeneity: Not applicable								
Test for overall effect: Z = 0.64 (P = 0.52)								
**1.8.2 IV iron vs. no iron/usual care**								
Khalafallah 2016	7.8	10.3	103	11.6	15.6	98	3.0%	-3.80 [-7.47, -0.13]
Kim 2017	10.7	7.9	228	10.9	13.8	226	8.2%	-0.20 [-2.27, 1.87]
Moppett 2019	2.48	0.59	39	2.56	0.48	41	37.3%	-0.08 [-0.32, 0.16]
Serrano-Trenas 2011	12.9	6.9	90	13.5	7.1	89	8.3%	-0.60 [-2.65, 1.45]
Spahn 2019	12	9.7	243	12.3	11.9	241	9.1%	-0.30 [-2.24, 1.64]
Van Iperen 2000	29	18	12	58	31	12	0.1%	-29.00 [-49.28, -8.72]
Yoo 2011	11.3	4.1	37	13.5	8	37	4.7%	-2.20 [-5.10, 0.70]
Subtotal (95% CI)			752			744	70.7%	-0.84 [-2.03, 0.34]
Heterogeneity: Tau^2 = 1.15; Chi^2 = 13.98, df = 6 (P = 0.03); I^2 = 57%								
Test for overall effect: Z = 1.39 (P = 0.16)								
Total (95% CI)			807			883	100.0%	-0.43 [-1.10, 0.24]
Heterogeneity: Tau^2 = 0.29; Chi^2 = 14.04, df = 7 (P = 0.05); I^2 = 50%								
Test for overall effect: Z = 1.27 (P = 0.20)								
Test for subgroup differences: Chi^2 = 0.89, df = 1 (P = 0.35), I^2 = 0%								

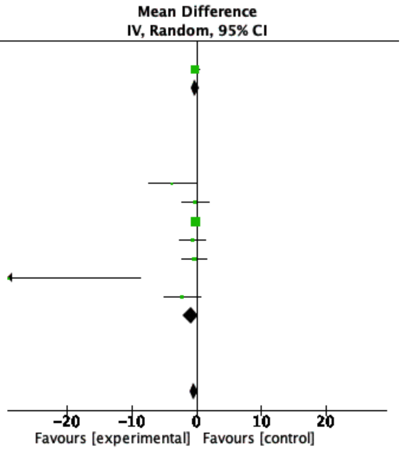

Association between mortality and hospital length of stay and intravenous iron. The mean differences and risk ratios were calculated using a random-effects model with Mantel-Haenszel weighting. The size of the data markers indicates the weight of the study. Error bars indicate 95% CIs

© 2021 Shah AA et al. *JAMA Network Open.*
